# Integrated Chemoenzymatic Synthesis of the mRNA Vaccine Building Block *N* ^1^‐Methylpseudouridine Triphosphate

**DOI:** 10.1002/anie.202506330

**Published:** 2025-07-04

**Authors:** Martin Pfeiffer, Leo Krammer, Johannes Zöhrer, Rolf Breinbauer, Bernd Nidetzky

**Affiliations:** ^1^ Institute of Biotechnology and Biochemical Engineering Graz University of Technology Petersgasse 12/1 Graz 8010 Austria; ^2^ Institute of Organic Chemistry Graz University of Technology Stremayrgasse 9/Z4 Graz 8010 Austria; ^3^ Austrian Centre of Industrial Biotechnology (acib) Krenngasse 37 Graz 8010 Austria; ^4^ BioTechMed‐Graz Graz 8010 Austria

**Keywords:** Biocatalysis, Enzyme catalysis, Nucleosides, Pseudouridine, Sustainable chemistry

## Abstract

Pseudouridine‐5′‐triphosphate (ΨTP) and its *N* ^1^‐methylated derivative (m^1^ΨTP) are critical monomer building blocks of mRNA therapeutics, yet efficient, scalable methods of their synthesis from readily accessible substrates remain underdeveloped. m^1^ΨTP is a major cost factor of production of the current COVID‐19 vaccines. We herein report a notably atom‐economic and high‐yielding biocatalytic route toward ΨTP and present two chemoenzymatic routes for producing m^1^ΨTP at ∼200 mg scale of isolated compound. Biocatalytic cascade rearrangement of uridine delivered ΨMP or Ψ in high yields. Acetonide‐protected ΨMP was selectively *N* ^1^‐methylated using dimethyl sulfate and subsequently converted to the triphosphate through efficient kinase cascade reaction. *Saccharomyces cerevisiae* uridine 5′‐monophosphate kinase was shown for ATP‐dependent phosphorylation of m^1^ΨMP to m^1^ΨDP and m^1^ΨTP synthesis was catalyzed by *Escherichia coli* acetate kinase which also served to regenerate ATP by acetyl phosphate. Benchmarked against chemical route converting the enzymatically produced Ψ into m^1^ΨTP, the novel chemoenzymatic route from ΨMP offered improved metrics of reaction efficiency and sustainability. The synthetic ΨTP and m^1^ΨTP replaced UTP for mRNA synthesis by in vitro transcription. Overall, this study shows the productive integration of chemical methylation with enzymatic cascade reactions for C─C coupling and phosphorylation toward an efficient preparation of m^1^ΨTP.

## Graphical abstract

Pseudouridine‐5′‐triphosphate (ΨTP) and its *N* ^1^‐methylated derivative (m^1^ΨTP) are crucial components in mRNA vaccine technology. Here, an atom‐economic, high‐yielding biocatalytic route for ΨTP synthesis is presented. A new chemoenzymatic route for m^1^ΨTP synthesis was developed. The method offers enhanced reaction efficiency and sustainability compared to pure chemical synthesis.

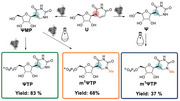

Pseudouridine (Ψ, **1**, Figure 1a) is the *C*‐nucleoside isomer of uridine (U, **2**). Ψ is found naturally in various types of RNA due to positional isomerization of certain U to Ψ by the biological machinery of Ψ‐synthases.^[^ ^1^, ^2^, ^3^ ^]^ Such U‐to‐Ψ substitutions are well‐known to enhance RNA stability through their effect on RNA secondary structure.^[^ ^4^ ^]^ Uniform substitution of U by Ψ, or the *N* ^1^‐methylated derivative thereof (m^1^Ψ, **3**), was key in the development of therapeutic applications of synthetic mRNA, including the vaccines against COVID‐19 in particular.^[^ ^5^, ^6^, ^7^, ^8^, ^9^, ^10^ ^]^ Fully Ψ‐ or m^1^Ψ‐substituted mRNA is less immunogenic and exhibits increased translational efficiency,^[^ ^8^, ^9^, ^10^ ^]^ in spite of the slightly reduced fidelity of mRNA translation caused by m^1^Ψ as was recently noted.^[^ ^11^, ^12^ ^]^ Ψ or m^1^Ψ are incorporated into synthetic mRNA from the corresponding 5′‐triphosphates (ΨTP, **1c**; m^1^ΨTP, **3c**) by in vitro transcription (IVT, Figure 1b).^[^ ^5^, ^6^, ^7^, ^8^, ^9^, ^10^ ^]^ The promiscuous activity of the RNA polymerase allows for ΨTP or m^1^ΨTP to be used instead of UTP. Future RNA therapeutics beyond the vaccines will target diseases such as cancer, thus necessitating long‐term and more frequent RNA‐based medicine administration.^[^ ^13^, ^14^, ^15^ ^]^ Consequently, RNA production will surge, driving proportional demand for nucleotide monomer building blocks. The supply of m^1^ΨTP for IVT represents the second highest cost in mRNA vaccine manufacturing (only surpassed by the cost for mRNA capping) and is a main cost driver of the production of mRNA vaccines.^[^ ^16^, ^17^ ^]^ The current study focuses on the challenge of synthetic supply of ΨTP (**1c**) and in particular, m^1^ΨTP (**3c**).

**Figure 1:**
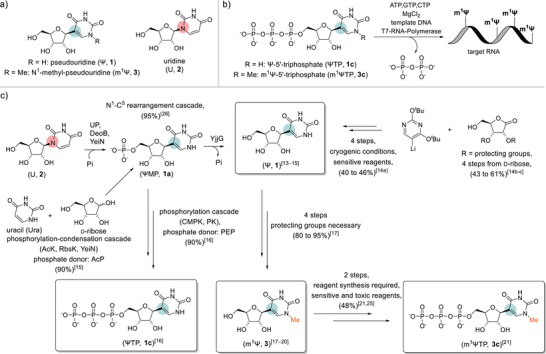
a) Structural comparison of *C*‐nucleosides Ψ (**1**) and m^1^Ψ (**3**) and *N*‐nucleoside U (**2**). b) General scheme of in vitro transcription used for mRNA vaccine production. c) Selected examples of existing biocatalytic and chemical routes of (m^1^)ΨTP synthesis. AcP, acetyl phosphate; PEP, 2‐phosphoenolpyruvate; Pi, inorganic phosphate; Rib, D‐ribose; Ura, uracil. Enzymes: AcK, acetate kinase (EC 2.7.2.1); CMPK, cytidine 5′‐phosphate kinase (EC 2.7.4.25); DeoB, phosphopentomutase (EC 5.7.2.7); PK, pyruvate kinase (EC 2.7.1.40); UP, uridine phosphorylase (EC 2.4.2.3); RbsK, ribokinase (EC 2.7.1.15); YeiN, ΨMP *C*‐glycosidase (EC 4.2.1.70); YjjG, pyrimidine 5′‐nucleotidase (EC 3.1.3.5).

Reported chemical routes to m^1^ΨTP (**3c**) are based on a common strategy in three main steps: synthesis of Ψ,^[^ ^18^, ^19^, ^20^, ^21^, ^22^, ^23^, ^24^, ^25^ ^]^ *N* ^1^‐methylation of Ψ,1^[^ ^26^, ^27^, ^28^ ^]^ and iterative phosphorylation^[^ ^29^, ^30^ ^]^ of m^1^Ψ. Robust synthetic methods are available for each step (Figure 1c), yet there remains the important need for improvements in efficiency and sustainability of the transformations used. Biocatalysis has shown disruptive potential in the development of a production‐amenable process chemistry for the synthesis of Ψ (**1**), or the 5′‐phosphate thereof (ΨMP, **1a**), from readily accessible substrates (Figure 1c).^[^ ^31^, ^32^, ^33^, ^34^, ^35^ ^]^ Site‐ and stereoselective coupling between the ribose 5‐phosphate (Rib5P) *C*‐1 and the uracil (Ura) *C*‐5 catalyzed by ΨMP *C*‐glycosidase is the key step to install the β‐*C*‐ribosyl residue on the Ura nucleobase.^[^ ^36^, ^37^ ^]^ ΨMP (**1a**) is produced very efficiently by a multistep one‐pot rearrangement of U (**2**) or directly from Rib5P and Ura (Figure 1c).^[^ ^31^, ^32^, ^35^ ^]^ ΨTP (**1c**) can then be obtained by enzymatic phosphorylation of ΨMP (**1a**) with ATP (Figure 1c).^[^ ^32^, ^35^ ^]^ For *N* ^1^‐methylation of Ψ, however, no biocatalytic alternative is known. A relevant methyltransferase has not been identified yet despite the fact that Ψ (**1**) sites in RNA can become *N* ^1^‐methylated post‐transcriptionally.^[^ ^38^, ^39^ ^]^ Moreover, important advances in methyltransferase cascade transformations notwithstanding^[^ ^40^, ^41^, ^42^, ^43^, ^44^, ^45^, ^46^, ^47^, ^48^ ^]^ the choice of donor substrate for enzymatic methylation reactions remain an unsolved issue in general.

Here, we present an integrated chemoenzymatic approach of m^1^ΨTP synthesis that combines chemical methylation of ΨMP (**1a**) with biocatalytic cascade transformations for the formation of ΨMP from U (**2**). Additionally, we show the two‐step iterative phosphorylation of m^1^ΨMP (**3a**). Although precedents exist for the individual steps, the steps have not been realized on the relevant substrates and lack consolidation into an overall synthetic route. We pinpoint specific requirements of ΨMP (**1a**) for *N* ^1^‐methylation and identify kinases well‐suited from their activity for cascade phosphorylation of ΨMP (**1a**) and m^1^ΨMP (**3a**), using ATP supplied in situ from acetyl phosphate (AcP) as an expedient donor substrate (Scheme 1). We show m^1^ΨTP (**3c**) production in up to 68% overall yield from U (**2**) at a concentration of ∼50 mg mL^−1^ and a scale of ∼200 mg of isolated product. Significant improvements in process performance metrics are highlighted by comparing the new chemoenzymatic synthesis to an established chemical procedure starting from Ψ (**1**).

**Scheme 1:**
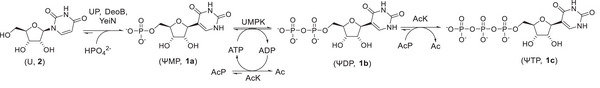
Proposed efficiency‐enhanced enzymatic one‐pot phosphorylation cascade from ΨMP (**1a**) to ΨTP (**1c**) via ΨDP (**1b**). The reactions of UP, DeoB and UMPK involve an equilibrium more balanced between substrate and product. The YeiN and AcK reactions are quasi‐irreversible due to equilibria strongly favoring ΨMP (**1a**) and ΨTP (**1c**) synthesis, respectively. ^[16,40]^ AcP was used as the overall phosphate‐donor. Ac, acetate; UMPK, uridine 5′‐phosphate kinase (EC 2.7.4.14).

We began our investigation by using the three‐enzyme cascade rearrangement^[^ ^31^ ^]^ shown in Figure 1c. ΨMP (**1a**) was obtained in excellent yield (95%) from U (**2**) (∼1 mol L^−1^) and a scale of up to ∼1.6 g product was demonstrated (Figure S1). Merely filtering off the enzymes was sufficient to isolate ΨMP in a form and purity suitable for its transformation into ΨTP (**1c**). Iterative phosphorylation of ΨMP (**1a**) was shown in our earlier work by a cascade reaction of cytidine 5′‐phosphate kinase (CMPK, from *E. coli*) and pyruvate kinase (PK, from rabbit muscle).^[^ ^32^ ^]^ As shown in Figure S2, CMPK catalyzed the phosphorylation of ΨMP (**1a**) and the resulting ΨDP (**1b**) was phosphorylated by PK. ATP was used as phosphate donor and was regenerated from phosphoenolpyruvate (PEP) by PK. Low enzyme activity with ΨMP and ΨDP (∼0.1 units mg^−1^ CMPK, 1.5 units mg^−1^ PK, Table S1) together with the requirement for the expensive PEP rendered the cascade phosphorylation by CMPK and PK rather unattractive for preparative synthesis of ΨTP (**1c**) (Figure S2). Most importantly, however, the CMPK showed no activity with m^1^ΨMP (**3a**) (preparation of m^1^ΨMP is described later in full detail).

We therefore searched for an alternative kinase. We noticed the rather relaxed nucleoside 5′‐monophosphate specificity of UMP kinases (UMPK), curiously, a trait only observed in eukaryotic UMPKs.^[^ ^49^, ^50^, ^51^ ^]^ Structure comparison (Figure S3) revealed a less constrained binding pocket for the nucleobase in yeast and slime mold UMPK compared to *E. coli* UMPK. We were pleased to find excellent activity of *Saccharomyces cerevisiae* UMPK with ΨMP (∼100 units mg^−1^, Table S1) and retention of useable activity with the more challenging substrate m^1^ΨMP (**3a**, 0.3 units mg^−1^, Table S1). Notably, except for a kinase in a recent patent (i.e., PPK2) that was used without further characterization,^[^ ^52^ ^]^ no kinase was previously reported for the reaction with m^1^ΨMP (**3a**). Acetate kinase (AcK) is well‐known for ATP regeneration from AcP.^[^ ^46^, ^53^, ^54^, ^55^, ^56^ ^]^ In recent work,^[^ ^35^ ^]^ we showed AcP‐driven ATP‐dependent phosphorylation of D‐ribose (Rib) for the enzymatic production of ΨMP in the presence of Ura. Studying the AcK from *E. coli*, we here discovered that the enzyme exhibits promiscuous activity for phosphorylation of both ΨDP (**1b**; 40 units mg^−1^) and m^1^ΨDP (**3b**; 200 units mg^−1^, Table S1). One‐pot cascade phosphorylation of ΨMP (**1a**; Scheme 1) was therefore done using UMPK and AcK. After thorough optimization of the reaction conditions (Figures S4–S6) and scale up, the cascade reaction now allowed for a highly efficient production of ΨTP (**1c**) as a single product of the transformation (Figure 2a). It will be noted that in the iterative phosphorylation of ΨMP (**1a**), distribution of the substrate between ΨDP (**1b**) and ΨTP (**1c**) products could become problematic for the overall efficiency of the synthesis. The clean conversion of ΨMP (**1a**) into ΨTP (**1c**, ≥95% yield) effectively without ΨDP (**1b**) accumulating (≤3%) was therefore remarkable. Nonetheless, Figure S5 shows that without suitable optimization, a mixture of ΨDP (**1b**) and ΨTP (**1c**) was obtained from which isolation of the ΨTP (**1c**) would have been cumbersome.

**Figure 2:**
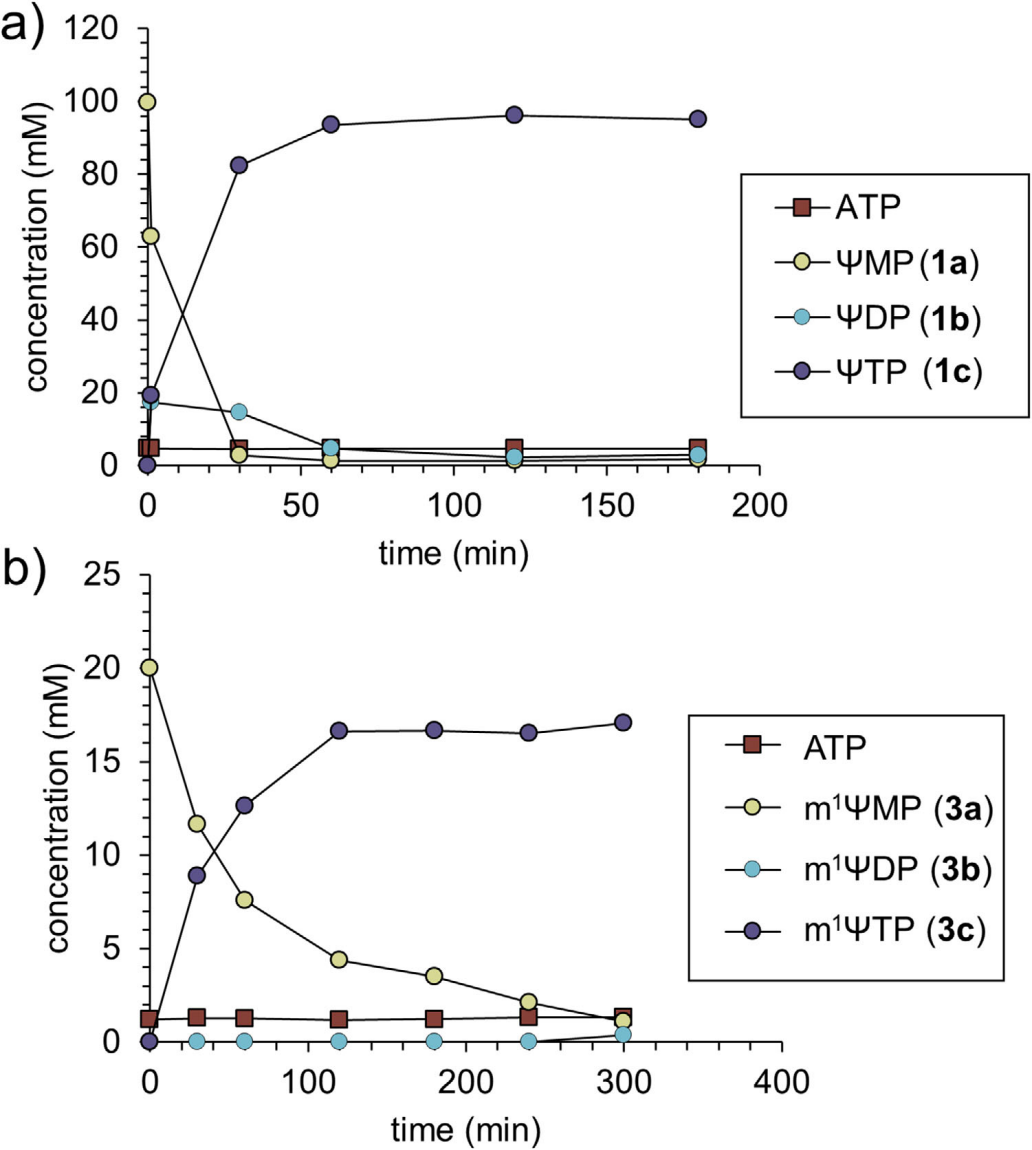
Time course analysis of phosphorylation. a) Reaction time course of ΨTP (**1c**) synthesis. Here, 50 mM TAPS buffer (pH 8.0–8.5), 100 mM ΨMP (**1a**), 5.0 mM ATP, 300 mM AcP, 10 mM MgCl_2_, 0.1 mg mL^−1^ UMPK and AcK were incubated at 30 °C (*n* = 1 individual experiment). The reaction was performed at 5.8 mL scale. b) Reaction time course of m^1^ΨTP (**3c**) synthesis. Here, 50 mM TAPS buffer (pH 8.5), 20 mM m^1^ΨMP (**3a**), 1.25 mM ATP, 75 mM AcP, 10 mM MgCl_2_, 0.7 mg mL^−1^ UMPK and 0.7 mg mL^−1^ AcK were incubated at 30 °C. The reaction was performed at 17 mL scale.

Notably, compared to the previously reported phosphorylation with CMPK and PK,^[^ ^32^ ^]^ the ΨTP (**3c**) concentration (∼50 mg mL^−1^) could be increased 10‐fold and the productivity (71 mg mL^−1^ h^−1^) could be enhanced eight‐fold, despite the fact that the enzyme use was lowered by 50‐ and 2‐fold for the monophosphate and diphosphate kinase, respectively. ΨTP (**3c**) was eventually isolated in high purity (≥97%; HPLC) and yield (85%; 271 mg) from the ΨMP (**3a**) used (Figure S7). The expected product structure was confirmed by NMR (Figures S21–S23).

Having found suitable conditions for the cascade phosphorylation, we next turned to the synthesis of m^1^ΨMP (**3a**) from ΨMP (**1a**), as summarized in Scheme 2a. Considering existing protocols for *N* ^1^‐methylation of the non‐phosphorylated Ψ (**1**),^[^ ^26^, ^27^, ^28^ ^]^ we examined a reaction of the acetonide‐protected ΨMP (**4**) [obtained in near‐quantitative yield from ΨMP (**1a**)] under similar conditions (see the Supporting Information for details). Due to low solubility of ΨMP (**1a**), a large excess (20 eq.) of *N*,*O*‐bis(trimethylsilyl)acetamide (BSA) was applied, and also, more CH_3_I (10 eq.) was required to induce any conversion. However, we were surprised to find that instead of the expected *N‐*alkylation the substrate had undergone complete dephosphorylation with substitution to the corresponding 5′‐iodo compound. We therefore tested dimethyl sulfate (DMS) as an alternative and even less expensive methylation reagent. To our delight, DMS smoothly facilitated the selective transformation into the *N* ^1^‐methylated intermediate after stirring under reflux for 4 d. Following optimization, m^1^ΨMP (**3a**) could be obtained in very good yield (85%) after deprotection, deprotonation and reversed‐phase column chromatography (Scheme 2a). The correct structure of m^1^ΨMP (**3a**, including the critical position of the methyl group) was confirmed by 2D‐NMR experiments (Figures S33 and S34). It should be noted that only with a large excess of BSA used, the undesired *N* ^3^‐methylation was suppressed fully.

**Scheme 2:**
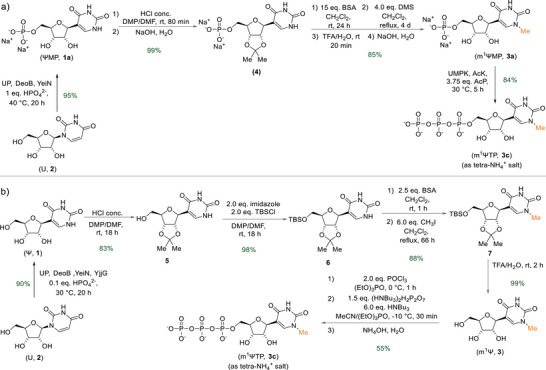
Synthetic routes analyzed. a) Acetonide protection, direct *N^1^*‐selective methylation and one‐pot enzymatic phosphorylation of ΨMP (**1a**). b) Comparative chemical synthesis of m^1^ΨTP (**3c**) starting from Ψ (**1**). BSA, *N,O*‐bis(trimethylsilyl)acetamide; DMF, *N,N*‐dimethylformamide; DMP, 2,2‐dimethoxypropane; DMS, dimethyl sulfate; TBS, *tert*‐butyldimethylsilyl; TFA, trifluoroacetic acid.

Next, we turned our focus to the iterative phosphorylation of m^1^ΨMP (**3a**) for its conversion into m^1^ΨTP (**3c**), as shown in Scheme 1. The activity of *S. cerevisiae* UMPK was significantly reduced with m^1^ΨMP (**3a**) compared to ΨMP (**1a**, Table S1, Figure S8b), likely due to steric interference caused by the *N* ^1^‐methyl group in the enzyme's binding pocket (Figure S9), resulting in a slower progression of the reaction.

It was therefore convenient to raise the UMPK concentration (0.7 mg mL^−1^; 10‐fold) and decrease the m^1^ΨMP (**3a**) substrate concentration to 20 mmol L^−1^ (5‐fold). With these adaptations in place, m^1^ΨTP (**3c**) was synthesized as a single product in this transformation from m^1^ΨMP (**3a**) in high yield (83%) and at high productivity (6.5 g L^−1^ h^−1^_,_ based on free acid; Figure S8c). Scale up of the reaction to 17 mL proceeded smoothly without notable changes in performance (Figure 2a), furnishing m^1^ΨTP (**3c**) in high yield (84%, 157 mg, tetra‐NH_4_ salt) and purity (98%) following product isolation (Figure S10) analogously as shown for ΨTP (**1c**). The expected structure was verified by NMR (Figures S24–S26). To introduce a relevant benchmark for the chemoenzymatic synthesis of m^1^ΨTP (**3c**) shown here, we additionally prepared m^1^ΨTP (**3c**) by purely chemical means starting from Ψ (**1**) (Scheme 2b). The Ψ (**1**) was obtained by biocatalytic cascade rearrangement of U (**2**), reported in our previous study (Figure S11).^[^ ^31^ ^]^ To ensure selective *N*‐methylation, a 2′,3′‐*O*‐isopropylidene protecting group (**5**) as well as a 5′‐*O*‐(*tert*‐butyldimethylsilyl) (TBS) protecting group (**6**) was introduced. *N* ^1^‐selective methylation was then achieved using BSA/MeI,^[^ ^26^, ^57^ ^]^ delivering **7** in very good yield. NMR experiments again confirmed the correct *N*‐methylation site (Figures S41 and S42). After complete deprotection of **7** with a TFA/H_2_O mixture, phosphorylation of the intermediary m^1^Ψ (**3**) according to reported protocols^[^ ^29^, ^30^ ^]^ eventually furnished m^1^ΨTP (**3c**) in acceptable yield (55%, 299 mg, tetra‐NH_4_ salt) after isolation by ion‐exchange chromatography.

Having now all desired nucleotide building blocks in hand, we then tested them in experiments of IVT. The IVT served as a critical test of the usability for the synthetic preparations of m^1^ΨTP (**3c**) and ΨTP (**1c**, Figure 3). The reactions were analyzed under conditions of uniform U‐to‐(m^1^)Ψ exchange where m^1^ΨTP (**3c**) or ΨTP (**1c**) replaced the UTP of the positive control. The DNA template migrated to a position of ∼1000 bp size in the agarose gel (Figure 3), consistent with expected size of the linearized DNA template (1160 bp, Figure S50). The single‐stranded RNA product migrated to a position of ∼500 bp (Figure 3). The apparent size of the RNA smaller significantly than the DNA template was probably caused by the formation of secondary structural elements. Both m^1^ΨTP (**3c**) preparations were similarly efficient as UTP and only a single product of consistent size was obtained in the reactions (Figure 3). ΨTP (**1c**) behaved analogously. These results provide a first validation of the *C*‐nucleotide triphosphate samples as monomer building blocks for the preparation of synthetic mRNA.

**Figure 3:**
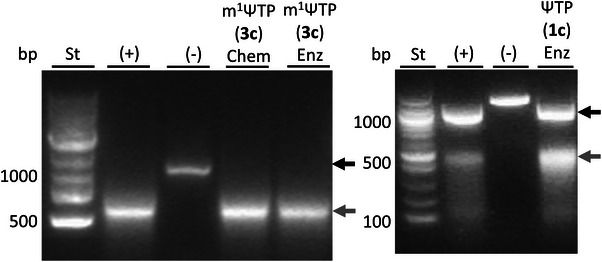
Analytical agarose gel of products from IVT. Reactions were done with 2.0 mM of each ATP, CTP, GTP, UTP or UTP‐replacing *C*‐nucleotide, 1.0 µg linear DNA template (YeiN‐encoding gene, Figure S50) and 1.2 units µL^−1^ RNA polymerase, incubated for 2 h at 37 °C. The positive control (+) used UTP, the negative control (‐) lacked UTP. Other reactions replaced UTP with ΨTP (**1c**) or m^1^ΨTP (**3c**) synthesized chemoenzymatically (lane: Enz) or chemically (lane: Chem). The bands of DNA template (black) and RNA product (grey) are indicated by arrows. In the left panel, the DNA template is not visible for unknown reason. St, base pair (bp) standard.

Finally, we evaluated the synthetic routes to m^1^ΨTP (**3c**) and ΨTP (**1c**) based on key benchmark metrics of process performance.^[^ ^58^, ^59^, ^60^, ^61^ ^]^ Results are summarized in Table 1. A breakdown of each route according to the individual reaction steps comprised is given in Tables S2 and S3 (the full calculation can be found in the Supporting Table “E factor calculations”). Not included is however the production of the enzymes and the product isolation from the reaction mixture. At this point, the chosen isolation via ion exchange chromatography was not optimized and thus resulted in a huge step E factor of ∼9000 (sE factor = 86) due to the high volume of mobile phase used. We envision that at larger scale, the triphosphates might be precipitated from the reaction mixture (e.g., by ethanol co‐solvent),^[^ ^62^, ^63^ ^]^ but did not consider this in the current study. The synthesis of ΨTP (**1c**) excels by the low number of reaction steps and the high overall yield. The main contribution to the overall E factor (E = 53) comes from the auxiliary step of chemical preparation of AcP for phosphorylation (E  =  35). By excluding all recyclable solvents, the sE factor even decreases further to a value as low as 3.8. The immense power of biocatalysis to improve process sustainability metrics is highlighted by comparing the E factors of enzymatic [E  =  15 for ΨMP (**1a**), E  =  77 for m^1^ΨMP(**3a**)] and chemical phosphorylation (E = 133, starting from m^1^Ψ (**3**), Tables S2, S3). Furthermore, no hazardous reagent is required in the enzymatic phosphorylation whereas POCl_3_ is used in the chemical reaction. Moreover, the enzymatic phosphorylation of m^1^ΨMP (**3a**) outperforms the chemical phosphorylation also in terms of yield (∼85% vs. ∼55%).

**Table 1:** Selected process parameters for *C*‐nucleotide syntheses starting from U (**2**).

Product	No. of steps	Yield^a)^ (%)	Scale (mg)	E factor^b)^	sE factor^c)^	Solvents	Hazardous reagents
ΨTP (**1c**)	3	83	271	53	3.8	EtOAc	none
m^1^ΨTP (**3c**)^d)^	4	68	157	2512	31	DMF, DCM	DMS
m^1^ΨTP (**3c**)^e)^	6	37	299	5617	26	DMF, DCM	CH_3_I, POCl_3_

^a)^ Overall isolated yield based on U (**2**).

^b)^ E factor $=\frac{\mathrm{mas}s_{\mathrm{prod}\mathrm{uct}}\left(g\right)}{\mathrm{mas}s_{\mathrm{waste}}\left(g\right)}$.

^c)^ sE Factor, E factor excluding the solvents used from the calculation.

^d)^ Synthesized via chemoenzymatic route (U→ΨMP→m^1^ΨMP→m^1^ΨTP, see Scheme 2a).

^e)^ Synthesized via chemical route (U→Ψ→m^1^Ψ→m^1^ΨTP, see Scheme 2b).

As a major drawback, yet highly convenient transformation, the chemical methylation contributes almost all (∼95%) of the total E factor of 2.5 × 10^3^ of the chemoenzymatic synthesis of m^1^ΨTP (**3c**). It employs a hazardous reagent in excess and the solvents used, in particular DCM, are nonpreferred from the green chemistry point of view.^[^ ^64^, ^65^ ^]^ Solvent recycling could however decrease the E factor substantially to a value of ∼31 (Table 1). The search of safer solvents for the chemical methylation should receive attention in future studies. When compared to ΨTP (**1c**), the synthesis of m^1^ΨTP (**3c**) requires an additional step and the overall yield is thus reduced to 68%. Hence, addressing the *N* ^1^‐methylation of ΨMP (**1a**) by means of biocatalysis promises significant improvement in the sustainability metrics of the m^1^ΨTP (**3c**) synthetic route. As a positive side effect, a reaction in water not only addresses the concern of solvent use, but it might also benefit the transformation efficiency due to improved solubility of the ΨMP (**1a**) substrate. Much progress has been made lately in the development of methyltransferases for biocatalytic methylation in organic synthesis.^[^ ^40^, ^41^, ^42^, ^43^, ^44^, ^46^ ^]^ The site‐selective *N*‐methylation of nucleobase moieties in non‐RNA context has, however, not been shown by a methyltransferase reaction to our knowledge. The enzymatic methyl donor substrate (*S*‐adenosylmethionine) can be regenerated through enzymatic cascade reactions either by complex biomimetic systems,^[^ ^45^, ^66^, ^67^ ^]^ or from standard chemical reagents of methylation (e.g., CH_3_I).^[^ ^42^ ^]^ However, CH_3_I is hardly soluble, and also unstable, in water. Enzymatic methylation reactions for synthesis often involve methyl donor used in an excess over substrate not different from the corresponding chemical methylation.^[^ ^42^ ^]^ Therefore, at this stage, substitution of chemical by enzymatic methylation is highly debatable and we, consequently, left this interesting point for m^1^ΨTP (**3c**) synthesis for consideration in the future. Overall, our early route assessment based on comparable data for the synthetic reactions demonstrates the substantial leverage generated by biocatalysis to enhance the process performance metrics for efficiency and sustainability of production of m^1^ΨTP (**3c**) and ΨTP (**1c**). Our analysis excluded the critical C─C coupling to generate the Ψ (**1**) core structure by purely chemical means because the advantages of the biocatalytic rearrangement are compelling. In addition to the sustainability analysis presented here, the results can provide a useful basis for techno‐economic evaluation.

In summary, we demonstrate an integrated chemoenzymatic route for the synthesis of m^1^ΨTP. Rigorous benchmarking of this new route against state‐of‐the‐art purely chemical synthesis of m^1^ΨTP highlights benefits in terms of efficiency and sustainability. The research findings contribute to the broader objective of incorporating applied biocatalysis into the industrial synthesis of advanced pharmaceutical ingredients.^[^ ^68^, ^69^, ^70^, ^71^ ^]^ Ultimately, we hope that the improved synthesis of m^1^ΨTP can contribute to a lowering of the production cost of mRNA therapeutics in general and making these powerful new modalities better accessible to benefit more people.^[^ ^16^, ^17^ ^]^

## Supporting Information

Supplementary Figures and Tables as well as experimental details are shown in the Supporting Information. A supplementary table provides details of E factor calculation. Additional references are cited in the Supporting Information.^[^ ^72^, ^73^, ^74^, ^75^, ^76^ ^]^

## Conflict of Interests

The authors declare no conflict of interest.

## Supporting information



Supporting Information

Supporting Information

## Data Availability

The data that support the findings of this study are available from the corresponding author upon reasonable request.
