# Supplementary material for: Integrated Chemoenzymatic Synthesis of the mRNA Vaccine Building Block N 1‐Methylpseudouridine Triphosphate
Source: Angew Chem Int Ed Engl. 2025 Jul 4;64(34):e202506330. doi: 10.1002/anie.202506330 (PMC12363647; doi:10.1002/anie.202506330)
Supplement: Supplementary file 1 — Supporting Information [file ANIE-64-e202506330-s001.pdf]

Supporting Information

Integrated Chemoenzymatic Synthesis of the mRNA Vaccine Building Block N^1-Methylpseudouridine Triphosphate

Martin Pfeiffer,[a] # Leo Krammer,[b] # Johannes Zöhrer,[a] Rolf Breinbauer,[b,d]* and Bernd Nidetzky[a,c]*

[a] Dr. M. Pfeiffer, J. Zöhrer, Prof. B. Nidetzky
Institute of Biotechnology and Biochemical Engineering
Graz University of Technology
Petersgasse 12/1, 8010 Graz (Austria)
E-mail: bernd.nidetzky@tugraz.at

[b] Dr. L. Krammer, Prof. R. Breinbauer
Institute of Organic Chemistry
Graz University of Technology
Stremayrgasse 9/Z4, 8010 Graz (Austria)
E-mail: breinbauer@tugraz.at

[c] Prof. B. Nidetzky
Austrian Centre of Industrial Biotechnology (acib)
Krenngasse 37, 8010 Graz (Austria)

[d] Prof. R. Breinbauer
BioTechMed-Graz
8010 Graz (Austria)

[#] Co-first authors.

Table of Contents

Supporting Figures **4**

Supporting Tables **16**

Methods **19**

General aspects **19**

Analytical methods **19**

High performance liquid chromatography with mass spectrometry 19

Nuclear magnetic resonance spectroscopy 20

High resolution mass spectrometry 20

Determination of melting points 21

Thin layer chromatography 21

Normal phase flash column chromatography 21

Reversed phase flash column chromatography 22

Determination of the concentration of nucleosides, nucleotides and enzymes 22

Experimental procedures **23**

Structure overview 23

Enzyme production 23

Enzyme assays 24

CMPK-PK cascade phosphorylation 26

UMPK-AcK cascade phosphorylation 26

Operational stability 26

Molecular docking of UDP, ΨDP (**1b**) and m^1ΨDP (**3b**) to UMPK. 27

Enzymatic synthesis of Ψ (**1**) and ΨMP (**1a**). 28

Enzymatic synthesis of ΨTP (**1c**). 29

General procedure for the 2',3'-*O*-isopropylidene protection 30

Synthesis of 2',3'-*O*-isopropylidene-ΨMP (**4**) 31

Synthesis of 2',3'-*O*-isopropylidene-Ψ (**5**) 32

Synthesis of m^1ΨMP (**3a**) 33

Synthesis of 2',3'-*O*-isopropylidene-5'-*O*-(*tert*-butyldimethylsilyl)-Ψ (**6**) 34

Synthesis of N^1-methyl-2',3'-*O*-isopropylidene-5'-*O*-(*tert*-butyldimethylsilyl)-Ψ (**7**) 35

Synthesis of m^1Ψ (**3**) 36

Synthesis of m^1ΨTP (**3c**) 37

Biocatalytic synthesis of m^1ΨTP (**3c**) 38

Synthesis of acetyl phosphate 38

In vitro transcription.....39
Calculation of E factor.....40
NMR appendix.....41
IVT appendix.....76
References.....79

Supporting Figures

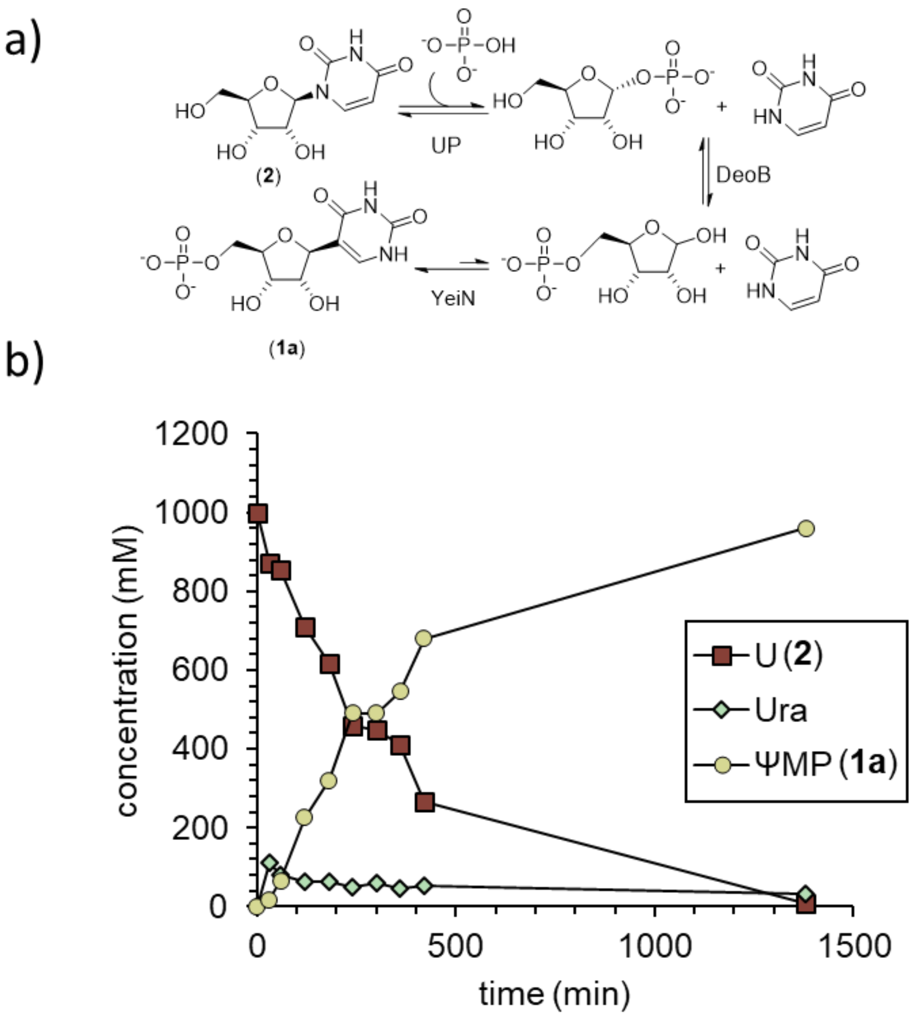

Figure S1. Synthesis of ΨMP (**1a**). a) Scheme of the enzymatic cascade reaction. The YeiN reaction is shown as quasi-irreversible because the equilibrium is very far on the side of ΨMP (**1a**) production. The reactions of UP and DeoB involve equilibrium more balanced between substrate and product. b) Time course of ΨMP (**1a**) synthesis. The reaction mixture contained 1.0 M sodium phosphate buffer at pH 7.0, 1.0 M uridine (U, **2**), 20 mM $MnCl_2$, 0.25 mg mL^{-1} UP, 2.5 mg mL^{-1} DeoB, and 1.5 mg mL^{-1} YeiN and was incubated at 40 °C with 700 rpm agitation. The reaction was performed in 5.8 mL volume. The enzymes used are specified in Table S1.

a)

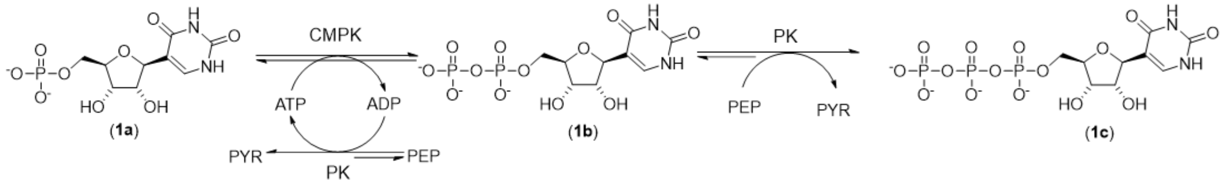

b)

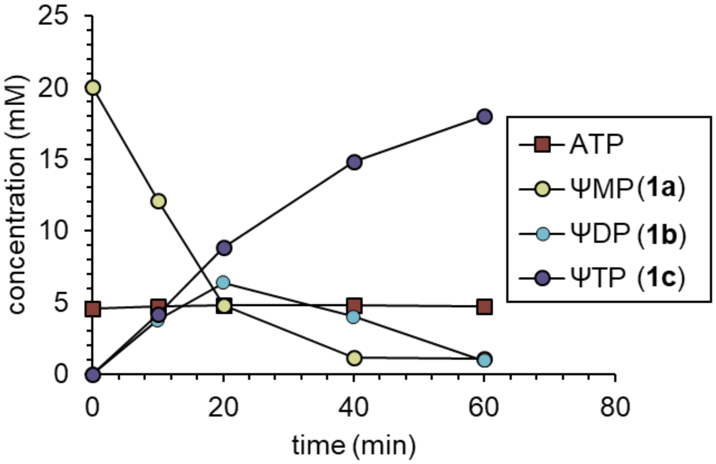

Figure S2. Synthesis of ΨTP (**1c**) using cascade reaction of PK and CMPK. a) Scheme of the enzymatic cascade reaction. The reactions of CMPK involves an equilibrium more balanced between substrate and product. The PK reaction is shown as quasi-irreversible because the equilibrium is very far on the side of ΨTP (1c). b) Time course of ΨTP (**1c**) synthesis. The reaction mixture contained 20 mM ΨMP (**1a**), 2.0 mM $MgCl_2$, 60 mM PEP, 5.0 mM ATP, 0.2 mg mL^{-1} PK, 3.0 mg mL^{-1} CMPK and was incubated at 30 °C with 350 rpm agitation. The reaction was performed in 200 µL volume. The enzymes used are specified in Table S1.

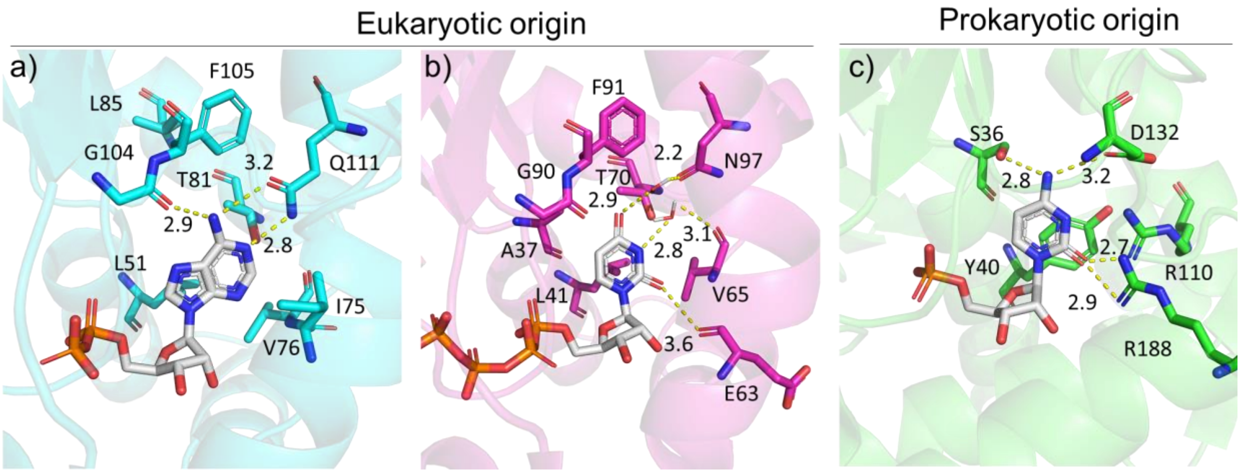

Figure S3. Nucleotide binding in different UMPK enzymes. Interaction distances are shown in Å. a) *Saccharomyces cerevisiae* UMPK bound with adenosine 5'-diphosphate, PDB identifier: 1UKY. b) *Dictyostelium discoideum* UMPK bound with P1-(adenosine-5'-P5-(uridine-5')pentaphosphate, PDB identifier: 1UKE. c) *Escherichia coli* UMPK bound with cytidine 5'-monophosphate, PDB identifier: 1KDO.

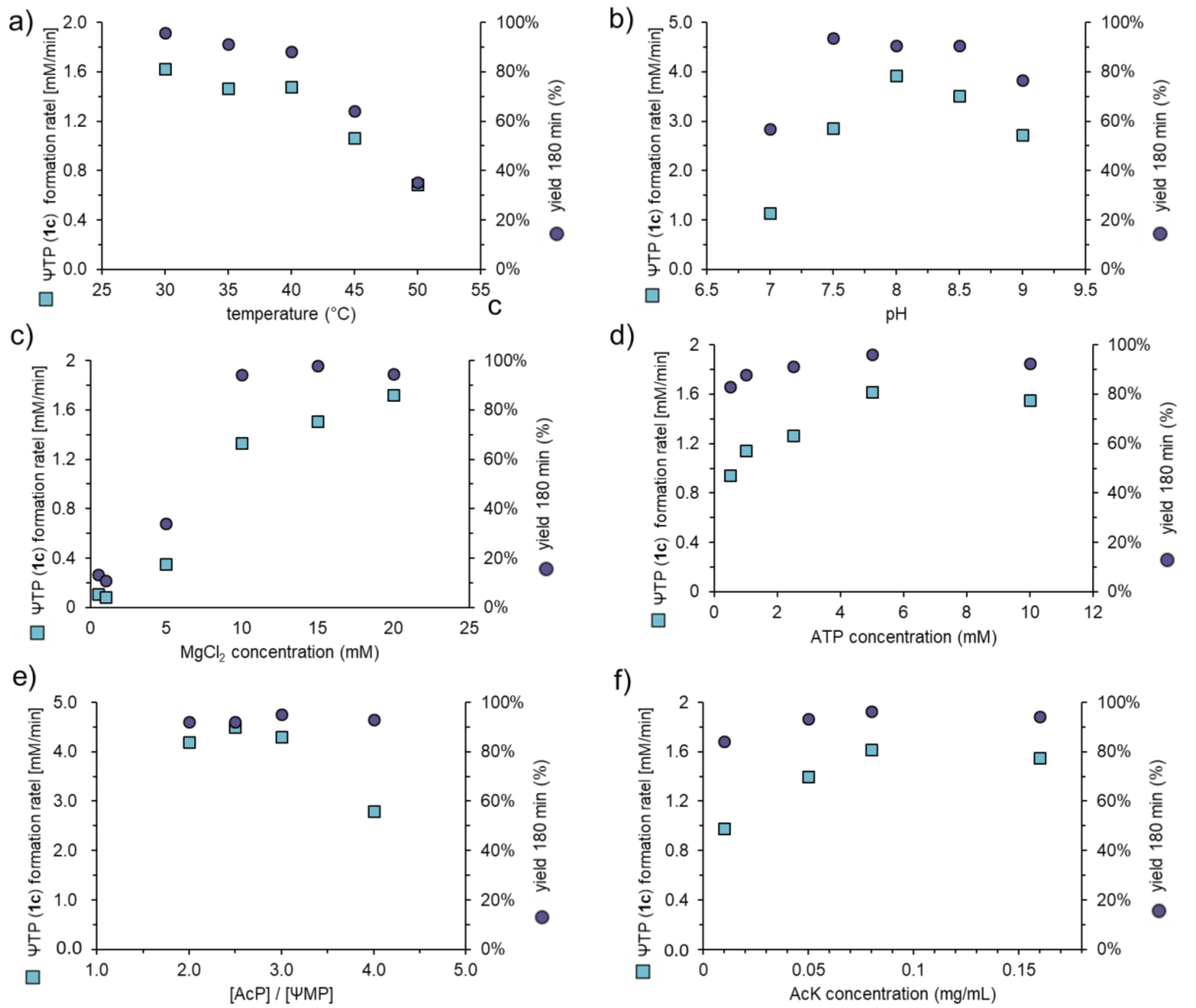

Figure S4. Analysis of reaction parameters and optimization of the UMPK-AcK cascade phosphorylation. Effect of (a) temperature, (b) pH, (c) $MgCl_2$ concentration, (d) ATP concentration, (e) AcP to ΨMP (**1a**) molar ratio and (f) AcK loading, on the ΨTP (**1c**) formation rate and the ΨTP (**1c**) yield after 180 min of reaction. Reactions were performed under standard condtions: 50 mM TAPS (pH 8.5), 100 mM ΨMP (**1a**), 5.0 mM ATP, 350 mM AcP, 10 mM $MgCl_2$, 0.08 mg mL^{-1} AcK and 0.03 mg mL^{-1} UMPK incubated at 30 °C (n=1) and 350 rpm agitation. All reactions were performed in 400 µL volume. Parameters that where optimized were varied in the range indicated in the panels. For further details, see the Methods section of this Supporting Information. The enzymes used are specified in Table S1.

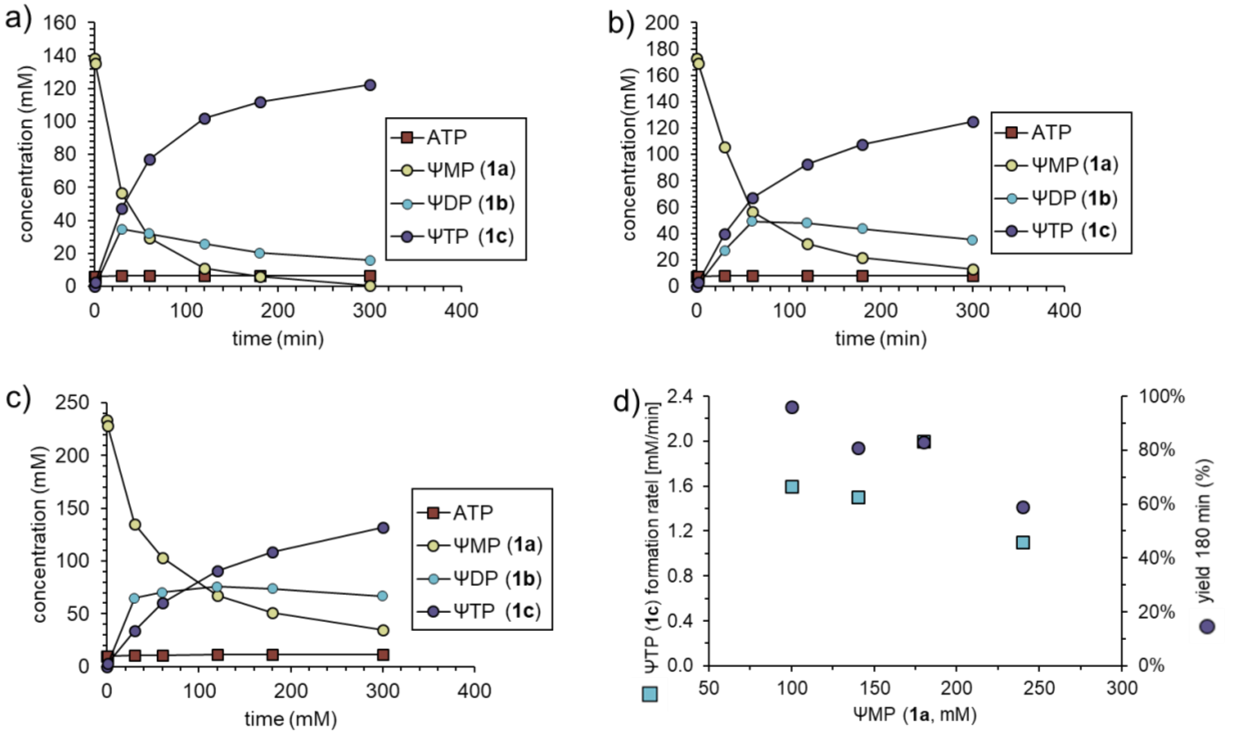

Figure S5. Reaction intensification for ΨTP (**1c**) synthesis. Reactions were performed in 50 mM TAPS, pH 8.5. a) 23 mM $MgCl_2$, 150 mM ΨMP (**1a**), 7.5 mM ATP, 375 mM AcP, 0.12 mg mL^{-1} AcK, and 0.45 mg mL^{-1} UMPK. b) 30 mM $MgCl_2$, 200 mM ΨMP (**1a**), 10 mM ATP, 500 mM AcP, 0.16 mg mL^{-1} AcK, and 0.6 mg mL^{-1} UMPK. c) 38 mM $MgCl_2$, 250 mM ΨMP (**1a**), 12.5 mM ATP, 625 mM AcP, 0.2 mg mL^{-1} AcK, and 0.75 mg mL^{-1} UMPK. d) Initial ΨTP (**1c**) formation rate and final reaction yield in dependence of the substrate concentration used. All reactions were done at 30 °C, 350 rpm agitation and used 400 µL volume. The enzymes used are specified in Table S1.

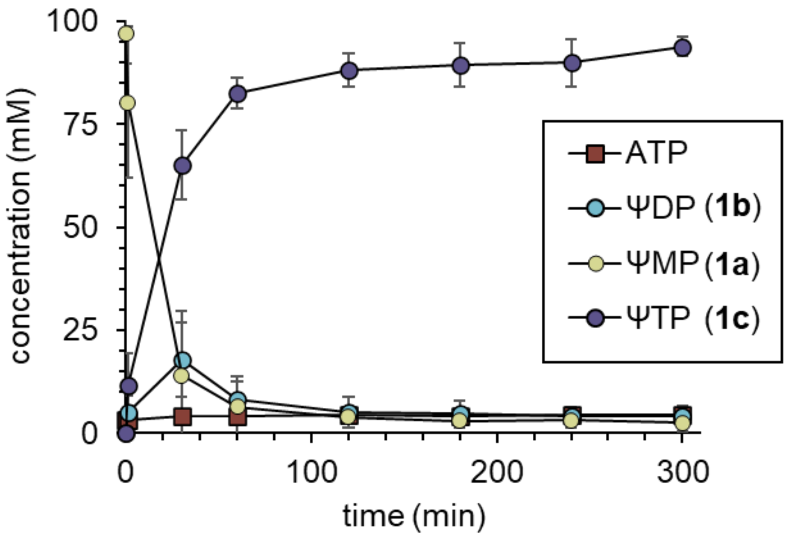

Figure S6. Reaction time course of the optimized ΨTP (**1c**) reaction. Here, 50 mM TAPS buffer (pH 8.0 to 8.5), 100 mM ΨMP, 5.0 mM ATP, 300 mM AcP, 10 mM $MgCl_2$, 0.1 mg mL^{-1} UMPK, and 0.1 mg mL^{-1} AcK were incubated at 30 °C. Error bars show S.D. (n = 3 individual experiments). The reactions were performed in 1.0 mL volume and 350 rpm agitation. The enzymes used are specified in Table S1.

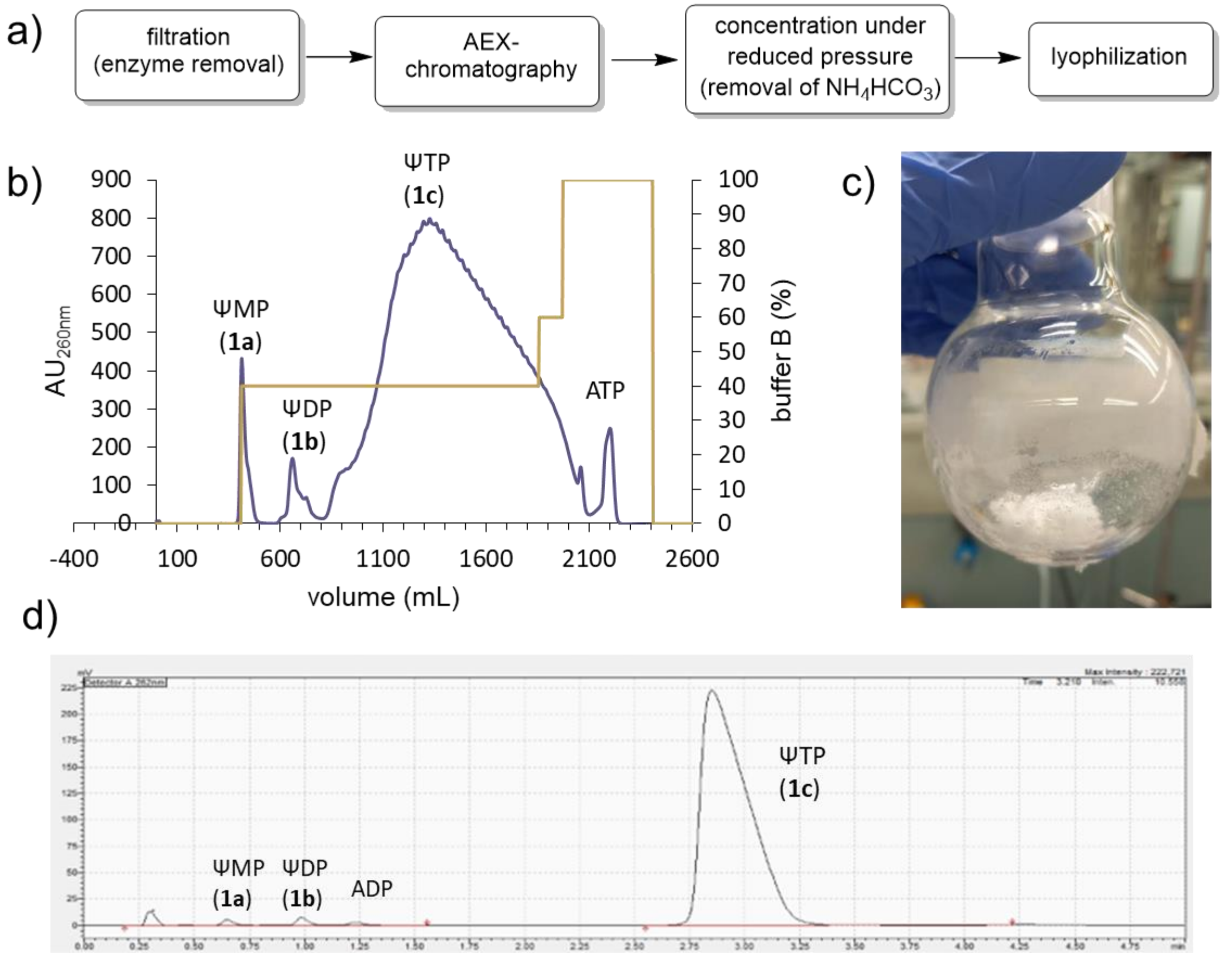

Figure S7. ΨTP (**1c**) isolation. a) General workflow of product isolation. AEX, anion exchange. b) Absorbance trace of the eluted volume in AEX chromatography, showing the isolation of ΨTP (**1c**). An isocratic concentration of 80 mM NH_4HCO_3 (50% buffer B) was used for elution. c) The tetra-ammonium salt of ΨTP (**1c**) forms a colorless hygroscopic powder after lyophilization. d) HPLC trace of the isolated product.

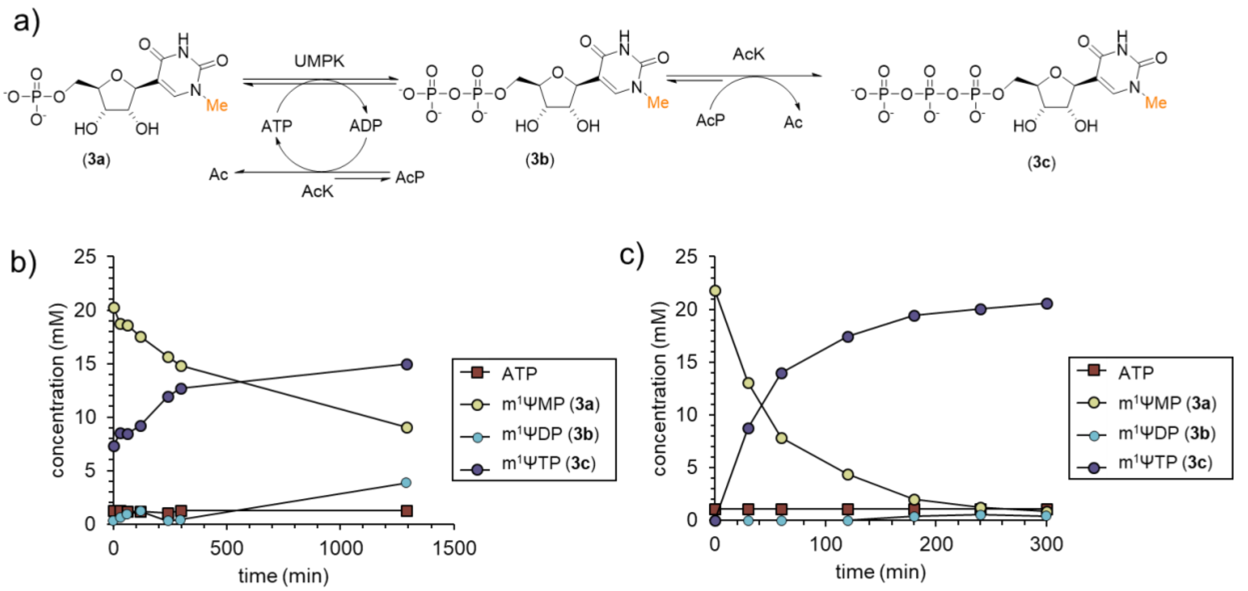

Figure S8. Synthesis of m^{1}ΨTP (**3c**) using cascade reaction of UMPK and AcK. a) Scheme of the enzymatic cascade reaction. The reactions of UMPK involves an equilibrium more balanced between substrate and product. The AcK reaction is shown as quasi-irreversible because the equilibrium is very far on the side of m^{1}ΨTP (**3c**). b) Reaction time course of unoptimized m^{1}ΨTP (**3c**) synthesis. Reaction conditions: 50 mM TAPS buffer (pH 8.5), 20 mM m^{1}ΨMP (**3a**), 1.25 mM ATP, 75 mM AcP, 10 mM $MgCl_2$, 0.2 mg mL^{-1} UMPK, and 0.2 mg mL^{-1} AcK; incubations were done at 30 °C 350 rpm agitation (n = 1 individual experiment). c) Reaction time course of the optimized m^{1}ΨTP (**3c**) synthesis. Reaction conditions: 50 mM TAPS buffer (pH 8.5), 20 mM m^{1}ΨMP (**3a**), 1.25 mM ATP, 75 mM AcP, 10 mM $MgCl_2$, 0.7 mg mL^{-1} UMPK, and 0.7 mg mL^{-1} AcK; incubations were done at 30 °C and 350 rpm agitation (n = 1 individual experiment). All reactions were performed in 400 µL volume. The enzymes used are specified in Table S1.

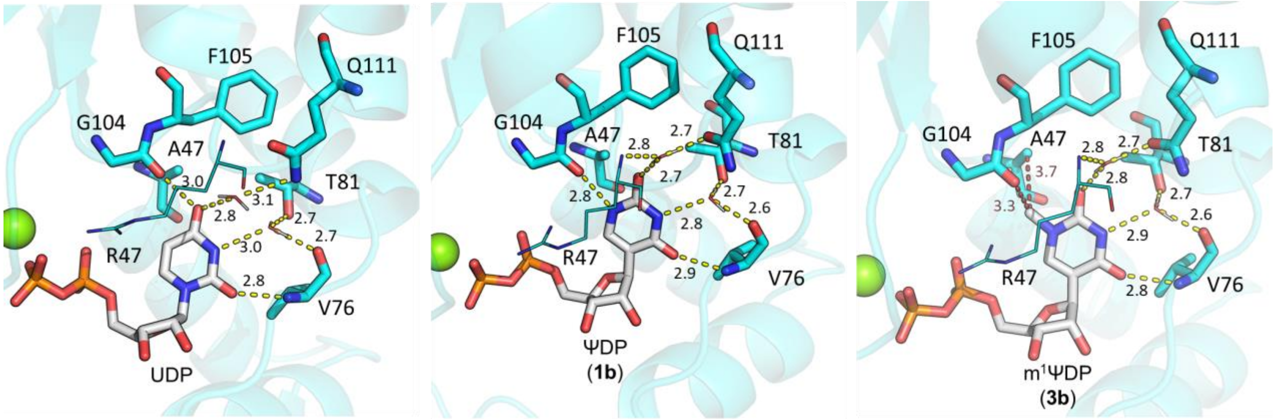

Figure S9. Interactions of UDP, ΨDP (**1b**) and m[1]ΨDP (**3b**) in *S. cerevisiae* UMPK. Distances are shown in Å. Polar interactions, yellow dashes; steric hindrance, red dashes. Enzyme-ligand complexes shown are the results of local dockings performed with Yasara. Experimental details are given in the Methods section.

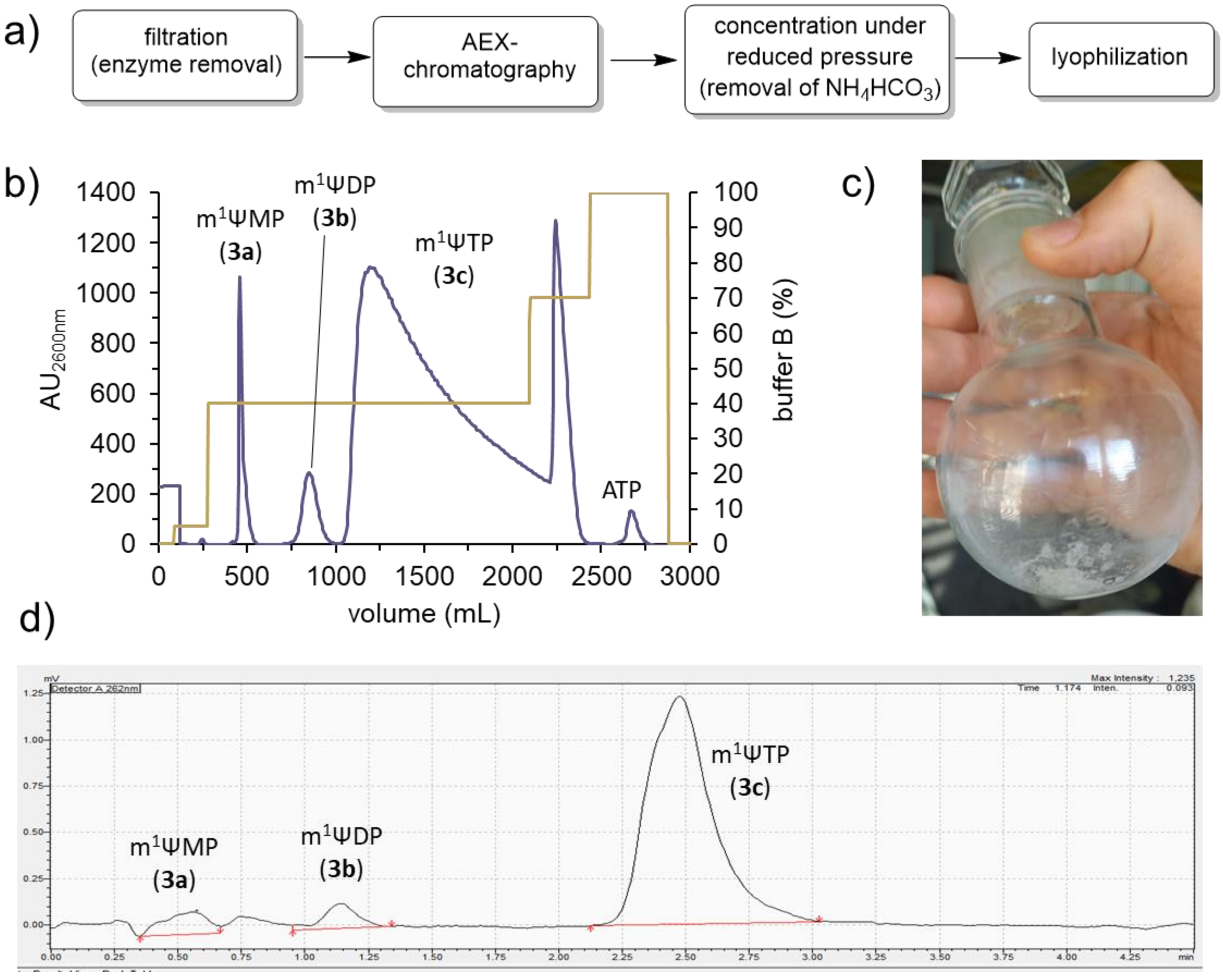

Figure S10. m[1]ΨTP (**3a**) isolation. a) General workflow of product isolation. AEX, anion exchange. b) Absorbance trace of the eluted volume in AEX chromatography, showing the isolation of m[1]ΨTP (**3a**). An isocratic concentration of 80 mM NH_4HCO_3 (50% buffer B) was used for elution. c) The tetra-ammonium salt of m[1]ΨTP (**3a**) forms a colorless hygroscopic powder after lyophilization. d) HPLC trace of the isolated product.

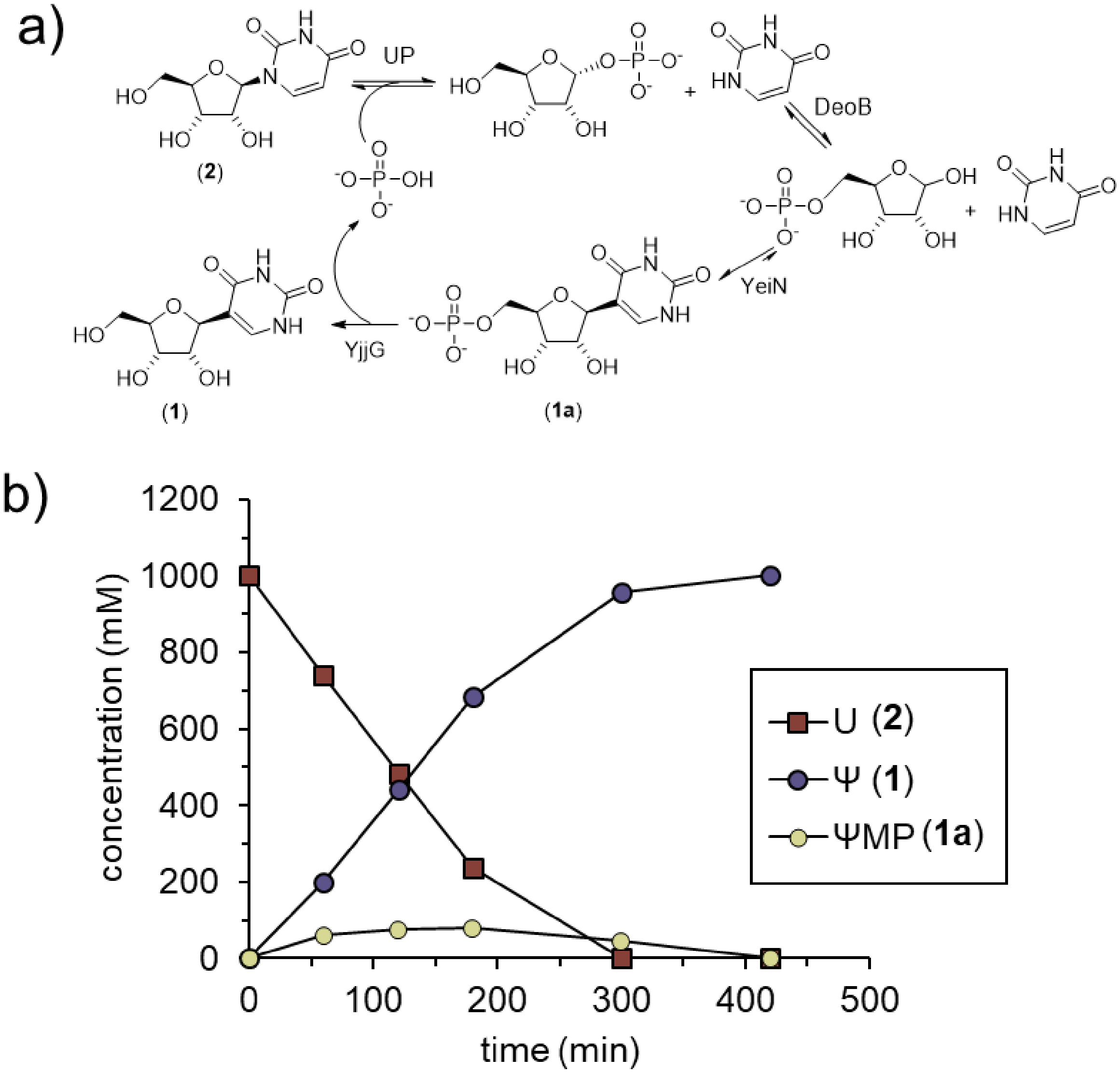

Figure S11. Synthesis of Ψ (**1**) by N-C cascade rearrangement of uridine (U, **2**).a) Scheme of the enzymatic cascade reaction. The reactions of UP and DeoB involves an equilibrium more balanced between substrate and product. The YeiN reaction is shown as quasi-irreversible because the equilibrium is very far on the side of ΨMP (**1a**). b) Reactions contained 0.1 M sodium phosphate buffer (pH 7.0), 1.0 M U (**2**), 2.0 mM $MnCl_2$, 0.3 mg mL^{-1} UP, 2.5 mg mL^{-1} DeoB, 1.5 mg mL^{-1} YeiN and 0.3 mg mL^{-1} YjjG. Incubation was performed at 30 °C and 700 rpm stirring. The reaction was performed in 5.0 mL volume. The enzymes used are specified in Table S1.

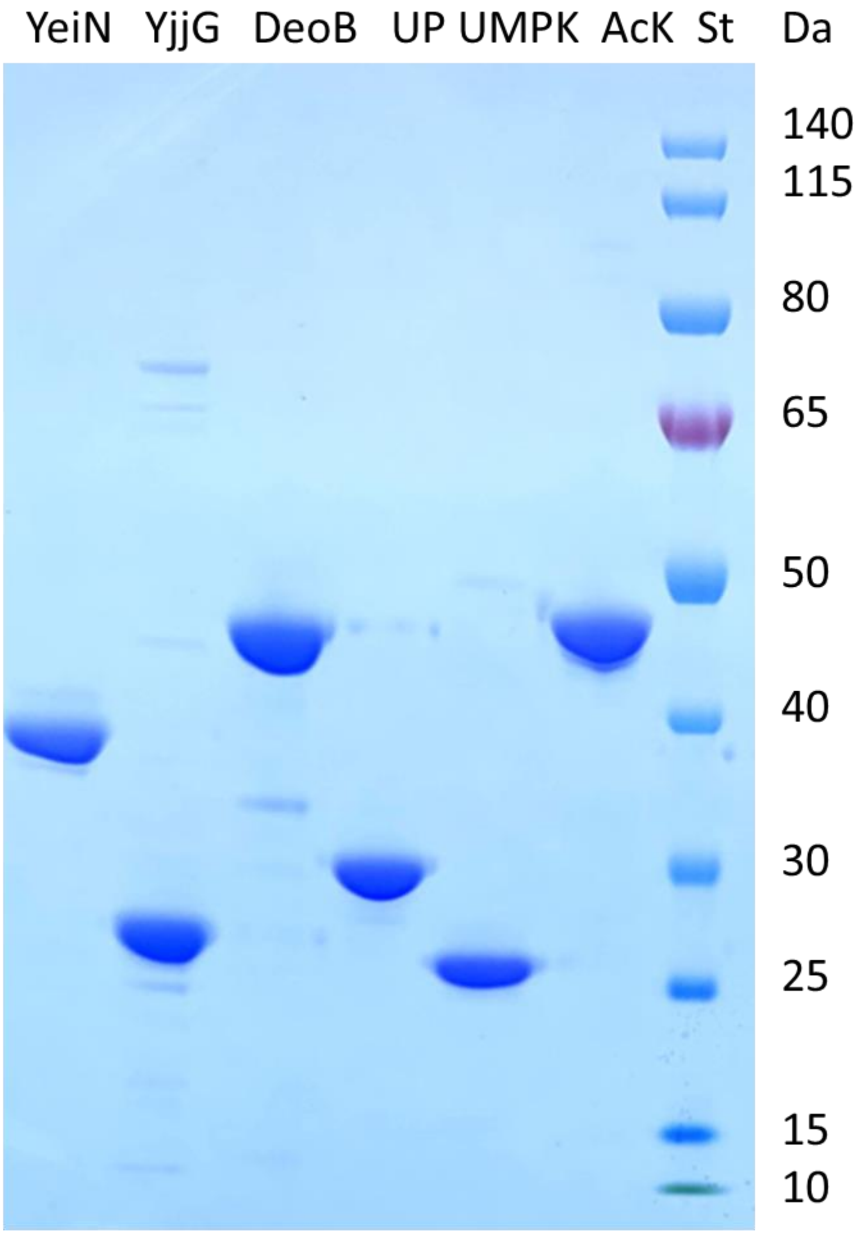

Figure S12. SDS polyacrylamide gel showing the purified enzyme preparations used in this study. The enzymes used are specified in Table S1.

Supporting Tables

Table S1. Summary of enzymes used and their properties.

**Enzyme**[a]	**Mol. mass (Da)**	**Tag**[c]	**Expression yield**[d] **(mg L^{-1})**	**Specific activity**[e] **(*U* mg^{-1})**	**Total turnover number**[f]
CMPK	26,610	Strep-tag II	50	0.1 (ΨMP)<br>n.d. (m^1ΨMP)<br>20 (UMP)	n.a.
PK[b]	58,048	*Not tagged*	n/a	1.5 (ΨDP)	n.a.
UMPK	45,453	His_6	190	99 (ΨMP)<br>197 (UMP)<br>0.3 (m^1ΨMP)	2.2×10^7
AcK	25,338	His_6	224	200 (m^1ΨDP)<br>40 (ΨDP)<br>2,290 (ADP)<br>741 (UDP)	3.6×10^6
YeiN	35,073	His_6	100	7.2 (Ura+Rib5P)	n.a.
UP	29,322	His_6	150	56 (U)	n.a.
DeoB	46,533	His_6	200	7.0 (Rib5P)	n.a.
YjjG	27,463	His_6	30	30 (UMP)	n.a.

[a] Definition of enzymes used: CMPK, cytidine 5'-phosphate kinase (EC 2.7.4.25), source: *Escherichia coli*, UniProt identifier: P0A6I0; PK, pyruvate kinase (EC 2.7.1.40), source: rabbit muscle, commercial preparation (CAS-Number 9001-59-6), from Sigma-Aldrich as ammonium sulfate suspension, UniProt identifier: P11974; UMPK, uridine 5'-phosphate kinase (EC 2.7.4.14), source: *Saccharomyces cerevisiae*, UniProt identifier: P15700; AcK, acetate kinase (EC 2.7.2.1), source *Escherichia coli*, UniProt identifier: P0A6A3; YeiN, ΨMP *C*-glycosidase (EC 4.2.1.70), source: *Escherichia coli*, UniProt identifier: P33025; DeoB, phosphopentomutase (EC 5.7.2.7), source: *Escherichia coli*, UniProt identifier: P0A6K6; YjjG, pyrimidine 5'-nucleotidase (EC 3.1.3.5), source: *Escherichia coli*, UniProt identifier: P0A8Y1. The sequences of all noncommercial enzymes are shown in Table S4.

[b] Enzymes used: Commercial preparation (CAS-Number 9001-59-6), from Sigma-Aldrich as ammonium sulfate suspension.

[c] The tag was always placed at the N-terminus of the enzyme. His_6, hexahistidine tag.

[d] For experimental details, see the subsection "Enzyme production" under "Experimental procedures".

[e] For experimental details, see the subsection "Enzyme assays" under "Experimental procedures".

[f] The total turnover number (TTN) was calculated according to equation 1. For experimental details, see the subsection "Operational stability" under "Experimental procedures".

n/a, not applicable; n.a. not analyzed; n.d., not detected.

Table S2. Individual reaction step E factors for the syntheses performed from U (**2**).

Product	E factor[c]				
	ΨMP (**1a**) or Ψ (**1c**)	AcP	Meth.	Phos.	Σ
ΨTP (**1c**)	2.4 (ΨMP)	35	n.a.	15	53
m^1ΨTP (**3c**)[a]	2.4 (ΨMP)	35	2397	77	2512
m^1ΨTP (**3c**)[b]	4.7 (Ψ)	n/a	5479	133	5617

[a] Synthesized via route Scheme 2a (U→ΨMP→m^1ΨMP→m^1ΨTP).

[b] Synthesized via route Scheme 2b (U→Ψ→m^1Ψ→m^1ΨTP).

[c] E factor including all solvents used in reaction/purification steps (enzyme production and final ion exchange chromatography used for product isolation were omitted). AcP, acetyl phosphate synthesis; Meth., methylation (all required reactions included), Phos., phosphorylation; Σ, sum of all steps; n/a, not applicable.

Table S3. Individual reaction step simple E factors for the syntheses performed from U (**2**).

Product	sE factor[c]				
	ΨMP (**1a**) or Ψ (**1c**)	AcP	Meth.	Phos.	Σ
ΨTP (**1c**)	0.02 (ΨMP)	2.7	n.a.	1.2	3.8
m^1ΨTP (**3c**)[a]	0.02 (ΨMP)	2.7	25.2	2.9	31
m^1ΨTP (**3c**)[b]	0.20 (Ψ)	n/a	18.6	7.2	26

[a] Synthesized via route Scheme 2a (U→ΨMP→m^1ΨMP→m^1ΨTP).

[b] Synthesized via route Scheme 2b (U→Ψ→m^1Ψ→m^1ΨTP).

[c] sE factor excluding all solvents. AcP, acetyl phosphate synthesis; Meth., methylation (all required reactions included), Phos., phosphorylation; Σ, sum of all steps; n/a, not applicable.

Table S4. Sequences of the enzymes used in this study.

Enzyme[a]	Amino acid sequence
DeoB	MGSSHHHHHHSSGLVPRGSHMKRAFIMVLDSFGIGATEDAERFGDVGADTLGHIAEACAKGEADNGRK<br>GPLNLPNLTRLGLAKAHEGSTGFIPAGMDGNAEVIGAYAWAHEMSSGKDTPSGHWEIAGVPVLFEWGYF<br>SDHENSFPQELLDKLVERANLPGYLGNCHSSGTVILDQLGEEHMKTGKPIFYTSADSVFQIACHEETFGLD<br>KLYELCEIAREELTNGGYNIGRVIARPFIGDKAGNFQRTGNRHDLAVEPPAPTVLQKLVDEKHGQVVSVGKI<br>ADIYANCGITKKVKATGLDALFDATIKEMKEAGDNTIVFTNFVDFDSSWGHRRDVAGYAAGLELFDRRLPE<br>LMSLLRDDDILILTADHGCDPTWTGTDHTREHIPVLVYGPKVKPGSLGHRETFADIGQTLAKYFGTSDMEY<br>GKAMF
YeiN	MGSSHHHHHHSSGLVPRGSHMSELKISPELLQISPEVQDALKNKKPVVALESTIISHGMPFPQNAQTAIEV<br>EETIRKQGAVPATIAIIGGVMKVGLSKEEIELLGREGHNVTKVSRRDLPFVVAAGKNGATTVASTMIIAALAGI<br>KVFATGGIGGVHRGAEHTFDISADLQELANTNVTVVCAGAKSILDLGLTTEYLETFGVPLIGYQTKALPAFF<br>CRTSPFDVSIRLDSASEIARAMVVKWQSGLNGGLVVANPIPEQFAMPEHTINAAIDQAVAEAEAQGVIGKE<br>STPFLLARVAELTGGDSLKSNIQLVFNNAILASEIAKEYQRLAG
YjjG	MGSSHHHHHHSSGLVPRGSHMKWDWIFFDADETLFTFDSFTGLQRMFLDYSVTFTAEDFQDYQAVNKP<br>LWVDYQNGAITSLQLQHGRFESWAERLNVEPGKLNEAFINAMAEICTPLPGAVSLLNAIRGNAKIGIITNGF<br>SALQQVRLERTGLRDYFDLLVISEEVGVAKPNKKIFDYALEQAGNPDRSRVLMVGDTAESDILGGINAGLA<br>TCWLNAHHREQPEGIAPTWTVSSLHELEQLLCKH
UP	MGSSHHHHHHSSGLVPRGSHMSKSDVFHLGLTKNDLQGATLAIVPGDPDRVEKIAALMDKPVKLASHRE<br>FTTWRAELDGKPVIVCSTGIGGPSTSIAVEELAQLGIRTFLRIGTTGAIQPHINVGDVLVTTASVRLDGASLH<br>FAPLEFPAVADFECTTALVEAAKSIGATTHVGVTASSDTFYPGQERYDTYSGRVVRHFKGSMEEWQAMGV<br>MNYEMESATLLTMCASQGLRAGMVAGVIVNRTQQEIPNAETMKQTESHAVKIVVEAARRLL
CMPK*	MASWSHPQFEKGLINHMTAIAPVITIDGPSGAGKGTLCKAMAEALQWHLLDSGAIYRVLALAALHHHVDVA<br>SEDALVPLASHLDVRFVSTNGNLEVILEGEDVSGEIRTQEVANAASQVAAFPRVREALLRRQRAFRELPGL<br>IADGRDMGTVVFPDAPVKIFLDASSEERAHRRMLQLQEKGFSVNFERLLAEIKERDDRDRNRAVAPLVPA<br>ADALVLDSTTLSIEQVIEKALQYARQKLALA
UMPK	MGSSHHHHHHSSGENLYFQGHMTAATTSQPAFSPDQVSVIFVLGGPGAGKGTQCEKLVKDYSFVHLSAG<br>DLLRAEQGRAGSQYGELIKNCIKEGQIVPQEITLALLRNAISDNVKANKHKFLIDGFPRKMDQAISFERDIVE<br>SKFILFFDCPEDIMLERLLERGKTSGRSDDNIESIKKRFNTFKETSMPVIEYFETKSKVVRVRCDRSVEDVY<br>KDVQDAIRDSL
AcK	MGSSHHHHHHSSGLVPRGSHMSSKLVLVLNCGSSSLKFAIIDAVNGEEYLSGLAECFHLPEARIKWKMDG<br>NKQEAALGAGAAHSEALNFIVNTILAQKPELSAQLTAIGHRIVHGGEKYTSSVVIDESVIQGIKDAASFAPLH<br>NPAHLIGIEEALKSFPQLKDKNVAVFDTAFHQTMPEESYLYALPYNLYKEHGIRRYGAHGTSHFYVTQEAAK<br>MLNKPVEELNIITCHLGNGGSVSAIRNGKCVDTSMGLTPLEGLVMGTRSGDIDPAIIFHLHDTLGMSVDAIN<br>KLLTKESGLLGLTEVTSDCRYVEDNYATKEDAKRAMDVYCHRLAKYIGAYTALMDGRLDAVVFTGGIGENA<br>AMVRELSLGKLGVLGFEVDHERNLAARFGKSGFINKEGTRPAVVIPTNEELVIAQDASRLTA

[a] See Table S1 for further specifications of the enzymes used. Note: the tag is always placed at the N-terminus. Except for *CMPK that harbors Strep-Tag II, all other enzymes contain His_6-tag.

Methods

General aspects

Chemicals. Commercially available reagents and solvents were purchased from Sigma Aldrich, TCI, Alfa Aesar, Roth, Lactan, abcr, VWR, ThermoFisher Scientific or Acros Organics and were used without further purification, unless otherwise mentioned. Experiments were usually carried out under air with non-dried solvents, unless otherwise mentioned. When applying Schlenk techniques the glass apparatus was dried under oil pump vacuum by heating with a heat gun, cooled to room temperature, and flushed with inert gas. Dry solvents were prepared by the below-mentioned procedures and afterwards stored under inert gas atmosphere (argon) over molecular sieves. The stated temperatures generally refer to the oil bath or the cooling bath temperature. The water bath temperature of the rotary evaporator was usually set to 40 °C unless otherwise noted.

Genetic reagents. Expression vectors (pET15b or pET28a(+)) containing the genes for YeiN, DeoB, UP, YjjG, AcK, and UMPK were codon-optimized for *E. coli* and ordered from Genescript.

Other. ChatGPT v3.5 was utilized to assist with rephrasing and ensuring the formal correctness of the writing. Prompts used were “check language” and “rephrase”.

Analytical methods

High performance liquid chromatography with mass spectrometry

Analytical HPLC-MS measurements were performed on an Agilent Technologies 1200 Series system (G1379 Degasser, G1312 Binary Pump, G1367C HiP ALS SL Autosampler, G1330B FC/ALS Thermostat, G1316B TCC SL column compartment, G1365C MWD SL multiple wavelength detector (deuterium lamp, 190-400 nm) equipped with a single quadrupole LC-MS detector “6120 LC/MS” using electrospray ionization source (ESI in positive and negative mode). Separations were carried out on a C-18-Reversed-Phase column of the type „Poroshell® 120 SB-C18, 3.0 x 100 mm, 2.7 µm“ by Agilent Technologies. Flow: Constant flow rate 0.7 mL min^{-1}, T = 35 °C. The following method was used:

MeCN_2_100: 0.0 – 0.1 min, isocratic, 2% MeCN (98% H_2O + 0.05% TFA); 0.1 – 8.0 min, linear, 2% to 100% MeCN (98% to 0% H_2O + 0.05% TFA); 8.0 – 11.1 min, isocratic, 100% MeCN; 11.1 –

11.3 min, linear, 100% to 2% MeCN (0% to 98% H_2O + 0.05 % TFA); 11.3 – 12.0 min, isocratic, 2% MeCN (98% H_2O + 0.05% TFA).

Samples from enzymatic reactions were analyzed by reversed-phase ion-pairing HPLC. Five µL of sample containing an overall nucleoside concentration of about 1 mM were loaded on a Kinetex C18 column (Phenomenex; 5 µm, 100 Å, 50 × 4.6 mm). Analytes were separated in 5-min long isocratic runs using 20 mM phosphate buffer, pH 5.9, containing 40 mM tetra-*n*-butylammonium bromide and 12.5% acetonitrile. The flow rate was 2.0 mL min^{-1} and the temperature set to 35 °C. Eluents were detected at 260 nm.

Nuclear magnetic resonance spectroscopy

NMR spectra were recorded on a Bruker Avance III 300 spectrometer (^{1}H: 300.36 MHz; ^{13}C: 75.53 MHz) with autosampler, or a Varian Unity Inova 500 spectrometer (^{1}H: 499.87 MHz; ^{13}C: 125.69 MHz, ^{31}P: 202.35 MHz), or a Jeol JNM-ECZL 400 MHz NMR Spectrometer (^{1}H: 399.78 MHz, ^{13}C: 100.53 MHz, ^{31}P: 161.83 MHz).

Chemical shifts δ are referenced to the residual proton and carbon signal of the deuterated solvent ($CDCl_3$: δ = 7.26 ppm (^{1}H), 77.16 ppm (^{13}C); CD_3OD: δ = 3.31 ppm (^{1}H), 49.00 ppm (^{13}C); D_2O: δ = 4.79 ppm (^{1}H)).[1] Chemical shifts δ are given in ppm (parts per million) and coupling constants *J* in Hz (Hertz). If necessary, 1D spectra (APT) as well as 2D spectra (H,H-COSY, HSQC, HMBC) were recorded for the identification and confirmation of the structure. Signal multiplicities are abbreviated as s (singlet), br s (broad singlet), d (doublet), t (triplet), q (quartet), quint (quintet), m (multiplet), dd (doublet of doublets), td (triplet of doublets), dt (doublet of triplets), and qd (quartet of doublets). Deuterated solvents for nuclear resonance spectroscopy were purchased from euriso-top®.

High resolution mass spectrometry

High-resolution mass (HRMS) spectra (LC-ESI-MS/MS) were acquired by data-dependent high-resolution tandem mass spectrometry on a QExactive Focus (Thermo Fisher Scientific). The electrospray ionization potential was set to +3.5 or -3.0 kV, the sheath gas flow was set to 20, and an auxiliary gas flow of 5 was used. Samples were diluted with an appropriate solvent (methanol or chloroform) and 1 µL was injected on a SeQuant® ZIC®-pHILIC HPLC column (Merck, 100 x 2.1 mm; 5 µm; 100 Å; peek coated; equipped with a guard column) or on a RP-column

(Waters, ACQUITY UPLC HSS T3 150 x 2.1 mm; 1.8 µm with VanGuard column). The separation solvent (pHILIC: A: CH_3CN, B: 25 mM NH_4HCO_3; RP: A: 0.1% HCOOH, B: 0.1% HCOOH in CH_3CN) was delivered through an Ultimate 3000 HPLC system (Thermo Fisher Scientific) with a flow rate of 100 µL min^{-1} and appropriate gradients were used for proper sample elution.

Determination of melting points

Melting points were determined on a Mel-Temp® melting point apparatus (Electrothermal) with an integrated microscopical support. They were measured in open capillary tubes with a mercury-in-glass thermometer and were not corrected.

Thin layer chromatography

Analytical thin layer chromatography (TLC) was carried out on Merck TLC silica gel aluminum sheets (silica gel 60, F254, 20 x 20 cm). All separated compounds were visualized by UV light (λ = 254 nm and/or λ = 366 nm) and by the listed staining reagents followed by development in heat.

<u>$KMnO_4$:</u> 3.0 g $KMnO_4$ and 20 g K_2CO_3 were dissolved in 300 mL H_2O and afterwards 5.0 mL 5% aq. NaOH were added.

Normal phase flash column chromatography

Normal phase flash column chromatography was performed on silica gel 60 (Acros Organics) with particle sizes between 35 µm and 70 µm. Depending on the problem of separation, a 30 to 100-fold excess of silica gel was used with respect to the dry amount of crude material. The dimension of the column was adjusted to the required amount of silica gel and formed a pad between 10 cm and 30 cm. In general, the silica gel was mixed with the eluent and the column was equilibrated. Subsequently, the crude material was dissolved in the eluent and loaded onto the top of the silica gel and the mobile phase was forced through the column using a rubber bulb pump. The volume of each collected fraction was adjusted between 20% and 30% of the silica gel volume.

Reversed phase flash column chromatography

Reversed phase flash column chromatography was performed on silica gel 60 C18 (Roth) with particle sizes between 35 µm and 70 µm. Depending on the problem of separation, a 30 to 100-fold excess of silica gel was used with respect to the dry amount of crude material. The dimension of the column was adjusted to the required amount of silica gel and formed a pad between 10 cm and 30 cm. In general, the silica gel was mixed with the eluent and the column was equilibrated. Subsequently, the crude material was dissolved in the eluent and loaded onto the top of the silica gel and the mobile phase was forced through the column using a rubber bulb pump. The volume of each collected fraction was adjusted between 20% and 30% of the silica gel volume.

Determination of the concentration of nucleosides, nucleotides and enzymes

Absorbance was measured with a DeNovix DS-11 Spectrophotometer and concentrations were calculated with the molar extinction coefficient. Ura: (258 nm, 8.3 $mM^{-1}cm^{-1}$); U (**2**), UMP, UDP, UTP: (262 nm, 10 $mM^{-1}cm^{-1}$); Ψ (**1**), ΨMP (**1a**), ΨDP (**1b**), ΨTP (**1c**): (262 nm, 7.5 $mM^{-1}\,cm^{-1}$); m^1Ψ (**3**), m^1ΨMP (**3a**), m^1ΨDP (**3b**), m^1ΨTP (**3c**): (260 nm, 5.8 $mM^{-1}\,cm^{-1}$).

Extinction coefficients for the enzymes used were calculated using ProtParam.[2] YeiN: (280 nm, 10,095 $M^{-1}\,cm^{-1}$; 35,073 Da), DeoB: (280 nm, 44,265 $M^{-1}\,cm^{-1}$; 46,533 Da), YjjG: (280 nm, 40,575 $M^{-1}\,cm^{-1}$; 27,464 Da), UP: (280 nm, 17,085 $M^{-1}\,cm^{-1}$; 29,322 Da), CMPK: (280 nm, 13,980 $M^{-1}\,cm^{-1}$; 26,611 Da), UMPK: (280 nm, 7,450 $M^{-1}\,cm^{-1}$; 25,339 Da), AcK (280 nm, 25,245 $M^{-1}\,cm^{-1}$; 45,453 Da).

Experimental procedures

Structure overview

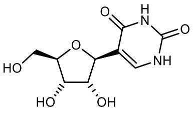

Ψ (**1**)

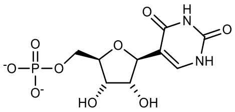

ΨMP (**1a**)

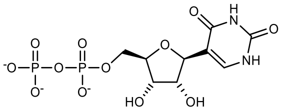

ΨDP (**1b**)

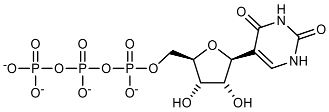

ΨTP (**1c**)

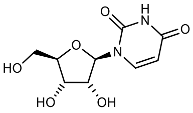

U (**2**)

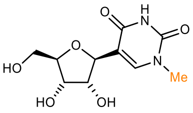

m^{1}Ψ (**3**)

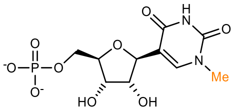

m^{1}ΨMP (**3a**)

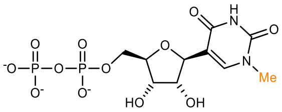

m^{1}ΨDP (**3b**)

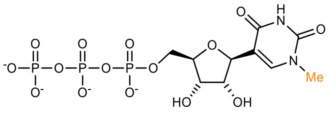

m^{1}ΨTP (**3c**)

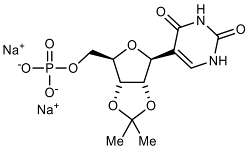

4

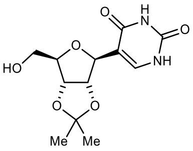

5

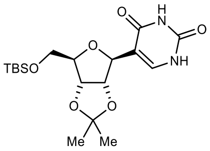

6

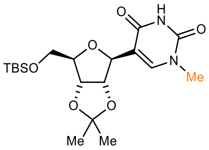

7

Enzyme production

N-terminally His$_6$-tagged DeoB, YeiN, UP, YjjG, and UMPK were utilized. CMPK harbored N-terminal Strep-Tag II (see Table S4). The e×pression vectors (pET-STREPII_*cmpk*, pET15b_*yein*, pET15b_*up*, pET15b_*deob*, pET15b_*ack*, pET28a(+)_*yjjg*, and pET28a(+)_*umpk* were used to transform *E. coli* BL2 (DE3)pLysE. Transformants were selected on LB-agar plates containing 0.1 mg mL^{-1} ampicillin, except for UMPK, YjjG and CMPK, which were selected on plates containing 0.05 mg mL^{-1} kanamycin. Enzymes were expressed in 1-L baffled shake flasks at 37 °C and 110 rpm, using 250 mL LB medium with 0.1 mg mL^{-1} ampicillin. For UMPK, YjjG and CMPK expression, 0.05 mg mL^{-1} kanamycin was used instead. Cultures were inoculated to an OD$_{600}$ of 0.1 and grown until reaching an OD$_{600}$ of ~0.8, at which point the temperature was reduced to 18 °C, and expression was induced with 0.4 mM IPTG for 20 h. Cells were harvested by

centrifugation at 4420 *g* for 30 min at 4 °C using a Sorvall RC-5B refrigerated superspeed centrifuge (Du Pont Instruments), and the supernatant was discarded.

Cell pellets containing His_6-tagged proteins were resuspended in His-tag binding buffer (50 mM HEPES, pH 8.0, containing 500 mM NaCl, 30 mM imidazole and 5% glycerol, v/v). The cell pellet containing CMPK was resuspended in a different buffer recommended for purification via Strep-Tag II (Strep-wash buffer: 100 mM Tris-Cl, pH 8.0, 150 mM NaCl, 1.0 mM EDTA). Cells were disrupted by sonication (Fisherbrand Sonic Dismembrator, Ultrasonic Processor FB-505; Fisher Scientific) for 6 min on ice, and the cell-free supernatant was collected by centrifugation at 27,150 *g* for 50 min at 4 °C.

His_6-tagged proteins were purified using immobilized metal affinity chromatography. Cell lysate (20 mL) filtered through a 0.45 µm syringe filter (Satorius) was loaded onto two 5 mL HisTrap FF columns (Cytiva) equilibrated with His-tag binding buffer and mounted on an ÄKTA prime plus system (Cytiva). Purification was conducted at 10 °C with a flow rate of 3 mL min^{-1}. Protein elution was achieved using an imidazole gradient from 0% to 100% with His-tag elution buffer (50 mM HEPES, pH 8.0, 500 mM NaCl, 300 mM imidazole).

For CMPK isolation, Strep-tactin purification was performed. The cell-free extract harboring CMPK (20 mL) was loaded at 3 mL min^{-1} onto two 5 mL StrepTrap HP columns (GE Healthcare) equilibrated with Strep-wash buffer and mounted on an ÄKTA prime plus system. Non-specifically bound proteins were washed with 10 column volumes of wash buffer, and the target protein was eluted using Strep-elution buffer (100 mM Tris-Cl, pH 8.0, 150 mM NaCl, 1.0 mM EDTA, 2.5 mM desthiobiotin).

Fractions containing the target protein were pooled, concentrated, and buffer-exchanged using Amicon Ultra-15 Centrifugal Filter Units (Millipore). The final protein concentration was adjusted to 20–50 mg mL^{-1} in 50 mM HEPES buffer (pH 7.0) containing 5% glycerol (v/v), 200 mM NaCl, and 2.0 mM $MgCl_2$. The enzyme was stored at -20 °C until further use. Protein purification was monitored by sodium dodecyl sulfate polyacrylamide gel electrophoresis (Figure S14).

Enzyme assays

All reactions were performed in 50 mM TAPS buffer (pH 8.5) containing 10 mM $MgCl_2$ at 30 °C in a total volume of 300 µL. Incubations were conducted using an Thermomixer (Eppendorf) with gentle agitation at 350 rpm. The specific substrates and concentrations used for each enzyme are detailed below. Enzyme activities were determined under substrate-saturating conditions.

Reactions were initiated by adding a concentrated enzyme solution (≤ 2% of the total volume) to the temperature-equilibrated substrate solution. Samples (20 µL) were taken at specific time points, quenched with 100 µL methanol (1/1, v/v), centrifuged for 5 min at 27,150 *g*, and analyzed by HPLC. Initial rates were derived from the linear range of product formation (or substrate consumption) under conditions of ≤ 20% substrate conversion. One unit (*U*) is defined as the amount of enzyme required to produce 1 µmol of product or consume 1 µmol of substrate per minute under the assay conditions. Specific activity is calculated from the initial reaction rate (*U* mL^{-1}) divided by the enzyme concentration used (mg mL^{-1}).

YeiN: Initial rates were assessed using 15 mM uracil and 15 mM D-ribose 5-phosphate (Rib5P) with 0.025 mg mL^{-1} enzyme. YeiN showed a specific activity of 73 *U* mg^{-1}. Activities (*U*) refer to ΨMP (**1a**) released.

UP: Initial rates were measured using 15 mM uridine (U, **2**) and 40 mM phosphate with 1.2×10^{-3} mg mL^{-1} enzyme. UP displayed a specific activity of 56 *U* mg^{-1}. Activities (*U*) refer to uracil released.

DeoB: An enzyme-coupled assay was used to measure DeoB activity. Initial rates were assessed with 1.0 mM uracil and 3.0 mM Rib1P, using 1.5 mg mL^{-1} YeiN and 1.6×10^{-3} mg mL^{-1} DeoB. Under these conditions, DeoB is rate-limiting, so the ΨMP (**1a**) formation rate equals the Rib1P isomerization rate. DeoB had a specific activity of 10 *U* mg^{-1}. Activities (*U*) refer to ΨMP (**1a**) (= Rib5P) released.

YjjG: Activity was assessed using 15 mM ΨMP (**1a**) with 6.6×10^{-3} mg mL^{-1} enzyme. YjjG exhibited a specific activity of 30 *U* mg^{-1}. Activities (*U*) refer to Ψ (**1**) or phosphate released.

AcK: Activity was measured using 30 mM acetyl phosphate and 10 mM nucleotide diphosphate (ADP, UDP, ΨDP (**1b**) or m^1ΨDP (**3b**)) with 5.0×10^{-5} to 5.0×10^{-3} mg mL^{-1} enzyme. AcK showed specific activities of 40 *U* mg^{-1} (ΨDP (**1b**)), 2.0×10^{2} *U* mg^{-1} (m^1ΨDP (**3b**)), 2.3×10^{3} *U* mg^{-1} (ADP), and 1.0×10^{2} *U* mg^{-1} (UDP). Activities (*U*) refer to nucleotide diphosphate released.

UMPK: An enzyme-coupled assay was used to measure UMPK activity. Initial rates were assessed with 10 mM nucleotide monophosphate (UMP; ΨMP (**1a**), m^1ΨMP(**3a**)), 0.5 mM ATP, 35 mM AcP, using 1.0 mg mL^{-1} AcK and 0.004 mg mL^{-1} UMPK. Under these conditions, UMPK is rate-limiting, so the ΨTP (**1c**) formation rate equals the ΨMP (**1a**) phosphorylation rate. UMPK displayed specific activities of 99 *U* mg^{-1} (ΨMP (**1a**)), 2.0×10^{2} *U* mg^{-1} (UMP), and 0.3 *U* mg^{-1} (m^1ΨMP (**3a**)). Activities (*U*) refer to nucleotide triphosphate (under condition used equal to nucleotide diphosphate) released.

CMPK: CMPK activity was assessed as described for UMPK. CMPK showed specific activities of 0.11 *U* mg^{-1} (ΨMP (**1a**) and 20 *U* mg^{-1} (UMP). Activities (*U*) refer to nucleotide triphosphate (under condition used equal to nucleotide diphosphate) released.

CMPK-PK cascade phosphorylation

The reaction mixture contained 20 mM ΨMP (**1a,** as synthesized), 2.0 mM $MgCl_2$, 60 mM PEP, 5.0 mM ATP, 0.2 mg mL^{-1} PK (70 *U* mL^{-1}), 3.0 mg mL^{-1} CMPK (0.3 *U* mL^{-1}) and was incubated at 30 °C with 350 rpm agitation in a total volume of 500 µL.

UMPK-AcK cascade phosphorylation

The reactions were conducted at 30 °C with 350 rpm agitation in a total volume of 400 µL, containing 50 mM TAPS (pH 8.5), 100 mM ΨMP (**1a**), 5.0 mM ATP, 350 mM AcP, 10 mM $MgCl_2$, 0.08 mg mL^{-1} AcK, and 0.03 mg mL^{-1} UMPK. Samples (20 µL) were taken at defined time points and quenched with an equal volume of methanol and analyzed by HPLC. Key reaction parameters were systematically varied to optimize the cascade. Temperature was adjusted from 30 °C to 50 °C in 5 °C increments; the pH was changed from 7.0 to 9.0 in 0.5-unit steps; and the $MgCl_2$ concentration was varied between 0.1 mM, 1.0 mM, and then 5.0 to 20 mM in 5.0 mM increments. The ATP concentration was varied from 0.5 mM to 10 mM, and the AcP to ΨMP (**1a**) molar ratio was tested at 2.0, 2.5, 3.0, and 4.0. The AcK concentration was increased from 0.01 to 0.05 mg mL^{-1}, and to 0.08 and 0.16 mg mL^{-1}.

The optimized reaction conditions (50 mM TAPS, pH 8.5, 15 mM $MgCl_2$, 100 mM ΨMP (**1a**), 5.0 mM ATP, 250 mM AcP, 0.08 mg mL^{-1} AcK, and 0.03 mg mL^{-1} UMPK) were used as a basis for intensification of the reaction in terms of product formed. The ΨMP (**1a**) concentration was increased to 200 mM in 50 mM increments in separate reactions, while the enzyme loading as well as the concentrations of ATP, $MgCl_2$ and AcP were linearly increased to maintain a constant ratio, as defined by the optimized conditions.

Operational stability

The stability of AcK and UMPK were analyzed under the conditions of the pH-controlled reaction as defined here. The conditions used were as follows: AcK (0.3 mg mL^{-1}) or UMPK (2.0 mg mL^{-1}),

Rib (0.95 M), AcP (1.15 M), $MgCl_2$ (30 mM); 30 °C, pH 7.5, 0.5 mL of total volume. Incubations were done in 1.5 mL microcentrifuge tubes (Sarstedt) at 30 °C using a ThermoMixer C (Eppendorf) without agitation applied. The pH was measured every 20 min and maintained with manual additions of 5 M NaOH as required. Samples (50 µL) were taken at certain times and immediately diluted (10-fold) into the reaction mixture for enzyme assay. Stability of YeiN was analyzed under the conditions of condensation reaction. For incubation the following conditions were used: YeiN (6.5 mg mL^{-1}), 50 mM HEPES buffer (pH 7.5), uracil (Ura) (0.65 M); 30 °C, 0.5 mL total volume. Incubation was performed in 2.0 mL microcentrifuge tubes (Sarstedt) at 30 °C using water bath with magnetic stirring (600 rpm). At certain times Ura was centrifuged off (16,000 *g*, 1 min) and clear supernatant (20 µL) was diluted (10-fold) in the reaction mixture for enzyme assay.

Activities were determined as described in this Supplementary Information under Activity assays (2.5). The logarithm of residual activity was plotted versus time and the slope of linear fit was used to obtain a first-order inactivation constant k_d. The total turnover number (TTN) of the enzyme was calculated with Eq. (1).

$$TTN=k_{cat}/k_d \qquad (1)$$

Molecular docking of UDP, ΨDP (1b) and m¹ΨDP (3b) to UMPK

Saccharomyces cerevisiae UMPK–product complexes were modeled based on the crystal structure of UMP/CMP kinase from *Dictyostelium discoideum* in complex with P1-(5'-adenosyl) P5-(5'-uridyl) pentaphosphate and Mg^{2+} (PDB ID: 1UKE), and the structure of *Saccharomyces cerevisiae* UMPK bound to ADP (PDB ID: 1UKY). These two kinases share 52% sequence identity. The structures were superimposed, and the UDP moiety, together with two water molecules essential for substrate coordination and Mg^{2+}, was positioned in the binding pocket of *S. cerevisiae* UMPK to replicate the binding configuration observed in the *D. discoideum* enzyme. To generate the ΨDP (**1b**) and m¹ΨDP (**3c**) complexes, UMP was converted into the respective ligands using Yasara version 18.2.7. The generated complexes were energy-minimized in Yasara utilizing the AMBER-15FB force field.[3] For evaluation, local docking was conducted using the standard macro provided in Yasara. Molecular docking experiments were performed with AutoDock VINA[4] as implemented within Yasara v. 18.2.7. A simulation cell measuring 10 Å × 10 Å × 10 Å encompassed the bound UDP binding site, and 50 docking runs were executed. Results were visualized using PyMOL 2.5.4, revealing that the docking poses aligned well with the energy-minimized modeled complex.

Enzymatic synthesis of Ψ (1) and ΨMP (1a)

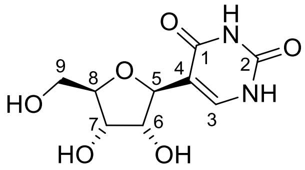

1

For Ψ (**1**) synthesis, 0.1 M sodium phosphate buffer (Na_2HPO_4, 0.05 g; NaH_2PO_4, 0.02 g), 1.0 M U (**2**) (1.22 g, 5 mmol) and 2.0 mM $MnCl_2$ (0.002 g) were dissolved in doubly distilled H_2O. The nucleoside content was monitored at 260 nm. The reaction was started by addition of 0.3 mg mL^{-1} UP, 2.5 mg mL^{-1} DeoB, 1.5 mg mL^{-1} YeiN, and 0.3 mg mL^{-1} YjjG and incubated in a water bath at 30 °C for 7 h and stirred at 700 rpm using a magnet stirrer with integrated heating plate (RCT basic, IKA). Pure Ψ (**1**) crystallized from the reaction and could be isolated by centrifugation. The solid material was freeze-dried using a Christ Alpha 1-4 freeze dryer (bbi-biotech GmbH) connected to a Vacuubrand vacuum pump unit RZ 6.

Yield 1.10 g (4.5 mmol, 90%), colorless solid, $C_9H_{12}N_2O_6$ [244.20]

^{1}H NMR (300.36 MHz, D_2O) δ = 7.66 (s, 1H, H-3), 4.68 (d, $^3J_{HH}$ = 3.9 Hz, 1H, H-5), 4.29 (s, 1H, H-6), 4.14 (s, 1H, H-7), 4.02 (s, 1H, H-8), 3.90 – 3.68 (m, 2H, H-9) ppm.

^{13}C NMR (75.53 MHz, D_2O) δ = 165.3 (C_q, C-1), 152.8 (C_q, C-2), 141.4 (CH, C-3), 110.5 (C_q, C-4), 83.4 (CH, C-8), 79.2 (CH, C-5), 73.4 (CH, C-6), 70.8 (CH, C-7), 61.5 (CH_2, C-9) ppm.

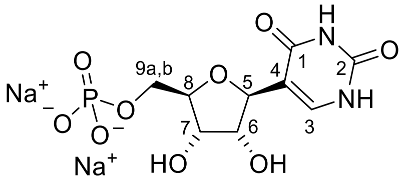

1a

For ΨMP (**1a**) synthesis, 1.0 M sodium phosphate buffer (Na_2HPO_4, 0.54 g; NaH_2PO_4, 0.15 g), 1.0 M U (**2**) (1.22 g, 5 mmol) and 20 mM $MnCl_2$ (0.01 g) were dissolved in doubly distilled H_2O. The nucleoside content was monitored at 260 nm. The reaction was started by addition of 0.25 mg mL^{-1} UP, 2.5 mg mL^{-1} DeoB, and 1.5 mg mL^{-1} YeiN and incubated at 40 °C for 20 h in a shaking

water bath 1083 (GFL) with 700 rpm agitation. Enzymes were separated from the reaction mixture using an Amicon Ultra-15 Centrifugal Filter Unit (Millipore) with a 10 kDa molecular mass cut-off. The resulting supernatant was freeze-dried using a Christ Alpha 1-4 freeze dryer (bbi-biotech GmbH) connected to a RZ 6 vacuum pump (Vacuubrand).

Yield 1.90 g (4.75 mmol, 95%), colorless solid, $C_9H_{11}Na_2N_2O_9P$ [367.99]

^{1}H NMR (300.36 MHz, D_2O) δ = 7.93 (s, 1H, H-3), 4.83 (s, 1H, H-5), 4.31 – 4.25 (m, 1H, H-6), 4.24 – 4.17 (m, 1H, H-7), 4.11 – 4.00 (m, 2H, H-8, H-9a), 3.94 – 3.86 (m, 1H, H-9b) ppm.

^{13}C NMR (75.53 MHz, D_2O) δ = 166.4 (C_q, C-1), 154.3 (C_q, C-2), 142.4 (CH, C-3), 111.4 (C_q, C-4), 81.5 (d, $^3J_{CP}$ = 8.6 Hz, CH, C-8), 78.6 (CH, C-5), 74.5 (CH, C-6), 69.8 (CH, C-7), 62.7 (d, $^2J_{CP}$ = 4.4 Hz, CH_2, C-9) ppm.

^{31}P NMR (202.35 MHz, D_2O) δ = 1.6 ppm.

Enzymatic synthesis of ΨTP (1c)

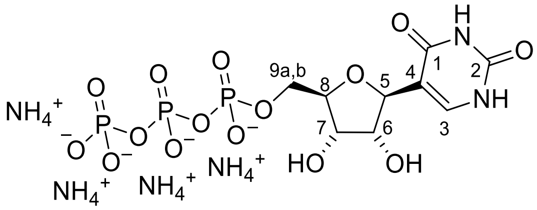

1c

The ΨTP synthesis was performed in a 15 mL Sarstedt tube. For ΨTP synthesis 50 mM TAPS (69 mg) at pH 8.5, 100 mM ΨMP (**1a**) (232 mg, 0.58 mmol, disodium salt), 10 mM $MgCl_2$ (12 mg), 5.0 mM ATP (16 mg), 300 mM AcP (1923 µL of a 900 mM aqueous stock) were combined and dissolved in doubly distilled H_2O to a total volume of 5.8 mL. The nucleoside content was monitored at 260 nm. The reaction was started by addition of 0.1 mg mL^{-1} AcK and 0.1 mg mL^{-1} UMPK. The reaction mixture was incubated at 30 °C for 3 h in a shaking water bath 1083 (GFL) with 700 rpm agitation. The pH was monitored and adjusted, if necessary, with 5 M NaOH. Samples taken (100 µL) were quenched by the addition of methanol (100 µL) and analyzed by HPLC. Enzymes were separated from the reaction mixture using an Amicon Ultra-15 Centrifugal Filter Unit (Millipore) with a 10 kDa molecular mass cut-off.

ΨTP (**1c**) was isolated from the reaction mixture by anion exchange (AEX) chromatography, using a XK26/40 column (26 x 400 mm, Cytivia) packed with 125 mL DEAE FF (Cytivia) anion exchange

resin, mounted onto an ÄKTA prime plus FPLC system (GE Healthcare) equilibrated with 5 mM NH_4HCO_3. The enzyme free reaction mixture was diluted to 20 mM ΨTP (**1c**) and applied with a flow rate of 5 mL min^{-1}. ΨTP (**1c**) was eluted with 80 mM NH_4HCO_3 at a flow rate of 10 mL min^{-1}. Elution of compounds was monitored at 260 nm. All fractions containing ΨTP (**1c**) were pooled and concentrated using rotary evaporator Laborta 4000 (Heidolph Instruments) at 40 °C and 30 mbar to a final volume of 10 mL. If samples contained residual NH_4HCO_3, ~10 mL of doubly distilled H_2O was added and the samples were re-concentrated until complete removal of NH_4HCO_3. The resulting aqueous solution was freeze-dried using a Christ Alpha 1-4 freeze dryer (bbi-biotech GmbH) connected to a RZ 6 vacuum pump (Vacuubrand).

Yield 271 mg (0.491 mmol, 84%), colorless hygroscopic solid, $C_9H_{27}N_6O_{15}P_3$ [552.26]

^{1}H NMR (499.87 MHz, D_2O) δ = 7.86 (s, 1H, H-3), 4.90 (s, 1H), 4.38 – 4.34 (m, 1H), 4.33 – 4.28 (m, 1H), 4.28 – 4.25 (m, 1H), 4.24 – 4.16 (m, 2H), 7.93 (s, 1H, H-3), 4.83 (s, 1H, H-5), 4.31 – 4.25 (m, 1H, H-6), 4.24 – 4.17 (m, 1H, H-7), 4.11 – 4.00 (m, 2H, H-8, H-9a), 3.94 – 3.86 (m, 1H, H-9b) ppm.

^{13}C NMR (125.69 MHz, D_2O) δ = 165.3 (C_q, C-1), 152.8 (C_q, C-2), 140.8 (CH, C-3), 111.6 (C_q, C-4), 80.86 (d, $^3J_{CP}$ = 9.0 Hz, CH, C-8), 78.33 (CH, C-5), 74.59 (CH, C-6), 69.65 (CH, C-7), 64.65 (d, $^2J_{CP}$ = 5.0 Hz, CH_2, C-9)

^{31}P NMR (202.35 MHz, D_2O) δ = -7.7 (1P), -10.9 (d, *J* = 18.9 Hz, 1P), -22.3 (1P) ppm.

General procedure for the 2',3'-*O*-isopropylidene protection

A suspension of Ψ (**1**) or ΨMP (**1a**) (2.0 mmol) in 2,2-dimethoxypropane (12 mL) and DMF (8 mL) was prepared in a 100 mL round-bottom flask. Concentrated HCl (160 µL) was added, and the mixture was stirred at room temperature for 80 min until complete conversion, as confirmed by HPLC, was achieved. After neutralization with 1 M aq. NaOH, the solvent was removed under reduced pressure. Purification procedure and analytical data are stated for each substrate.

Synthesis of 2',3'-*O*-isopropylidene-ΨMP (4)

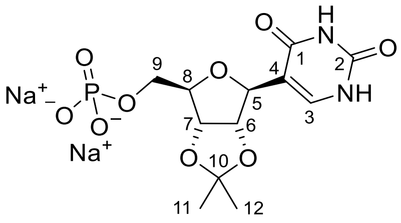

4

Starting from disodium ΨMP (**1a**) (803 mg, 2.03 mmol) 2',3'-*O*-isopropylidene protection was carried out as described in "General procedure for the 2',3'-O-isopropylidene protection". The crude product was purified via reversed-phase column chromatography (35 g C18 RP-silica gel, 7 x 3.5 cm, H_2O, fraction size: 15 mL).

Yield 820 mg (2.01 mmol, 99%), colorless solid, $C_{12}H_{15}N_2Na_2O_9P$ [408.21]

HPLC-MS t_R = 2.49 min (method: *MeCN_2_100*); *m/z* (ESI+) = 365 $[M\text{-}2Na\text{+}3H]^+$

mp 245 – 250 °C decomposition

^{1}**H NMR** (300.36 MHz, D_2O) δ = 7.78 (s, 1H, H-3), 4.96 – 4.86 (m, 3H, H-5 to H-7), 4.29 (dd, $^3J_{HH}$ = 9.4, 3.9 Hz, 1H, H-8), 3.96 – 3.79 (m, 2H, H-9), 1.61 (s, 3H, H-11 or H-12), 1.41 (s, 3H, H-11 or H-12) ppm.

^{13}**C NMR** (75.53 MHz, D_2O) δ = 166.7 (C_q, C-1), 161.0 (C_q, C-2), 144.1 (CH, C-3), 114.8 (C_q, C-10), 110.5 (C_q, C-4), 83.9 (CH, C-6), 83.8 (d, $^3J_{CP}$ = 9.0 Hz, CH, C-8), 81.5 (CH, C-7), 80.6 (CH, C-5), 63.8 (d, $^2J_{CP}$ = 4.5 Hz, CH_2, C-9), 26.4 (CH_3, C-11 or C-12), 24.5 (CH_3, C-11 or C-12) ppm.

^{31}**P NMR** (202.35 MHz, D_2O) δ = 3.92 ppm.

HRMS calcd (*m/z*) for $[C_{12}H_{16}N_2O_9P]^-$ $[M\text{-}2Na\text{+}H]^-$: 363.0594; found: 363.0601.

Synthesis of 2’,3’-*O*-isopropylidene-Ψ (5)

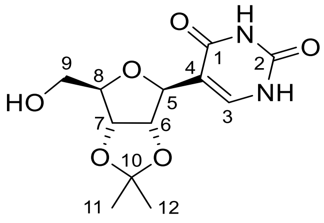

5

Starting from Ψ (**1**) (800 mg, 3.28 mmol) 2’,3’-*O*-isopropylidene protection was carried out as described in ”General procedure for the 2’,3’-O-isopropylidene protection”. The crude product was purified via reversed-phase column chromatography (35 g C18 RP-silica gel, 7 x 3.5 cm, H_2O/MeCN = 10/1, fraction size: 15 mL).

Yield 774 mg (2.72 mmol, 83%), colorless solid, $C_{12}H_{16}N_2O_6$ [284.27]

HPLC-MS t_R = 3.12 min (method: *MeCN_2_100*); *m*/*z* (ESI+) = 285 $[M+H]^+$

mp 208 – 212 °C

^{1}H NMR (300.36 MHz, D_2O) δ = 7.65 (s, 1H, H-3), 4.93 (dd, $^3J_{HH}$ = 6.4, 4.5 Hz, 1H, H-5), 4.83 (s, 1H, H-6), 4.17 (dd, $^3J_{HH}$ = 9.4, 4.2 Hz, 1H, H-8), 3.75 (qd, $^3J_{HH}$ = 12.2, 4.7 Hz, 2H, H-9), 1.61 (s, 3H, H-11 or H-12), 1.40 (s, 3H, H-11 or H-12) ppm.

^{13}C NMR (75.53 MHz, D_2O) δ = 165.5 (C_q, C-1), 153.8 (C_q, C-2), 142.6 (CH, C-3), 115.1 (C_q, C-10), 110.3 (C_q, C-4), 84.3 (CH, C-8), 83.4 (CH, C-6), 81.4 (CH, C-7), 81.0 (CH, C-5), 61.6 (CH_2, C-9), 26.4 (CH_3, C-11 or C-12), 24.5 (CH_3, C-11 or C-12) ppm.

The spectra are in accordance with the ones reported in literature.[5]

Synthesis of m[1]ΨMP (3a)

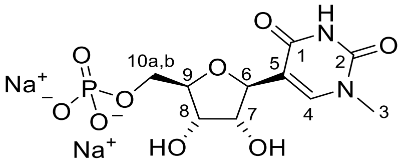

3a

In an argon-flushed 30 mL Schlenk flask disodium 2',3'-*O*-isopropylidene-ΨMP (**4**) (200 mg, 0.490 mmol) was suspended in 10 mL CH_2Cl_2. *N,O*-Bis(trimethylsilyl)acetamide (1.82 mL, 1.50 g, 7.35 mmol) was added and the reaction mixture was stirred for 24 h at room temperature. Subsequently, dimethyl sulfate (70 µL, 92.7 mg, 0.735 mmol) was added and the reaction mixture was stirred at 40 °C. After 17 h, additional dimethyl sulfate was added (70 µL, 92.7 mg, 0.735 mmol), followed by another addition of dimethyl sulfate (46.6 µL, 62.0 mg, 0.491 mmol) after stirring for additional 24 h at 40 °C. Full conversion was eventually detected by HPLC after stirring for a total of 4 d at 40 °C. After cooling to room temperature, the solvent of the dark-yellow solution was removed under reduced pressure. The residue was taken up in 10 mL H_2O, 5 mL TFA were added, and the yellowish solution was stirred for 20 min at room temperature until quantitative 2',3'-deprotection was detected by HPLC. The reaction solution was washed with 10 mL EtOAc and the aqueous layer was directly loaded on a reversed-phase silica column and the protonated intermediate was purified via reversed-phase column chromatography (35 g C18 RP-silica gel, 7 × 3.5 cm, H_2O, fraction size: 10 mL). Fractions containing the desired product were pooled and lyophilized. The residue was taken up in 5 mL H_2O and the pH was adjusted to pH 11 with 1 M aq. NaOH. The solution was directly loaded on a reversed-phase silica column and the deprotonated product was purified via reversed-phase column chromatography (35 g C18 RP-silica gel, 7 × 3.5 cm, H_2O, fraction size: 10 mL). Fractions containing the desired product were pooled and lyophilized.

Yield 159 mg (0.417 mmol, 85%), colorless solid, $C_{10}H_{13}N_2Na_2O_9P$ [382.17]

HPLC-MS t_R = 0.90 min (method: *MeCN_2_100*); *m*/*z* (ESI+) = 339 $[M-2Na+3H]^+$

mp 135 – 140 °C

^{1}H NMR (300.36 MHz, D_2O) δ = 7.69 (s, 1H, H-4), 4.14 – 4.06 (m, 2H, H-7, H-8), 3.97 (d, $^3J_{HH}$ = 3.4 Hz, 2H, H-9, H-10a), 3.85 (dd, $^3J_{HH}$ = 16.2, 8.9 Hz, 1H, H-10b), 3.26 (s, 3H, H-3) ppm.

^{13}**C NMR** (75.53 MHz, D_2O) δ = 165.0 (C_q, C-1), 152.6 (C_q, C-2), 145.7 (CH, C-4), 111.4 (C_q, C-5), 81.4 (d, $^3J_{CP}$ = 8.2 Hz, CH, C-9), 78.5 (CH, C-6), 74.3 (CH, C-8), 70.1 (CH, C-7), 64.1 (d, $^2J_{CP}$ = 4.7 Hz, CH_2, C-10), 36.3 (CH_3, C-3) ppm.

^{31}**P NMR** (202.35 MHz, D_2O) δ = 2.99 ppm.

HRMS calcd (*m/z*) for $[C_{10}H_{14}N_2O_9P]^-$ $[M-2Na+H]^-$: 337.0437; found: 377.0445.

Synthesis of 2',3'-*O*-isopropylidene-5'-*O*-(*tert*-butyldimethylsilyl)-Ψ (6)

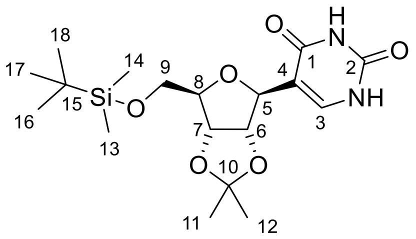

6

In an argon-flushed 30 mL Schlenk flask 2',3'-*O*-isopropylidene-Ψ (**5**) (170 mg, 0.598 mmol) was suspended in 10 mL CH_2Cl_2. Imidazole (82 mg, 1.20 mmol) and *tert*-butyldimethylsilyl chloride (180 mg, 1.20 mmol) were added and the colorless suspension was stirred for 16 h at room temperature until quantitative conversion was detected by HPLC. The solvent was removed under reduced pressure and the residue was taken up in 40 mL EtOAc. The organic layer was washed with water (1 × 30 mL) and brine (1 x 30 mL), dried over Na_2SO_4, filtered, and the solvent was removed under reduced pressure. The crude product was purified via flash chromatography (25 g silica gel, 8 × 3 cm, CH_2Cl_2/MeOH = 10/1, fraction size: 10 mL).

Yield 233 mg (0.585 mmol, 98%), colorless solid, $C_{18}H_{30}N_2O_6Si$ [398.53]

HPLC-MS t_R = 6.22 min method: *MeCN_2_100*); *m*/*z* (ESI+) = 399 $[M+H]^+$

TLC R_f = 0.43 (CH_2Cl_2/MeOH = 10/1; UV + $KMnO_4$)

mp 220 – 223 °C

^{1}**H NMR** (300.36 MHz, CD_3OD) δ = 7.43 (s, 1H), 4.78 – 4.70 (m, 2H, H-5, H-6), 4.69 – 4.63 (m, 1H, H-7), 4.03 (dd, $^3J_{HH}$ = 8.5, 4.2 Hz, 1H, H-8), 3.78 (qd, $^3J_{HH}$ = 11.2, 4.5 Hz, 2H, H-9), 1.53 (s, 3H, H-11 or H-12), 1.33 (s, 3H, H-11 or H-12), 0.91 (s, 9H, H-16 to H-18), 0.09 (s, 6H, H-13, H-14) ppm.

^{13}C NMR (75.53 MHz, CD_3OD) δ = 165.3 (C_q, C-1), 153.4 (C_q, C-2), 140.7 (CH, C-3), 115.0 (C_q, C-10), 113.1 (C_q, C-4), 86.7 (CH, C-8), 86.1 (CH, C-6), 83.2 (CH, C-7), 82.5 (CH, C-5), 64.8 (CH_2, C-9), 27.8 (CH_3, C-11 or C-12), 26.4 (CH_3, C-16 to C-18), 25.7 (CH_3, C-11 or C-12), 19.2 (C_q, C-15), -5.3 (CH_3, C-13, C-14) ppm.

HRMS calcd (*m/z*) for [$C_{18}H_{31}N_2O_6Si^+$]: 399.1952; found: 399.1939.

Synthesis of *N*¹-methyl-2',3'-*O*-isopropylidene-5'-*O*-(*tert*-butyldimethylsilyl)-Ψ (7)

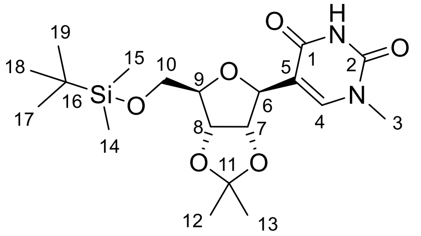

7

In a flame-dried and argon-flushed 30 mL Schlenk flask 2',3'-*O*-isopropylidene-5'-*O*-(*tert*-butyldimethylsilyl)-Ψ (**6**) (220 mg, 0.552 mmol) was suspended in 10 mL dry CH_2Cl_2. *N,O*-Bis(trimethylsilyl)acetamide (342 µL, 281 mg, 1.38 mmol) was added and the resulting colorless solution was stirred for 3 h at room temperature. Subsequently, methyl iodide (206 µL, 470 mg, 3.31 mmol) was added and the reaction mixture was stirred for 66 h at 40 °C until quantitative conversion was detected by HPLC. After cooling to room temperature, 20 mL sat. $NaHCO_3$-solution were added and the yellowish mixture was extracted with CH_2Cl_2 (3 x 30 mL). The combined organic layers were dried over Na_2SO_4, filtered and the solvent was removed under reduced pressure. The crude, yellow oil was purified via flash chromatography (50 g silica gel, 10.5 x 3.5 cm, CH_2Cl_2/MeOH = 20/1, fraction size: 25 mL).

Yield 200 mg (0.485 mmol, 88%), yellow amorphous solid, $C_{19}H_{32}N_2O_6Si$ [412.56]

HPLC-MS t_R = 6.92 min (method: *MeCN_2_100*); *m*/*z* (ESI+) = 413 [M+H]$^+$

TLC R_f = 0.31 (CH_2Cl_2/MeOH = 20/1; UV + $KMnO_4$)

^{1}H NMR (300.36 MHz, $CDCl_3$) δ = 9.08 (s, 1H, N-H), 7.33 (s, 1H, H-4), 4.85 (s, 1H, H-6), 4.65 (s, 2H, H-7, H-8), 4.08 (d, $^3J_{HH}$ = 3.3 Hz, 1H, H-9), 3.79 (ddd, $^3J_{HH}$ = 15.4, 11.2, 3.6 Hz, 2H, H-10), 3.34 (s, 3H, H-3), 1.57 (s, 3H, H-12 or H-13), 1.33 (s, 3H, H-12 or H-13), 0.89 (s, 9H, H-17 to H-19), 0.07 (s, 6H, H-14, H-15) ppm.

^{13}C NMR (75.53 MHz, $CDCl_3$) δ = 162.3 (C_q, C-1), 151.1 (C_q, C-2), 142.6 (CH, C-4), 114.2 (C_q, C-11), 113.1 (C_q, C-5), 85.2 (CH, C-9), 85.1 (CH, C-7), 81.3 (CH, C-8), 80.7 (CH, C-6), 63.4 (CH_2, C-10), 36.1 (CH_3, C-3), 27.7 (CH_3, C12 or C-13), 26.0 (CH_3, C-17 to C-19), 25.7 (CH_3, C12 or C-13), 18.5 (C_q, C-16), -5.2 (CH_3, C-14 or C-15), -5.2 (CH_3, C-14 or C-15) ppm.

HRMS calcd (*m/z*) for $[C_{19}H_{33}N_2O_6Si]^+$ $[M+H]^+$: 413.2108; found: 413.2091.

Synthesis of m^{1}Ψ (3)

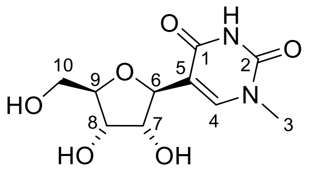

3

In a 50 mL round-bottom flask *N^1*-methyl-2',3'-*O*-isopropylidene-5'-*O*-(*tert*-butyldimethylsilyl)-Ψ (**7**) (1.02 g, 2.47 mmol) was dissolved in a mixture of TFA (10 mL) and water (15 mL) and the yellowish solution was stirred for 2 h at room temperature until full conversion was detected by HPLC. The reaction solution was concentrated under reduced pressure and lyophilized to give m^{1}Ψ (**3**) without further purification necessary.

Yield 632 mg (2.45 mmol, 99%), colorless solid, $C_{10}H_{14}N_2O_6$ [258.23]

HPLC-MS t_R = 1.52 min (method: *MeCN_2_100*); *m*/*z* (ESI+) = 259 $[M+H]^+$

mp 125 – 129 °C

^{1}H NMR (300.36 MHz, D_2O) δ = 7.74 (s, 1H, H-4), 4.65 (d, $^3J_{HH}$ = 5.5 Hz, 1H, H-6), 4.26 (t, $^3J_{HH}$ = 5.3 Hz, 1H, H-7), 4.12 (t, $^3J_{HH}$ = 5.2 Hz, 1H, H-8), 4.00 (dd, $^3J_{HH}$ = 7.6, 4.4 Hz, 1H, H-9), 3.77 (ddd, $^3J_{HH}$ = 16.9, 12.5, 3.6 Hz, 2H, H-10), 3.36 (s, 3H, H-3) ppm.

^{13}C NMR (75.53 MHz, D_2O) δ = 164.9 (C_q, C-1), 152.5 (C_q, C-2), 146.3 (CH, C-4), 110.5 (C_q, C-5), 83.4 (CH, C-9), 79.1 (CH, C-6), 73.4 (CH, C-7), 70.8 (CH, C-8), 61.4 (CH_2, C-10), 36.1 (CH_3, C-3) ppm.

Synthesis of m¹ΨTP (3c)

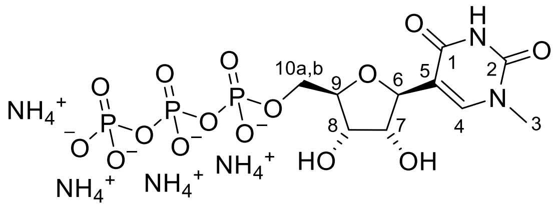

3c

In a flame-dried and argon-flushed 30 mL Schlenk flask m¹Ψ (**3**) (250 mg, 0.968 mmol) was dissolved in 5 mL trimethyl phosphate and the colorless solution was cooled to 0 °C via an ice/water bath. Phosphorous oxychloride (88.3 µL, 0.970 mmol) was added and the solution was stirred for 10 min at 0 °C. Another portion of phosphorous oxychloride (88.3 µL, 0.970 mmol) was added and the resulting yellowish solution was stirred for another 50 min at 0 °C before it was cooled to -10 °C (ice/water/NaCl bath) and stirred for 15 min at -10 °C. A pre-chilled cocktail of bis(tributylammonium) pyrophosphate (819 mg, 1.49 mmol), tributylamine (1.38 mL, 1.08 g, 5.81 mmol) and 4 mL acetonitrile was added and the reaction mixture was stirred for 30 min at -10 °C. Subsequently, the reaction was quenched by the slow addition of 20 mL H_2O and extracted with CH_2Cl_2 (3 × 15 mL). The aqueous layer was then transferred into a 100 mL round-bottom flask, adjusted to pH 6.5 by the addition of 25% NH_3-solution and stirred at 4 °C overnight. The yellow solution was again adjusted to pH 6.5 with 25% NH_3-solution and lyophilized. The crude, lyophilized product was dissolved in water to 20 mM product concentration and m¹ΨTP (**3c**) was isolated by AEX chromatography, as described for ΨTP (**1c**).

Yield 299 mg (0.528 mmol, 55%), colorless solid, $C_{10}H_{29}N_6O_{15}P$ [566.29]

mp > 130 °C decomposition

^{1}H NMR (300.36 MHz, D_2O) δ = 7.82 (s, 1H), 4.33 – 4.09 (m, 5H), 3.39 (s, 3H) ppm.

^{13}C NMR (75.53 MHz, D_2O) δ = 165.0 (C_q, C-1), 152.6 (C_q, C-2), 145.6 (CH, C-4), 111.3 (C_q, C-5), 81.2 (d, $^3J_{CP}$ = 8.4 Hz, CH, C-9), 78.22 (CH, C-6), 74.3 (CH, C-8), 69.8 (CH, C-7), 64.9 (d, $^2J_{CP}$ = 5.5 Hz, CH_2, C-10), 36.3 (CH_3, C-3) ppm.

(C_q, C-1), 152.6 (C_q, C-2), 145.7 (CH, C-4), 111.4 (C_q, C-5), 81.4 (d, $^3J_{CP}$ = 8.2 Hz, CH, C-9), 78.5 (CH, C-6), 74.3 (CH, C-8), 70.1 (CH, C-7), 64.1 (d, $^2J_{CP}$ = 4.7 Hz, CH_2, C-10), 36.3 (CH_3, C-3)

^{31}**P NMR** (161.83 MHz, D_2O) δ = -8.06 (1P), -10.39 (d, J = 18.6 Hz, 1P), -21.10 – -21.62 (m, 1P) ppm.

Biocatalytic synthesis of m^{1}ΨTP (3c)

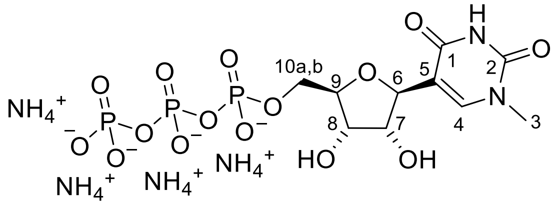

3c

The m^{1}ΨTP (**3c**) synthesis was performed in a 50 mL Sarstedt tube. For synthesis of m^{1}ΨTP (**3c**) 50 mM TAPS (206 mg), 20 mM m^{1}ΨMP (**3c**, 130 mg, 0.34 mmol, disodium salt), 10 mM $MgCl_2$ (34 mg), 1.25 mM ATP (12 mg), 75 mM AcP (1500 µL of a 900 mM aqueous stock, 1.35 mmol) were combined and dissolved in doubly distilled H_2O, the pH adjusted to pH 8.5 with 5 M NaOH and the final volume brought to 17 mL. The nucleoside content was monitored at 260 nm. The reaction was initiated by addition of 0.1 mg mL^{-1} AcK and 0.1 mg mL^{-1} UMPK. The reaction mixture was incubated at 30 °C for 3 h in a shaking water bath 1083 (GFL) at 700 rpm agitation. The pH was monitored and adjusted, if necessary, with 5 M NaOH. Samples taken (100 µL were quenched in 100 µL 1:1 v/v methanol) were analysed by HPLC. Enzymes were separated from the reaction mixture using an Amicon Ultra-15 Centrifugal Filter Unit (Millipore) with a 10 kDa molecular mass cut-off. m^{1}ΨTP (**3c**) was isolated from the reaction mixture by AEX chromatography, as described for ΨTP (**1c**).

Yield 157 mg (0.286 mmol, 84%), yellow amorphous solid, $C_{19}H_{32}N_2O_6Si$ [412.56]

Synthesis of acetyl phosphate

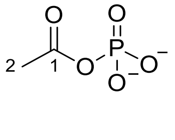

AcP

A protocol adapted from Tasnadi et al. was used.[6] Aqueous H_3PO_4 (85%, 13.5 mL, 0.2 mol) was added to 120 mL EtOAc and cooled on ice. Cold acetic anhydride (56.4 mL, 0.6 mol) was added

dropwise over 10 min. After incubating 5.5 h at 0 °C with stirring, the reaction was poured into a mixture of H_2O (100 mL) with ice (50 g) and $NaHCO_3$ (16.8 g, 0.2 mol). The slurry was incubated at 0 °C and stirred until bubbling ceased. The organic phase was discarded and the aqueous phase was washed three-times with cold EtOAc (200 mL). The pH was adjusted to 7.5 with 10 M NaOH and the solution washed again with cold EtOAc (50 mL). Subsequently, residual EtOAc in the aqueous phase was removed using a Laborota 4000 rotary evaporator (Heidolph Instruments) at 30 °C and 35 mbar for 10 min. The concentration of the aqueous AcP was determined by ^{1}H-NMR (Figure S13) relative to a 50 mM internal standard of dimethyl methylphosphonate. Inorganic phosphate impurity was identified in ~25% relative concentration by ^{31}P-NMR analysis (Figure S15). AcP was stored at -20 °C.

^{1}H NMR (300.36 MHz, D_2O) δ = 1.92 (s, 3H, H-2) ppm.

^{13}C NMR (125.65 MHz, D_2O) δ = 172.1 (d, $^2J_{CP}$ = 8.0 Hz, C_q, C-1), 22.0 (d, $^3J_{CP}$ = 4.8 Hz, CH_3, C-2) ppm.

^{31}P NMR (202.35 MHz, D_2O) δ = -2.0 ppm.

In vitro transcription

In vitro transcription (IVT) using T7 RNA polymerase (Thermo Fisher Scientific) was performed following the manufacturer’s protocol. Shortly, linear DNA template (Sequence depicted in Figure S50.) containing the T7 promoter and the *yein* gene was amplified from the YeiN expression vector (pET15b_*yein*) by PCR using Q5 polymerase and a set of primers complementary to the T7 promoter (TAATACGACTCACTATAGGG) or terminator (GCTAGTTATTGCTCAGCGG). The PCR product was purified via column purification using GenJET® PCR clean up kit (Thermo Fisher Scientific) and eluted in DEPC-water. IVT was performed using 30 *U* of T7 RNA polymerase, 500 ng template DNA, transcription Buffer (40 mM Tris-HCl (pH 7.9), 6 mM $MgCl_2$, 10 mM DTT, 10 mM NaCl and 2 mM spermidine) and 2 mM of each NTP (ATP, GTP, UTP, CTP) in a total volume of 50 µL. The negative control was performed as mentioned above with the exception that UTP was not present in the NTP mix. Synthesis of modified RNA was performed by full replacement of UTP with 2 mM of either ΨTP (**1c**) or m^{1}ΨTP (**3c**). The reaction was incubated at 37 °C in the Doppio-Dual thermocycler (VWR). IVT products were analyzed by 1% agarose gel electrophoresis stained with GelGreen (Thermo Fisher Scientific) and visualized under UV light using a Gel Doc 2000 system (Bio-Rad).

Calculation of E factor

The E factor is calculated by dividing the total mass of all waste by the mass of final product isolated including solvents used during intermediate purification (equation 2). Enzyme preparation and final product isolation were omitted from the calculations. For the calculation of the simple E factor (sE factor) solvents used were omitted from the calculation. All calculations are comprehensively outlined in the accompanying document entitled “E factor calculations”.

$$\text{(s)E factor} = \frac{mass\ product\ (g)}{mass\ waste\ (g)} \qquad (2)$$

NMR appendix

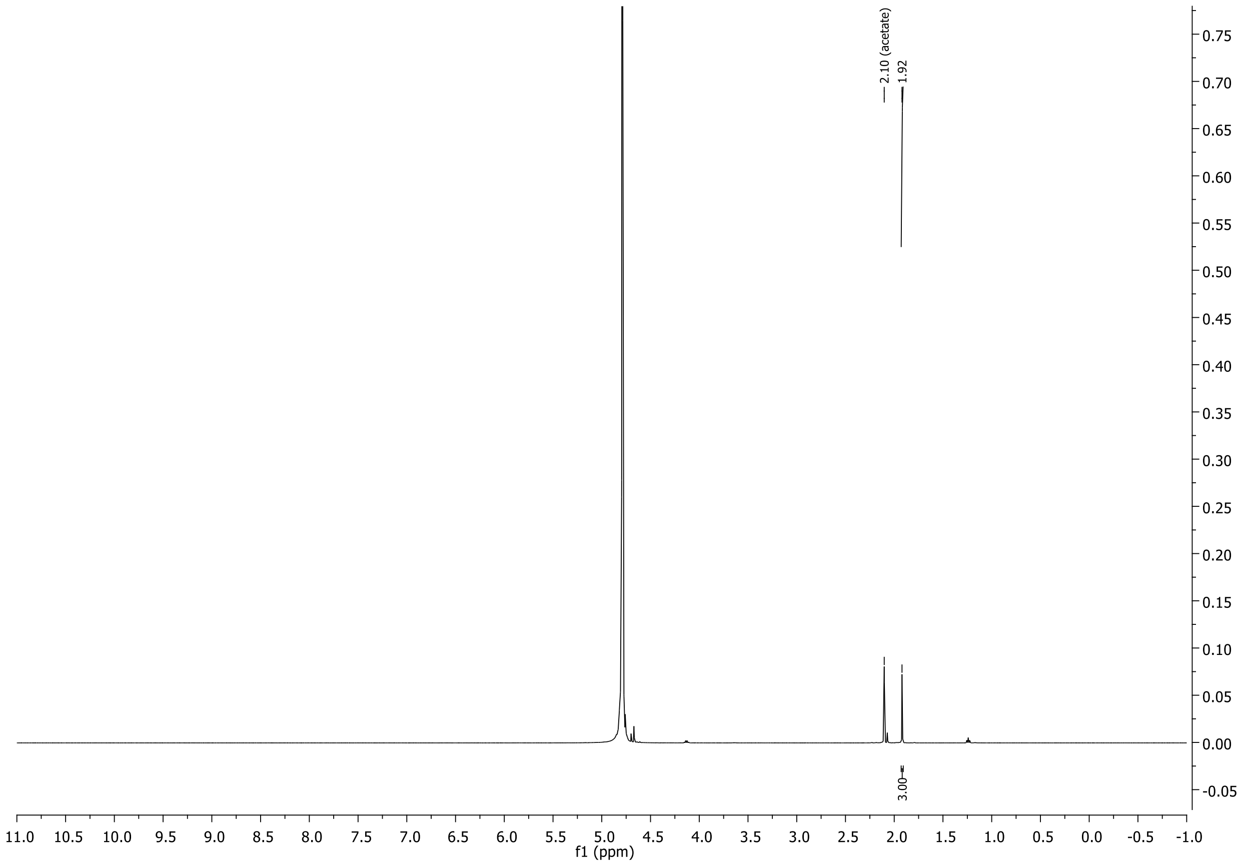

Figure S13. ^{1}H-NMR (499.87 MHz, D_2O) of AcP.

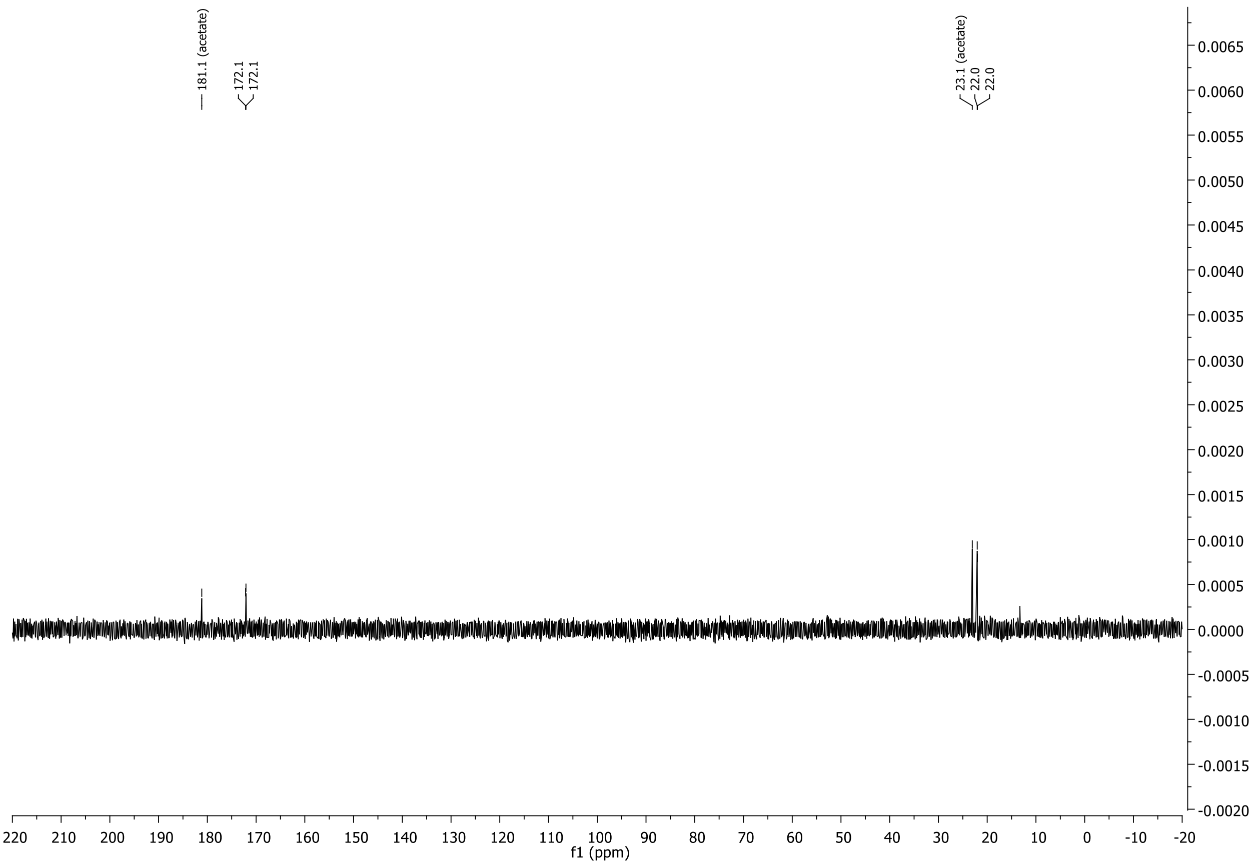

Figure S14. ^{13}C-NMR (125.65 MHz, D_2O) of AcP.

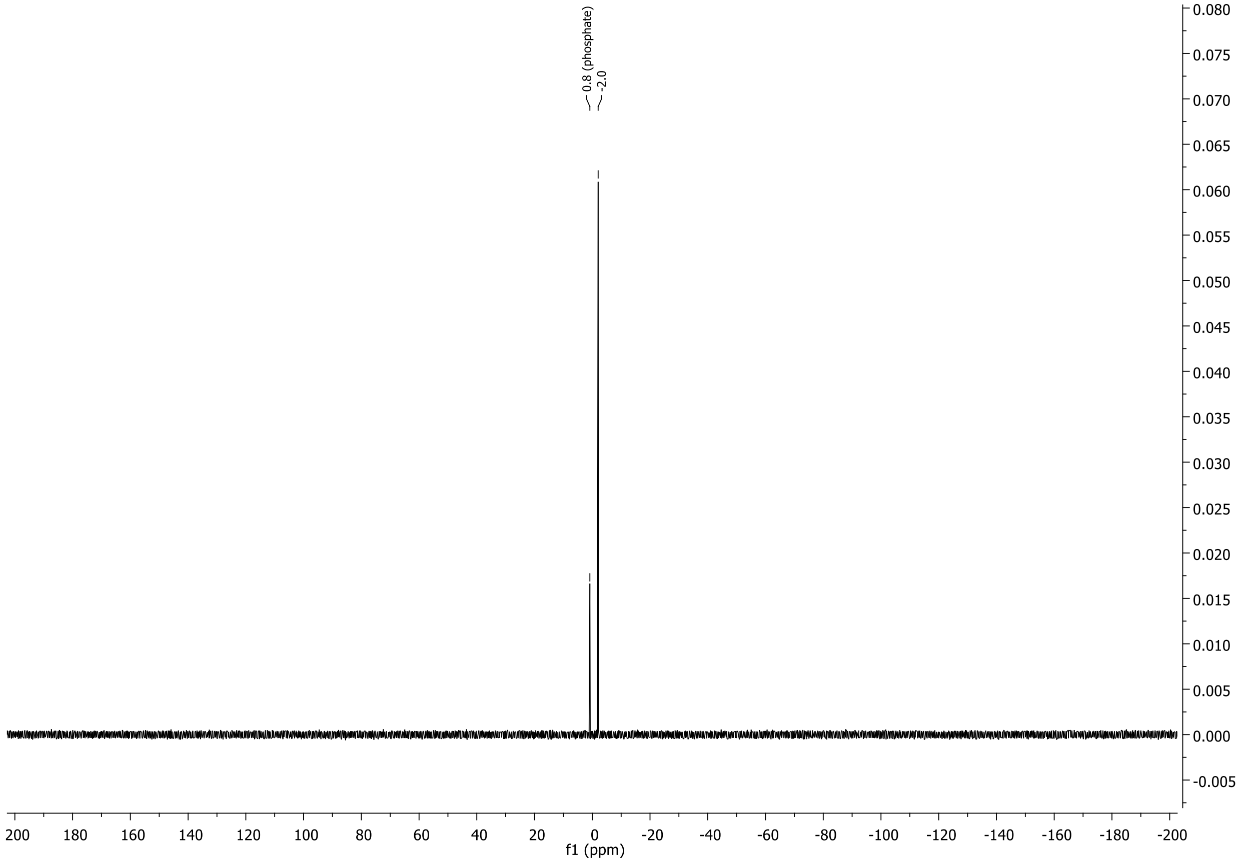

Figure S15. ^{31}P-NMR (202.35 MHz, D_2O) of AcP.

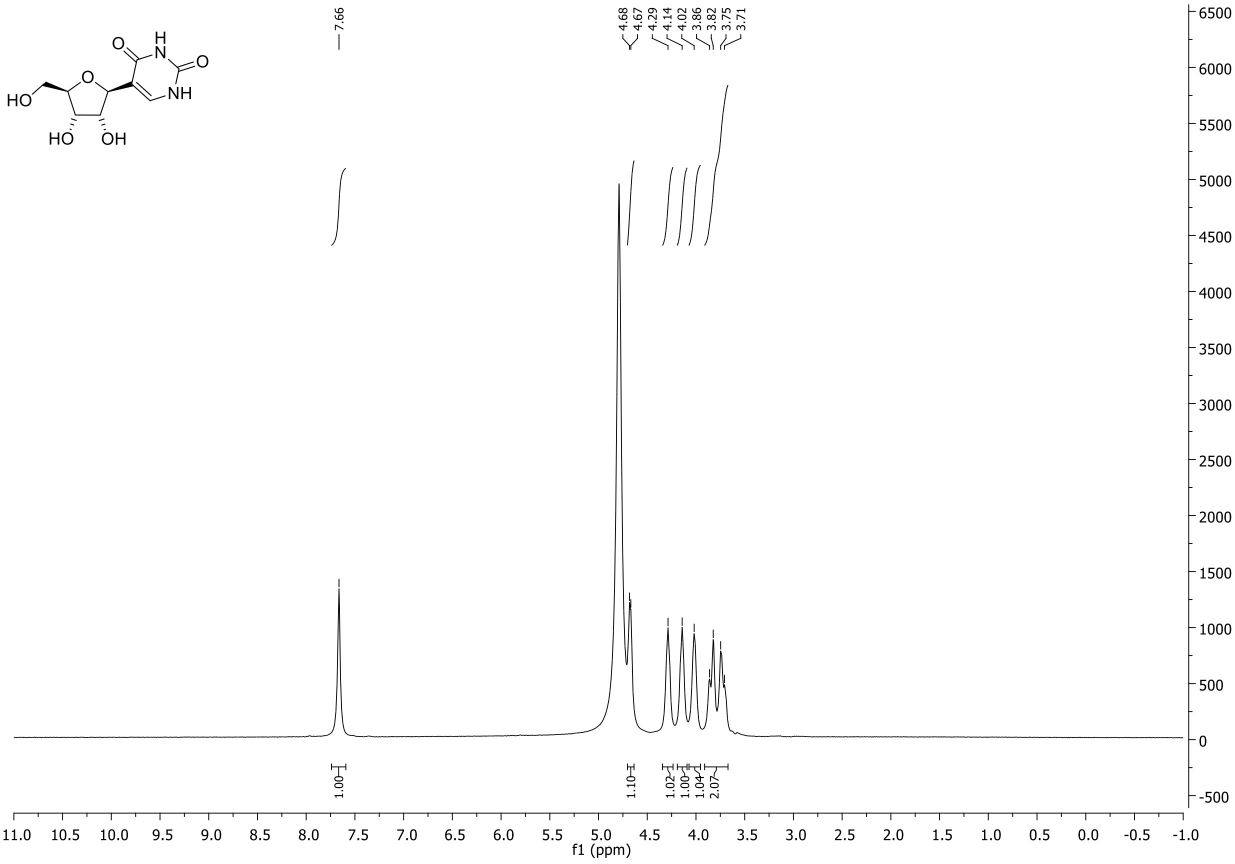

Figure S16. ^{1}H-NMR (300.36 MHz, D_2O) of Ψ (**1**).

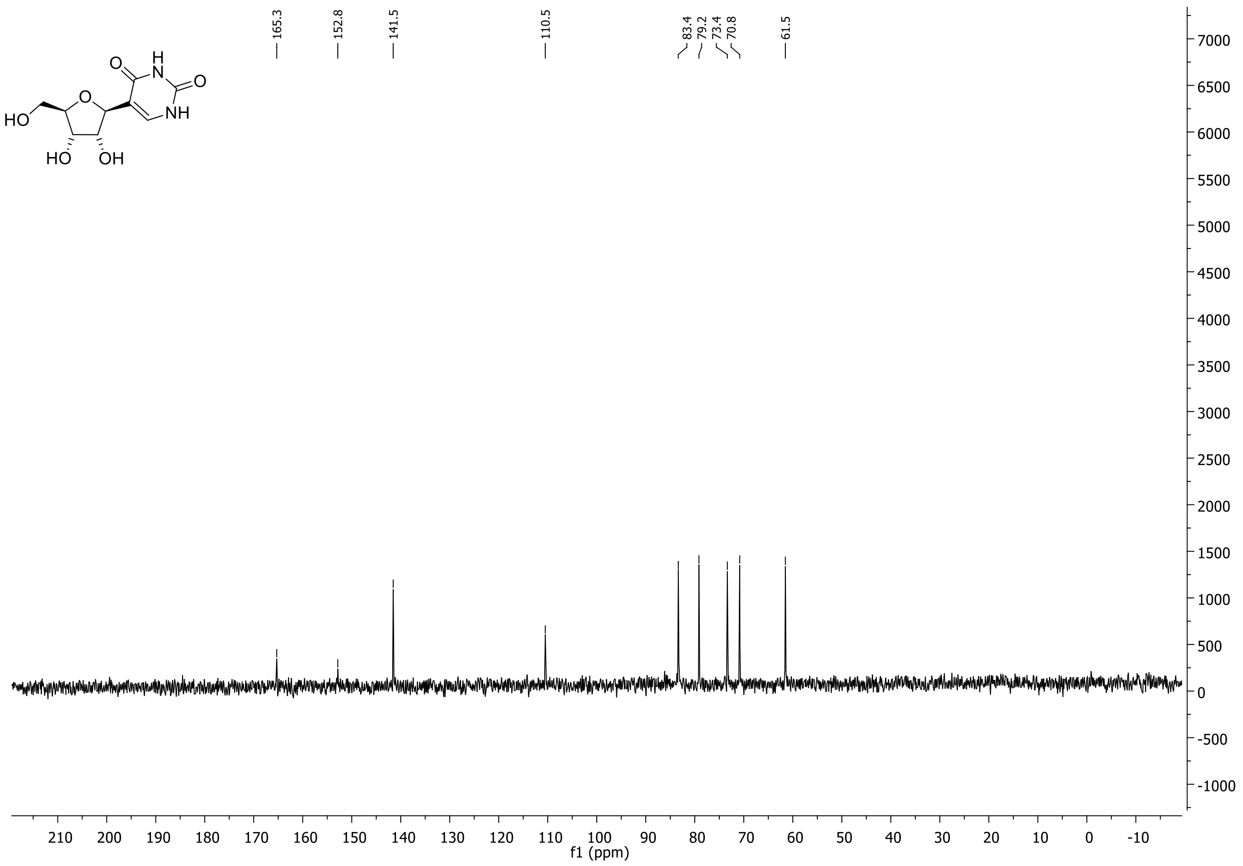

Figure S17. ^{13}C-NMR (75.53 MHz, D_2O) of Ψ (**1**).

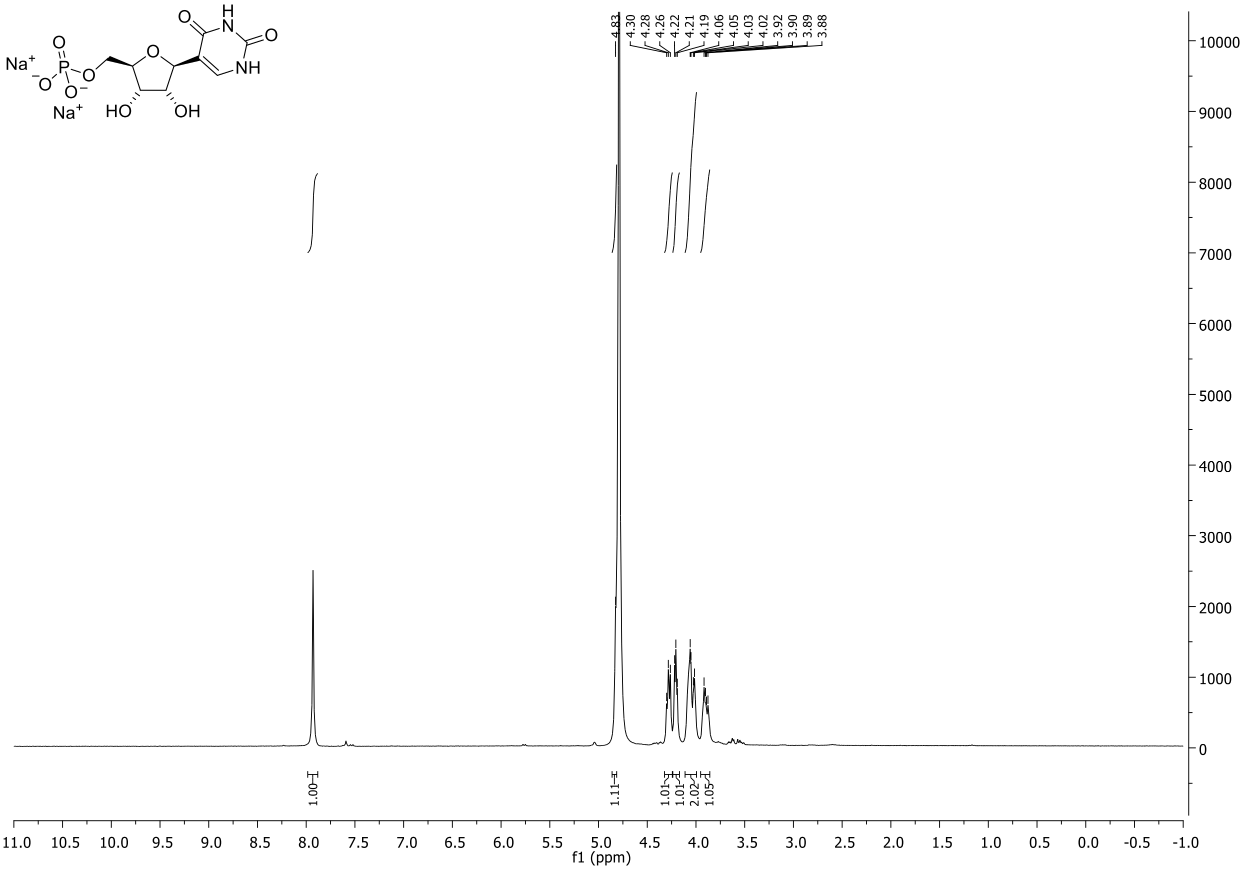

Figure S18. ^{1}H-NMR (300.36 MHz, D_2O) of ΨMP (**1a**).

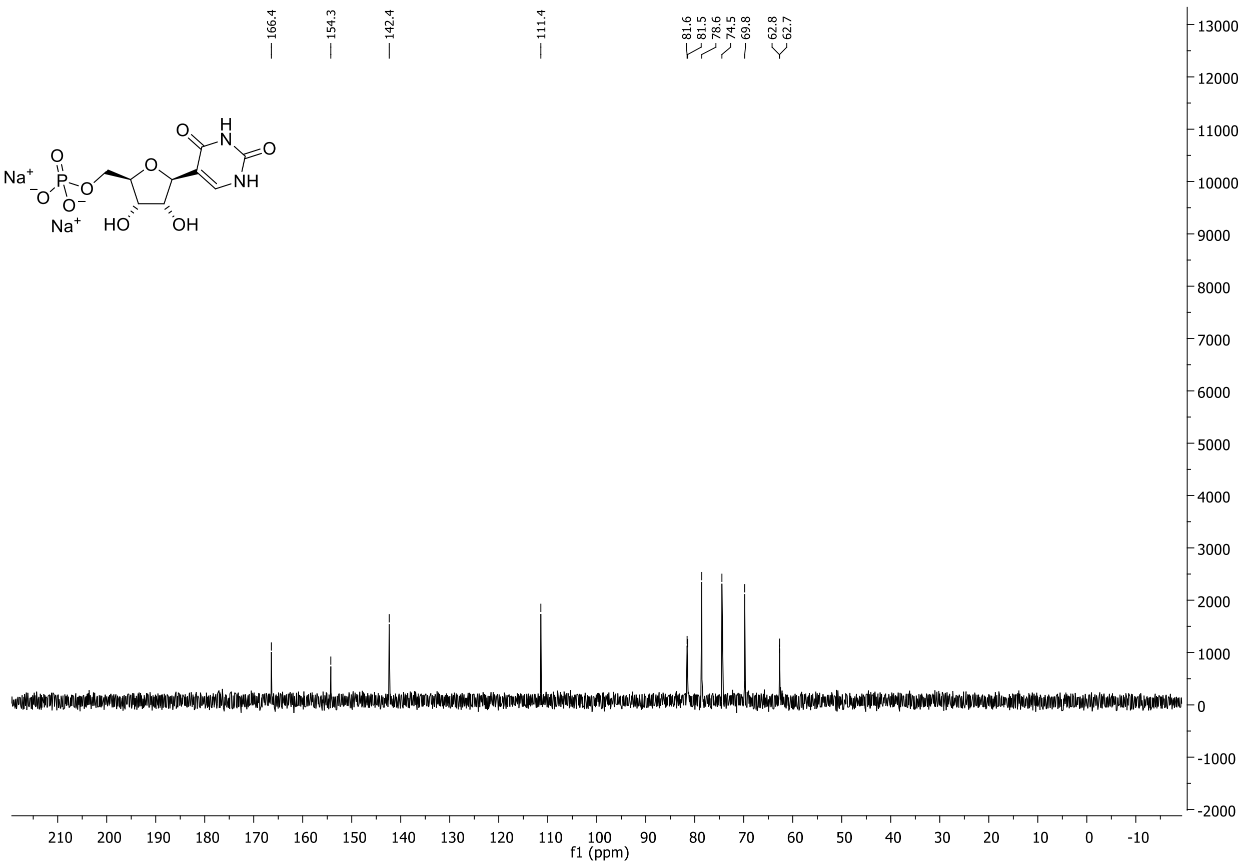

Figure S19. ^{13}C-NMR (75.53 MHz, D_2O) of ΨMP (**1a**).

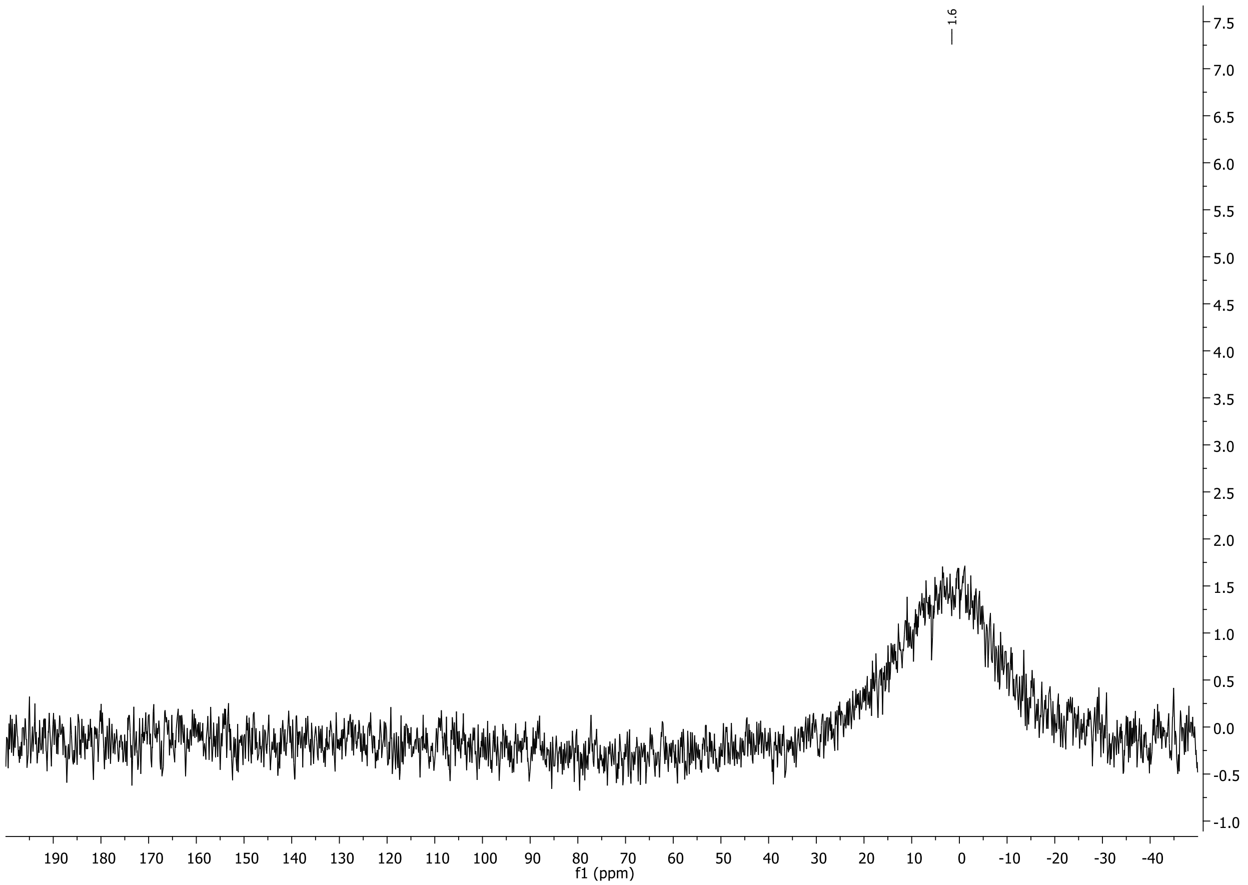

Figure S20. ^{31}P-NMR (202.35 MHz, D_2O) of ΨMP (**1a**). Presence of paramagnetic metal ions (Mn^{2+}) caused line broadening.

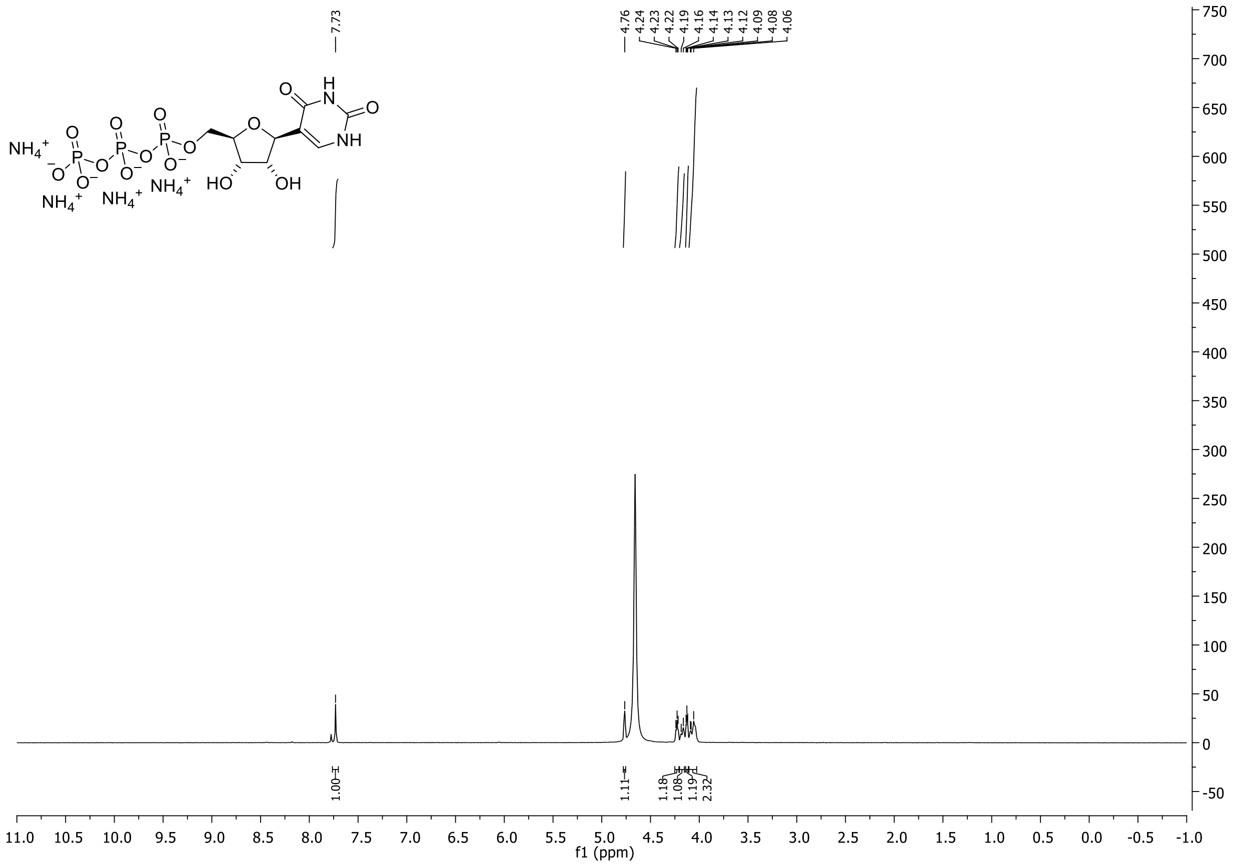

Figure S21. ^{1}H-NMR (499.87 MHz, D_2O) of ΨTP (**1c**).

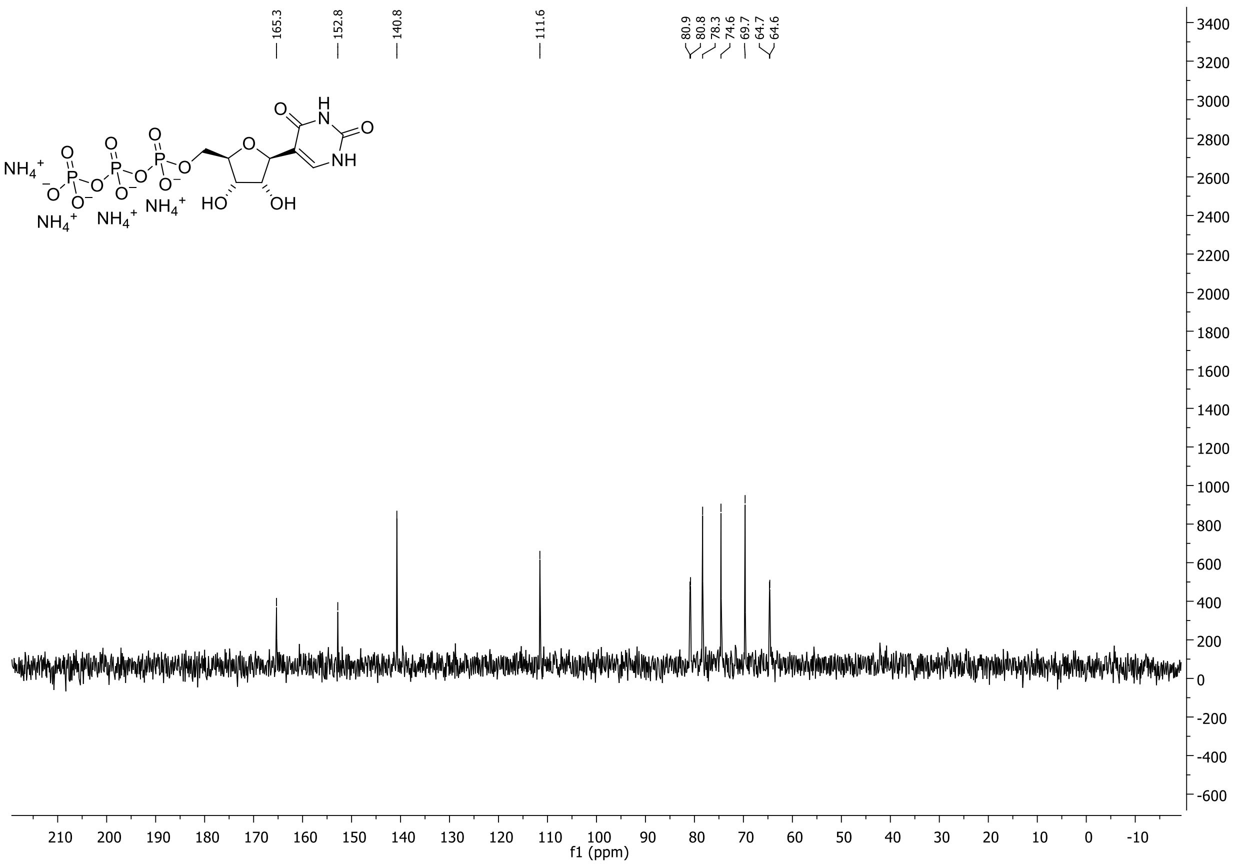

Figure S22. ^{13}C-NMR (125.69 MHz, D_2O) of ΨTP (**1c**).

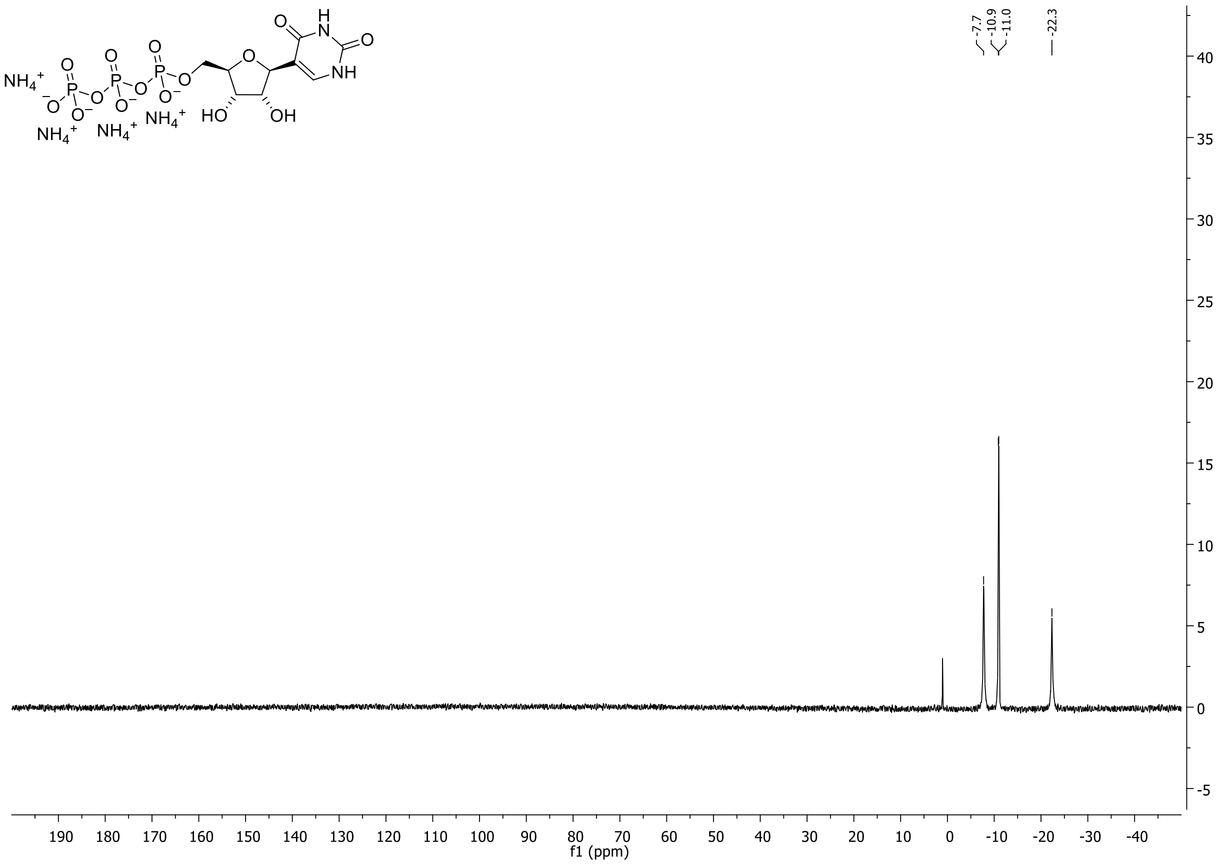

Figure S23. ^{31}P-NMR (202.35 MHz, D_2O) of ΨTP (**1c**).

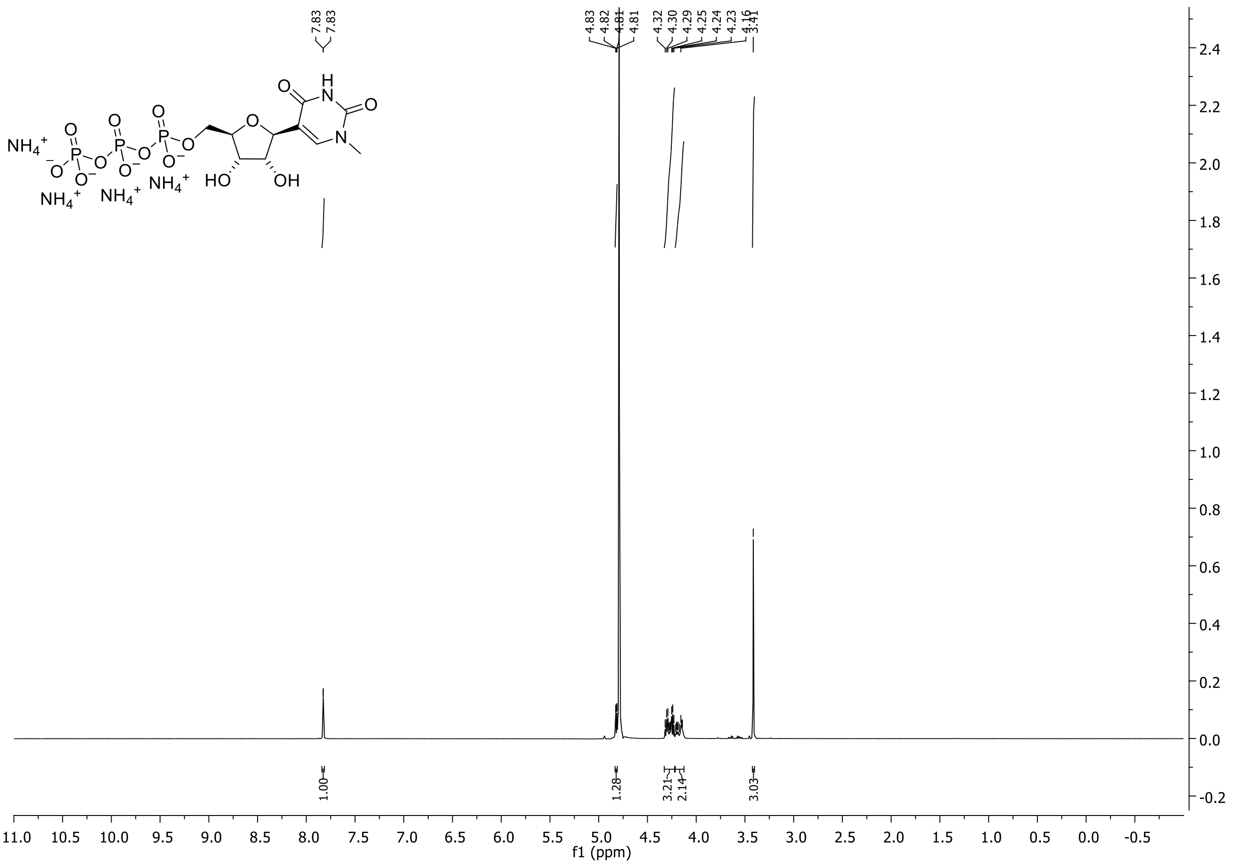

Figure S24. ^{1}H-NMR (399.78 MHz, D_2O) of m^{1}ΨTP (**3c**) produced by chemoenzymatic route as presented in Scheme 2A.

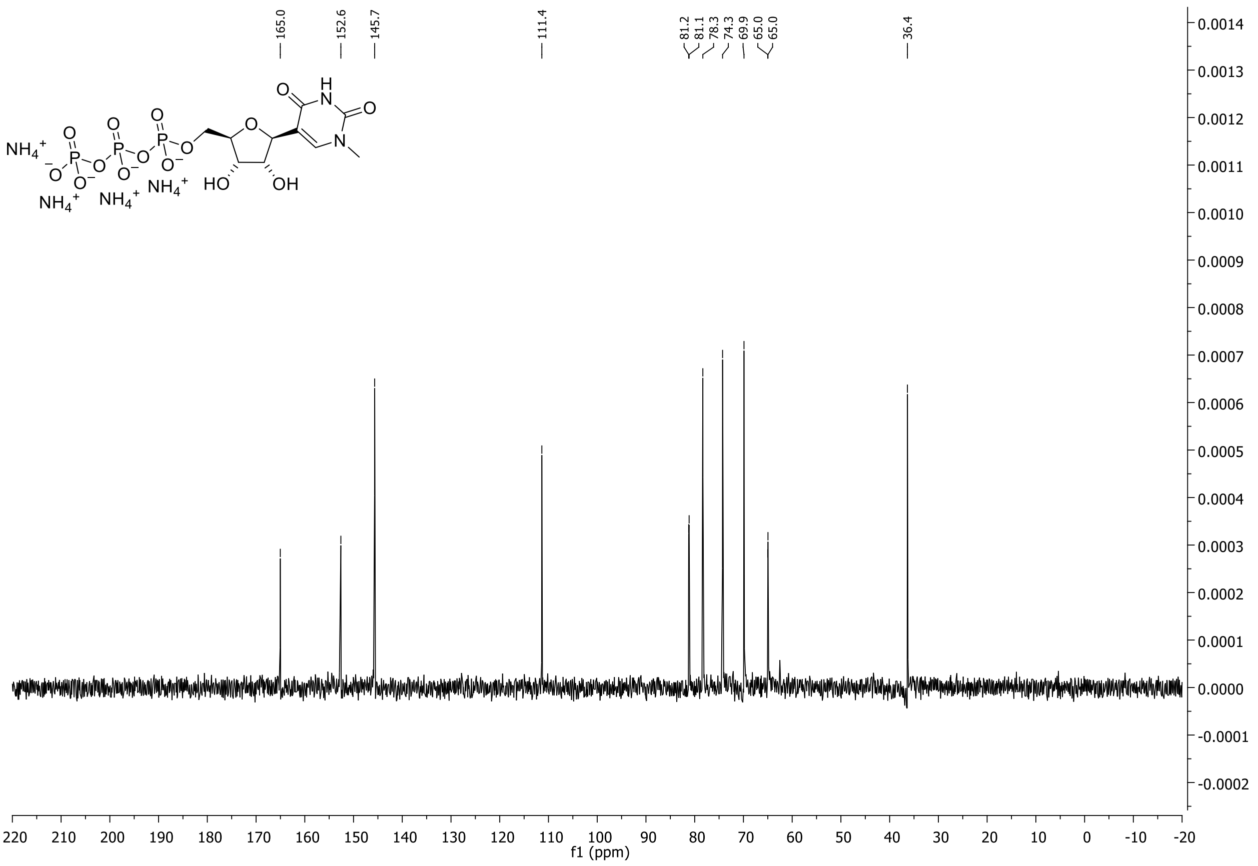

Figure S25. ^{13}C-NMR (100.53 MHz, D_2O) of m^{1}ΨTP (**3c**) produced by chemoenzymatic route as presented in Scheme 2A.

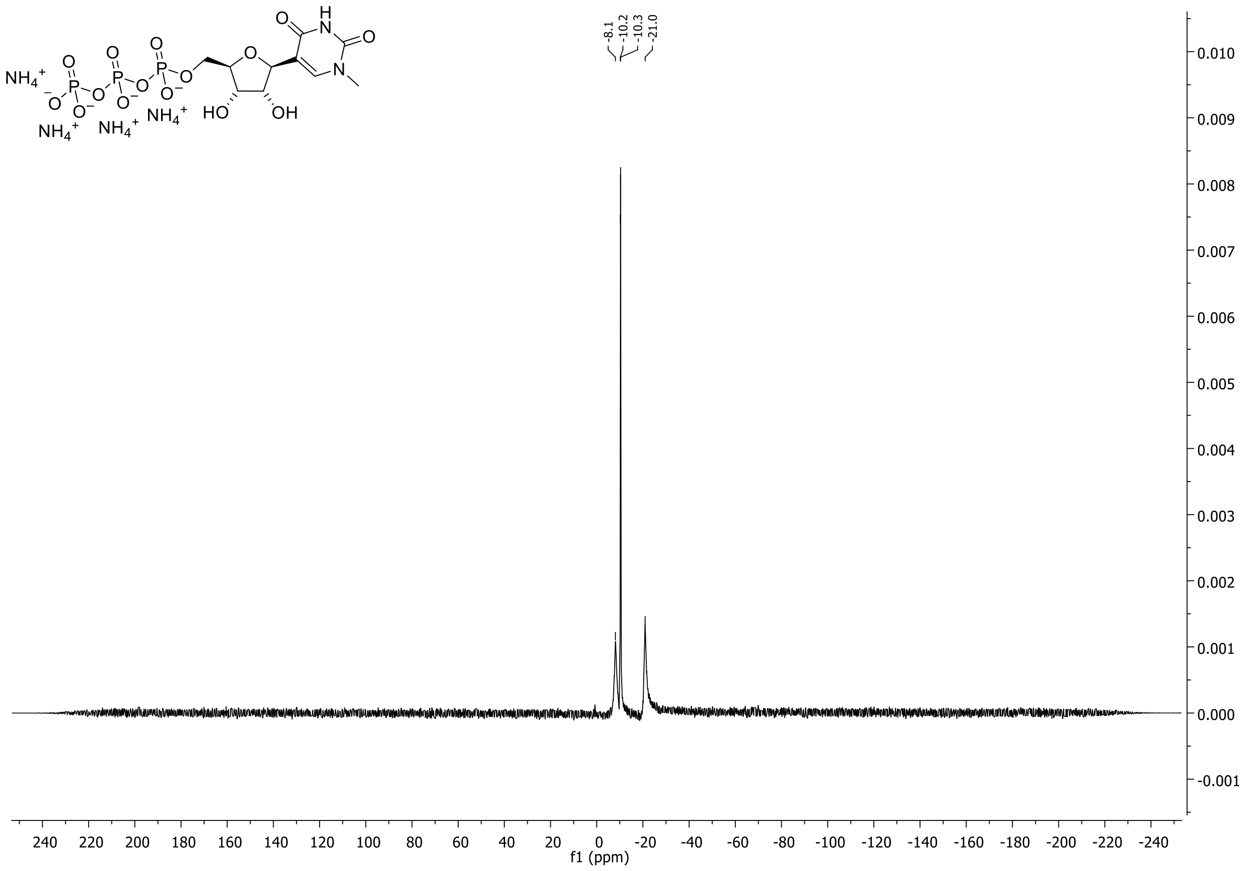

Figure S26. ^{31}P-NMR (161.83 MHz, D_2O) of m^{1}ΨTP (**3c**) produced by chemoenzymatic route as presented in Scheme 2A.

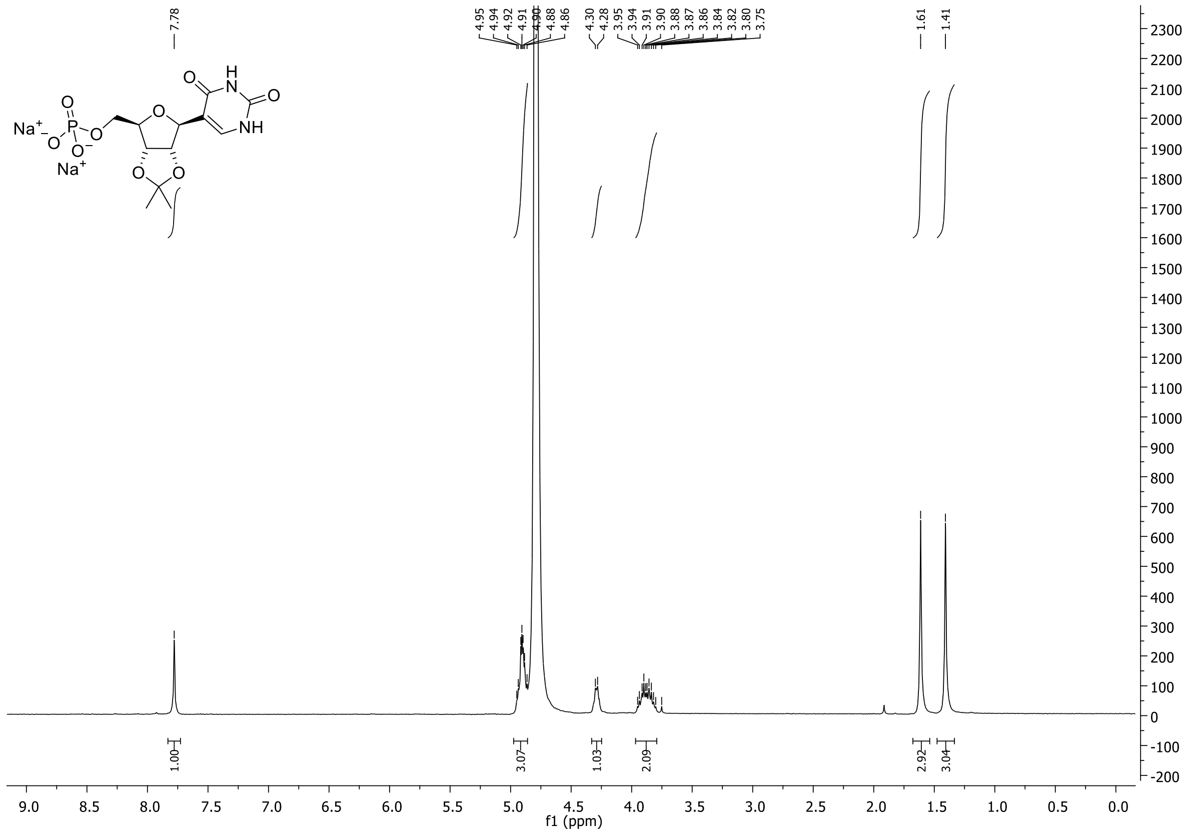

Figure S27. ^{1}H-NMR (300.36 MHz, D_2O) of 2',3'-*O*-isopropylidene-ΨMP (**4**).

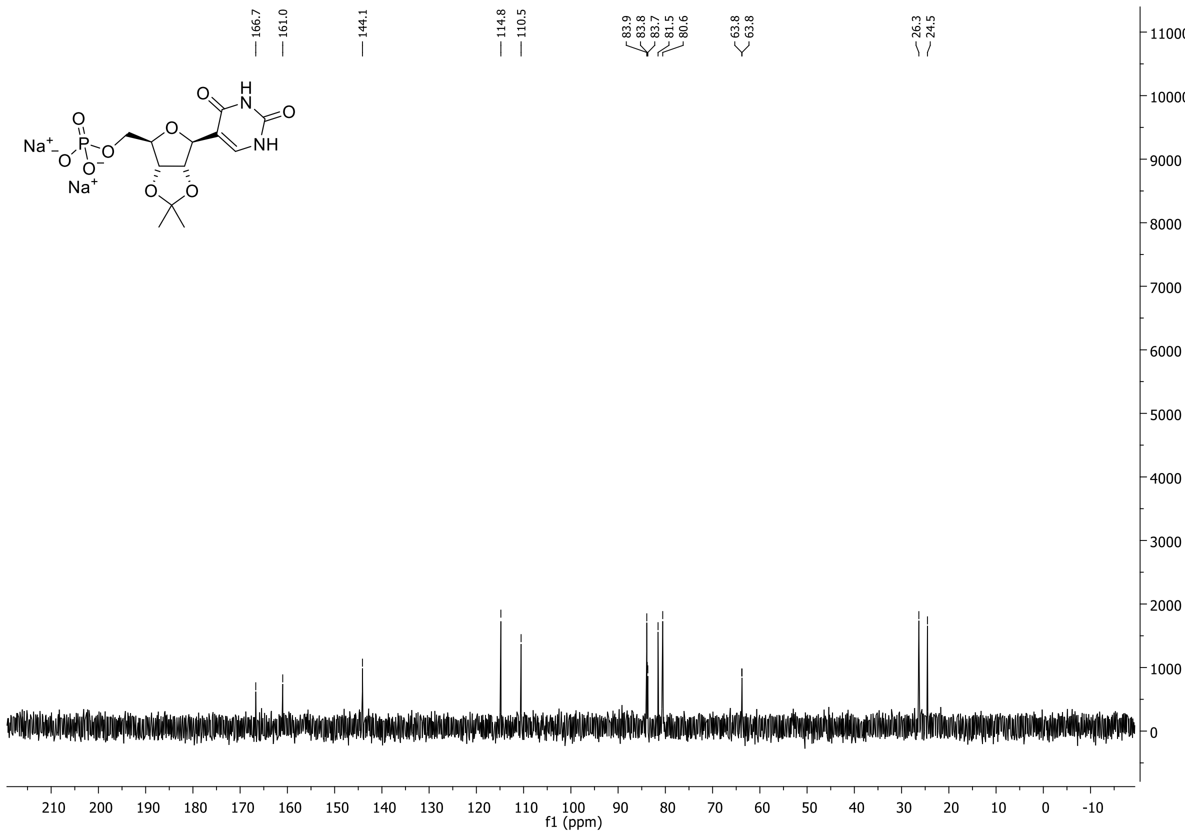

Figure S28. ^{13}C-NMR (75.53 MHz, D_2O) of 2',3'-*O*-isopropylidene-ΨMP (**4**).

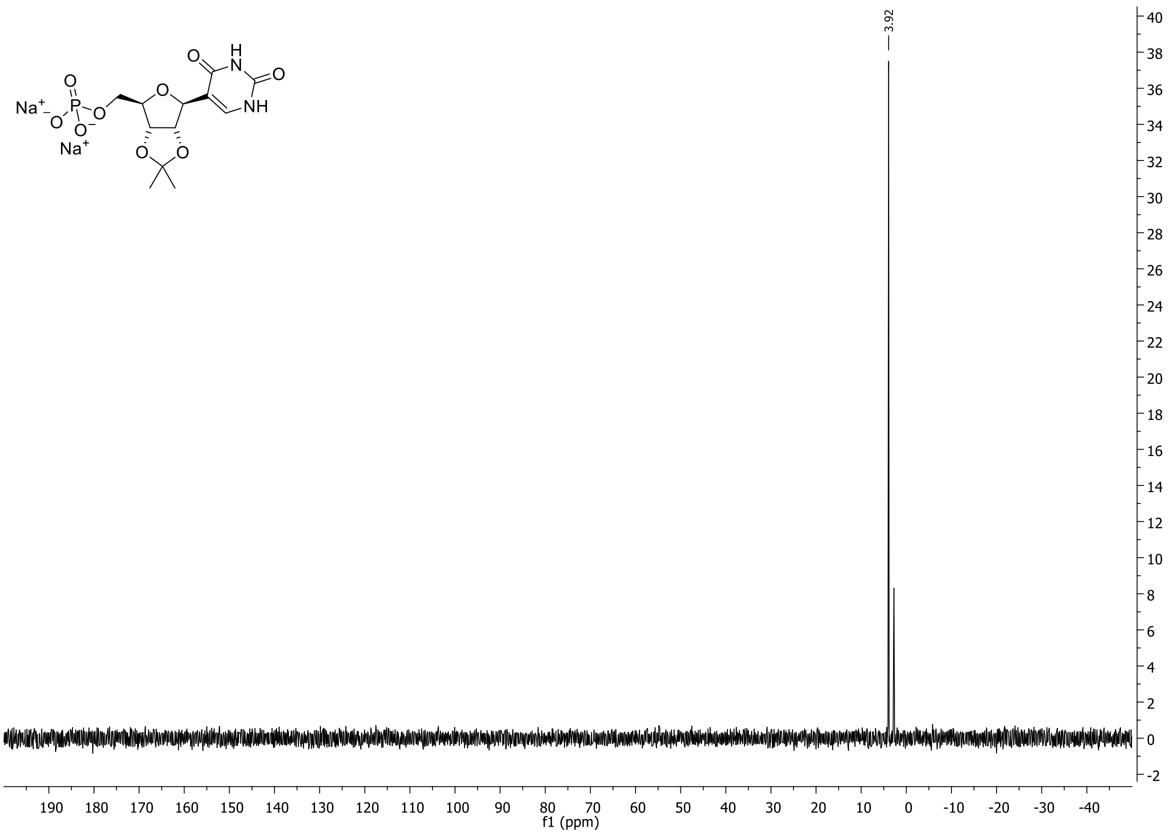

Figure S29. ^{31}P-NMR (202.35 MHz, D_2O) of 2',3'-*O*-isopropylidene-ΨMP (**4**).

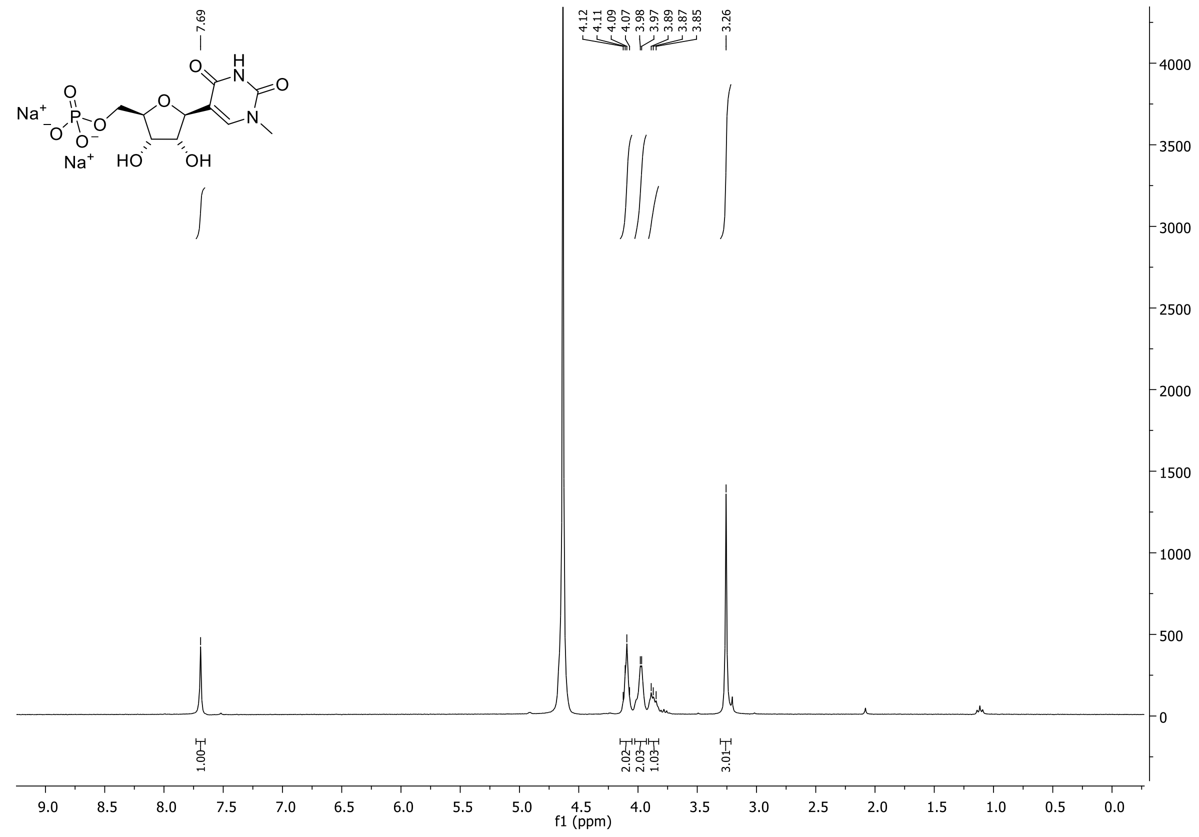

Figure S30. ^{1}H-NMR (300.36 MHz, D_2O) of m**^{1}**ΨMP (**3a**).

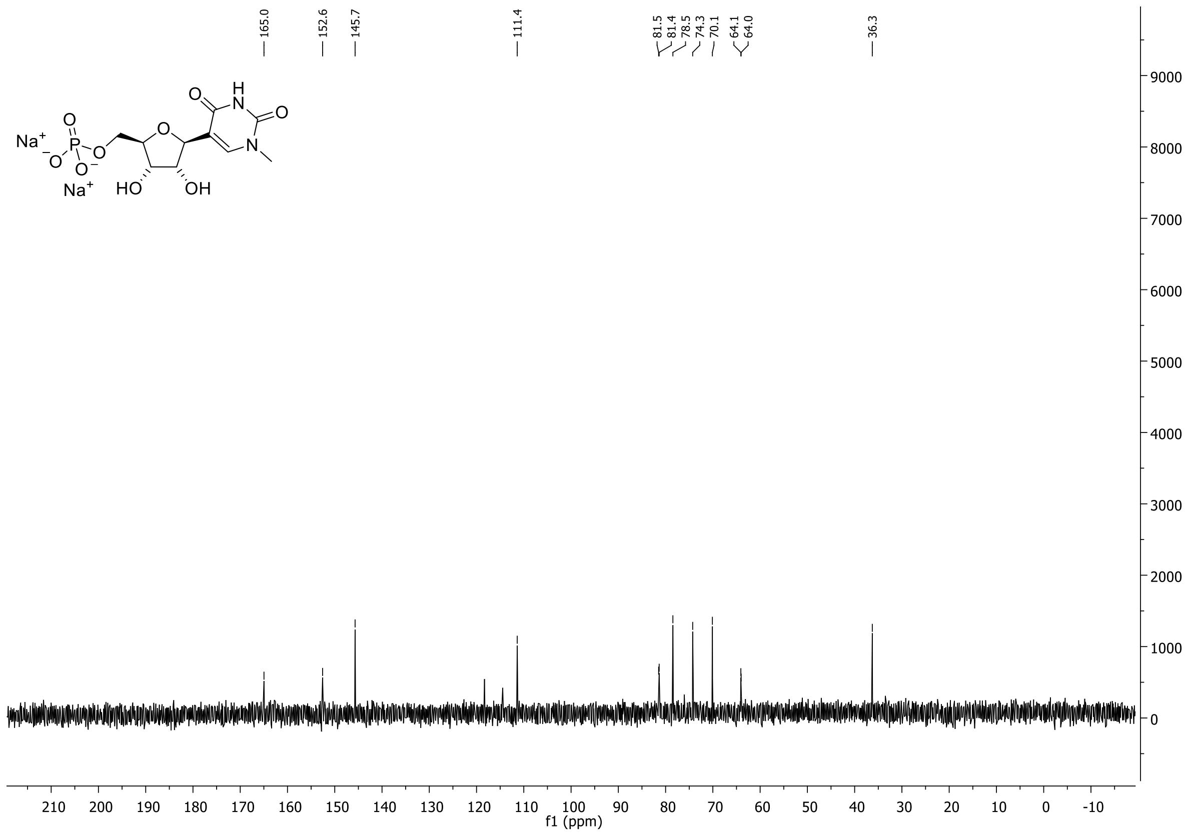

Figure S31. ^{13}C-NMR (75.53 MHz, D_2O) of m^{1}ΨMP (**3a**).

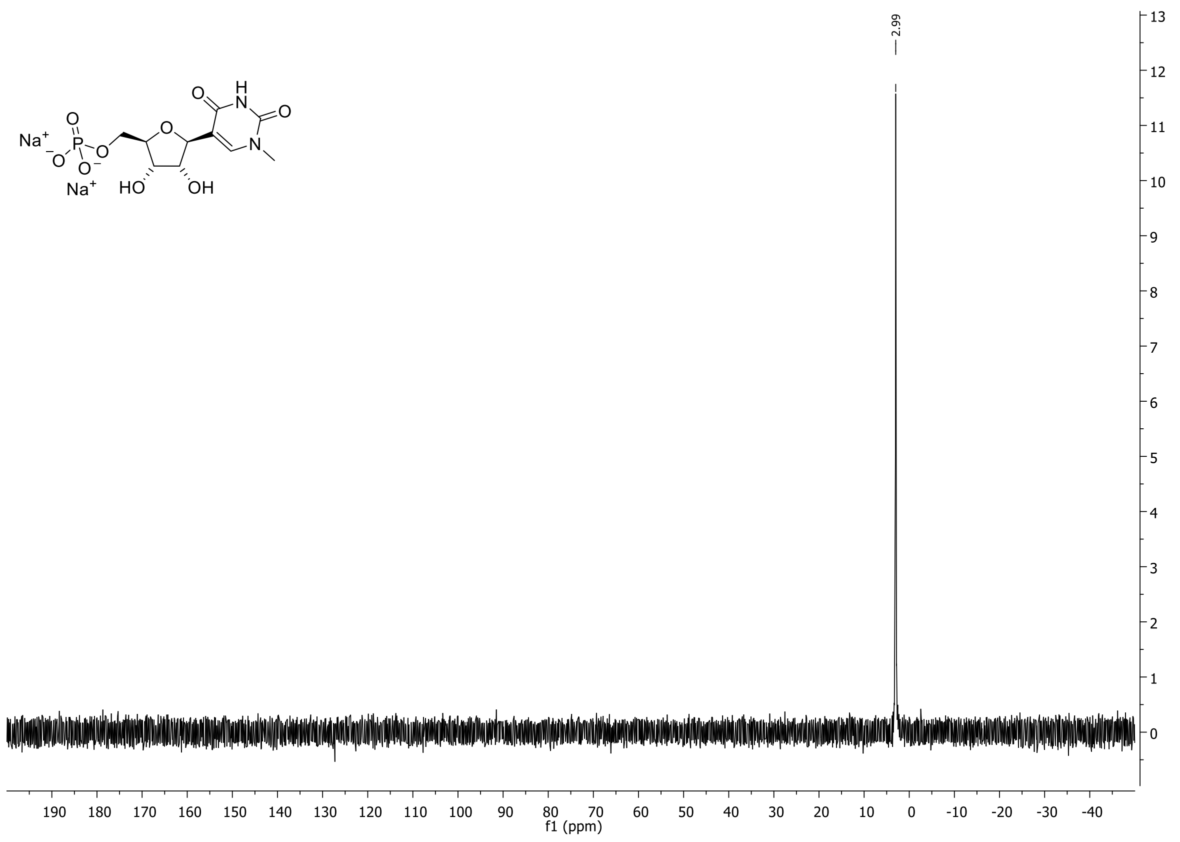

Figure S32. ^{31}P-NMR (202.35 MHz, D_2O) of m^{1}ΨMP (**3a**).

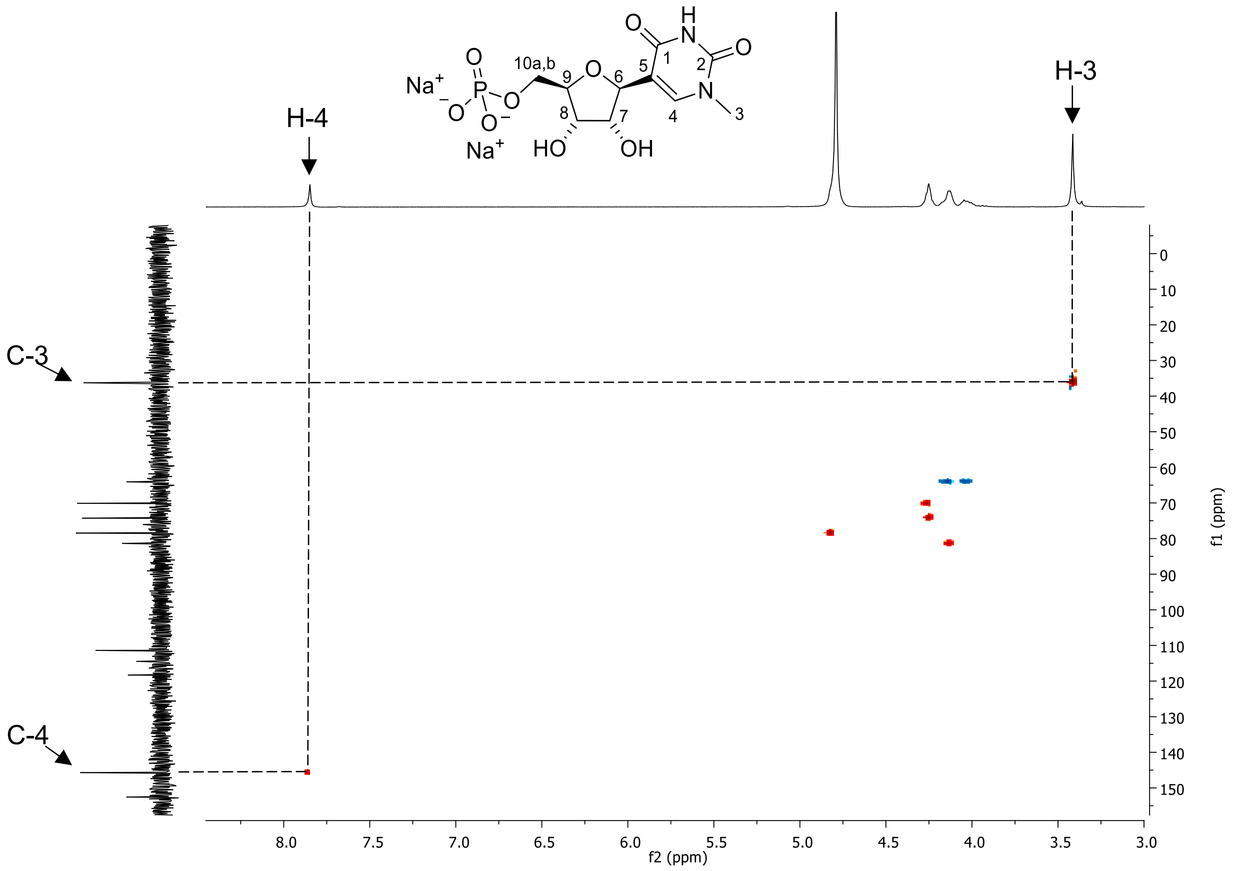

Figure S33. HSQC-NMR (300.36 MHz, 75.53 MHz, D_2O) of m[1]ΨMP (**3a**). Cross peaks are seen between C-3 and H-3 (methyl group on uracil nitrogen), and between C-4 and H-4 (aromatic CH of uracil), confirming the correct assignments of the given hydrogens and carbons, respectively.

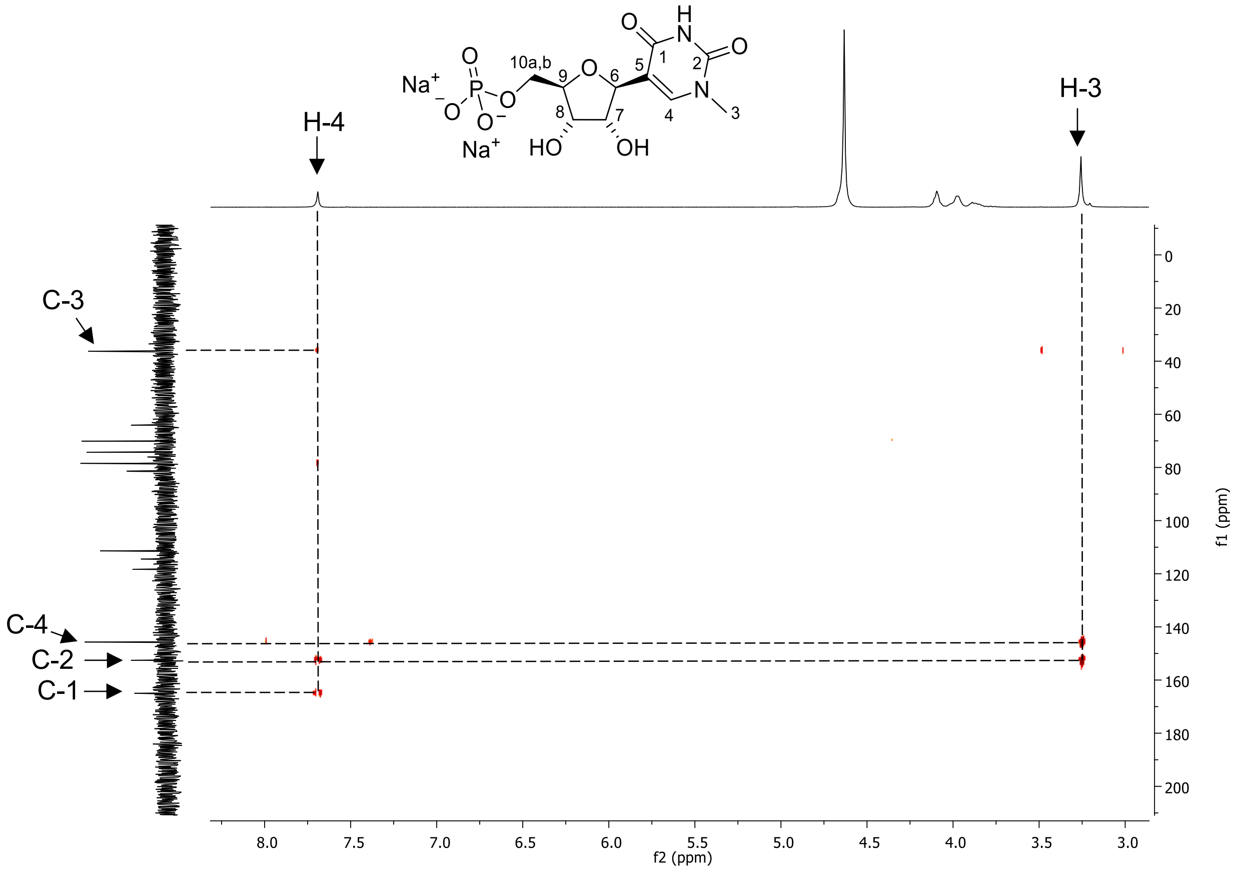

Figure S34. HMBC-NMR (300.36 MHz, 75.53 MHz, D_2O) of m[1]ΨMP (**3a**). Cross peaks are seen between C-1 (carbonyl-C of uracil ring) and H-4 (aromatic proton of uracil ring), C-2 (carbonyl-C between the two nitrogen atoms of uracil ring) and H-4 (aromatic proton of uracil ring), C-2 and H-3 (methyl group on the nitrogen between carbonyl C-2 and aromatic C-4), C-3 (methyl group on uracil nitrogen) and H-4 and between C-4 (aromatic carbon) and H-3, which unequivocally confirms the correct structure of m[1]ΨMP (**3a**).

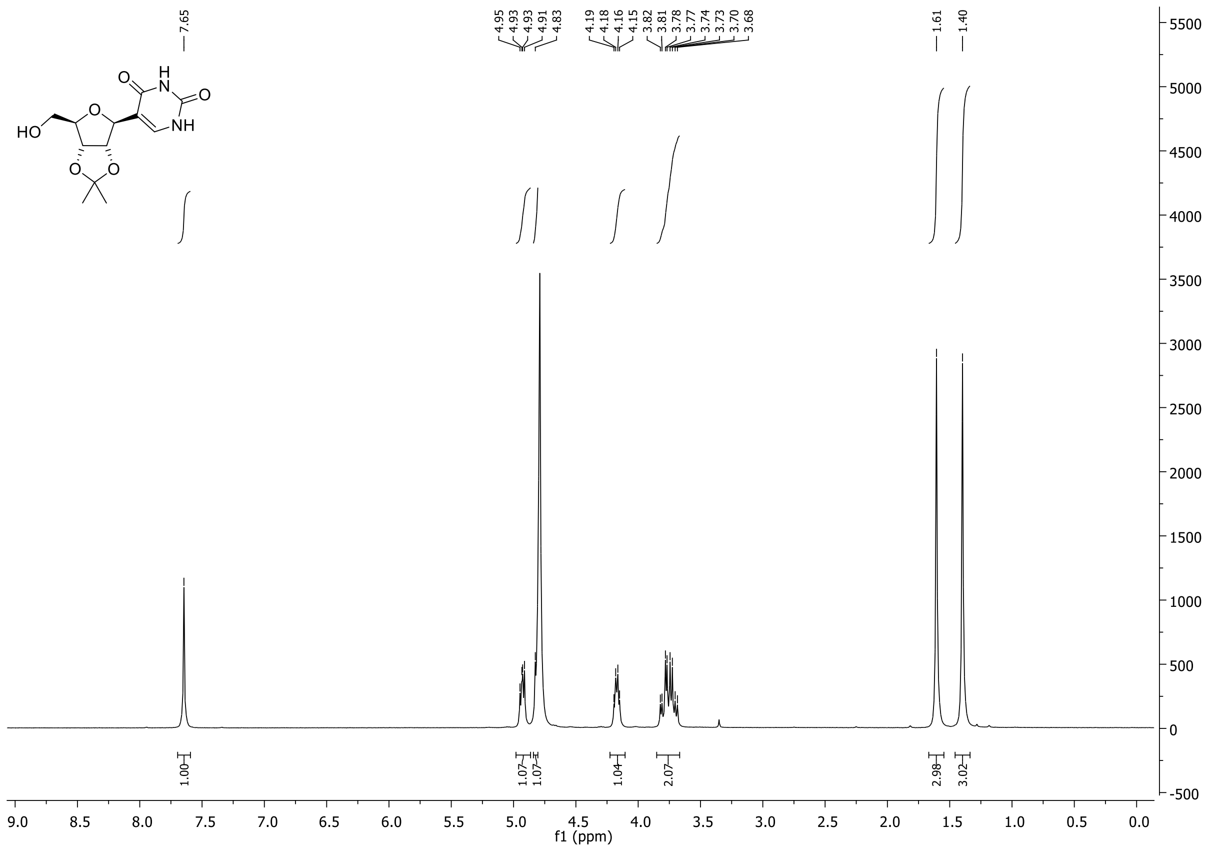

Figure S35. ^{1}H-NMR (300.36 MHz, D_2O) of 2’,3’-*O*-isopropylidene-Ψ (**5**).

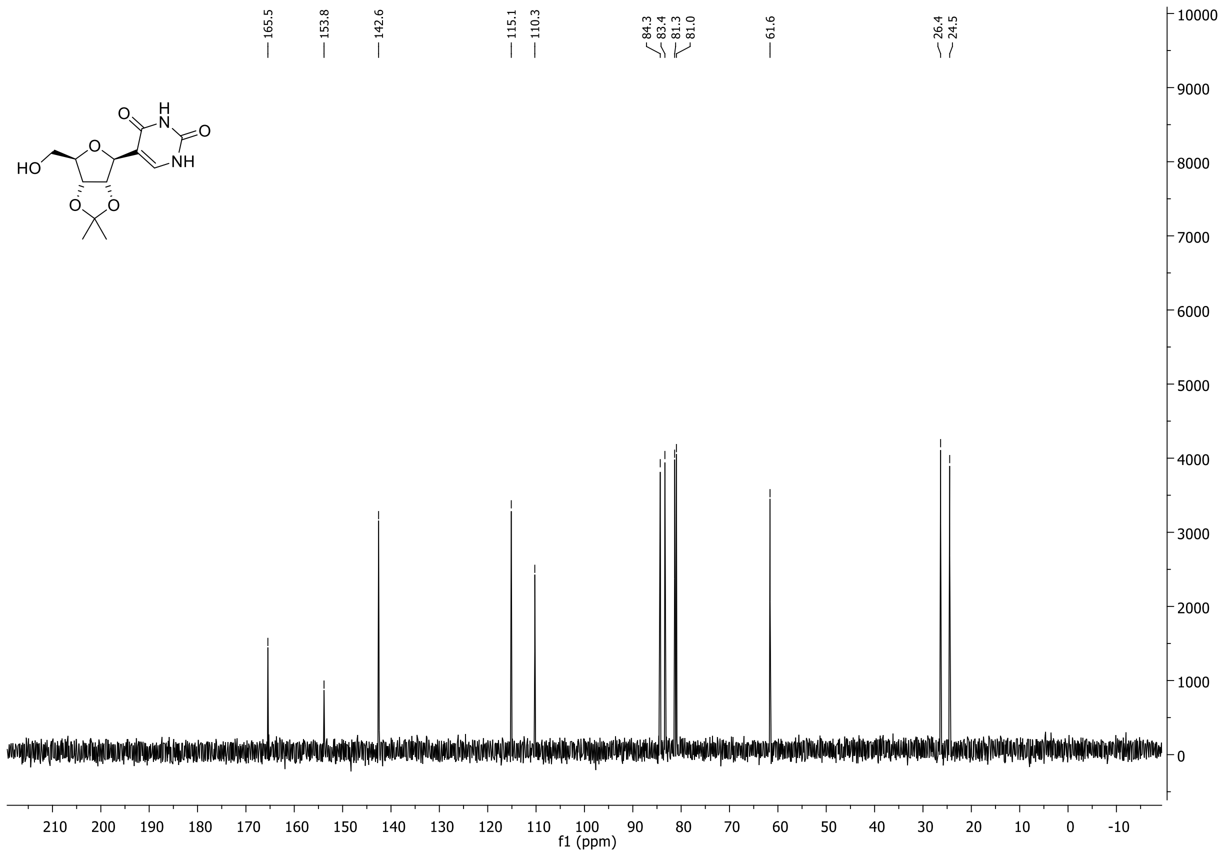

Figure S36. ^{13}C-NMR (75.53 MHz, D_2O) of 2',3'-*O*-isopropylidene-Ψ (**5**).

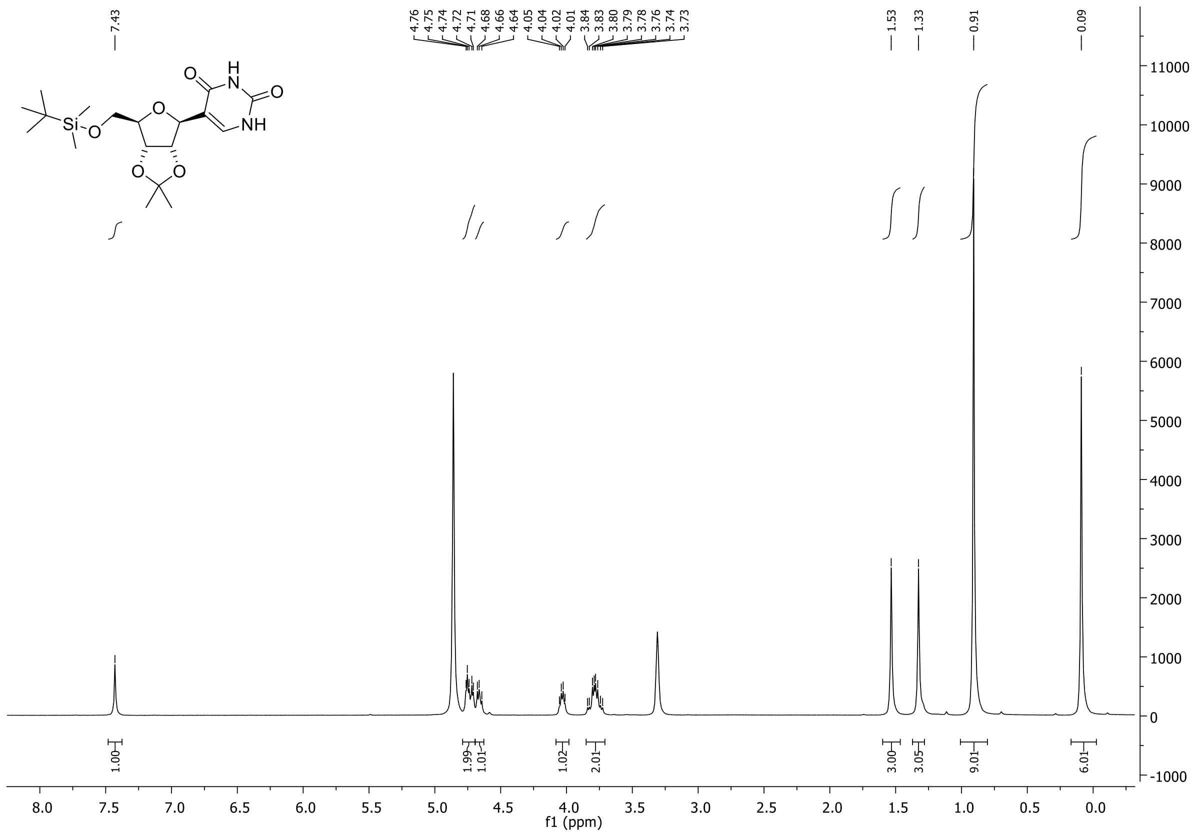

Figure S37. ^{1}H-NMR (300.36 MHz, CD_3OD) of 2’,3’-*O*-isopropylidene-5’-*O*-(*tert*-butyldimethylsilyl)-Ψ (**6**).

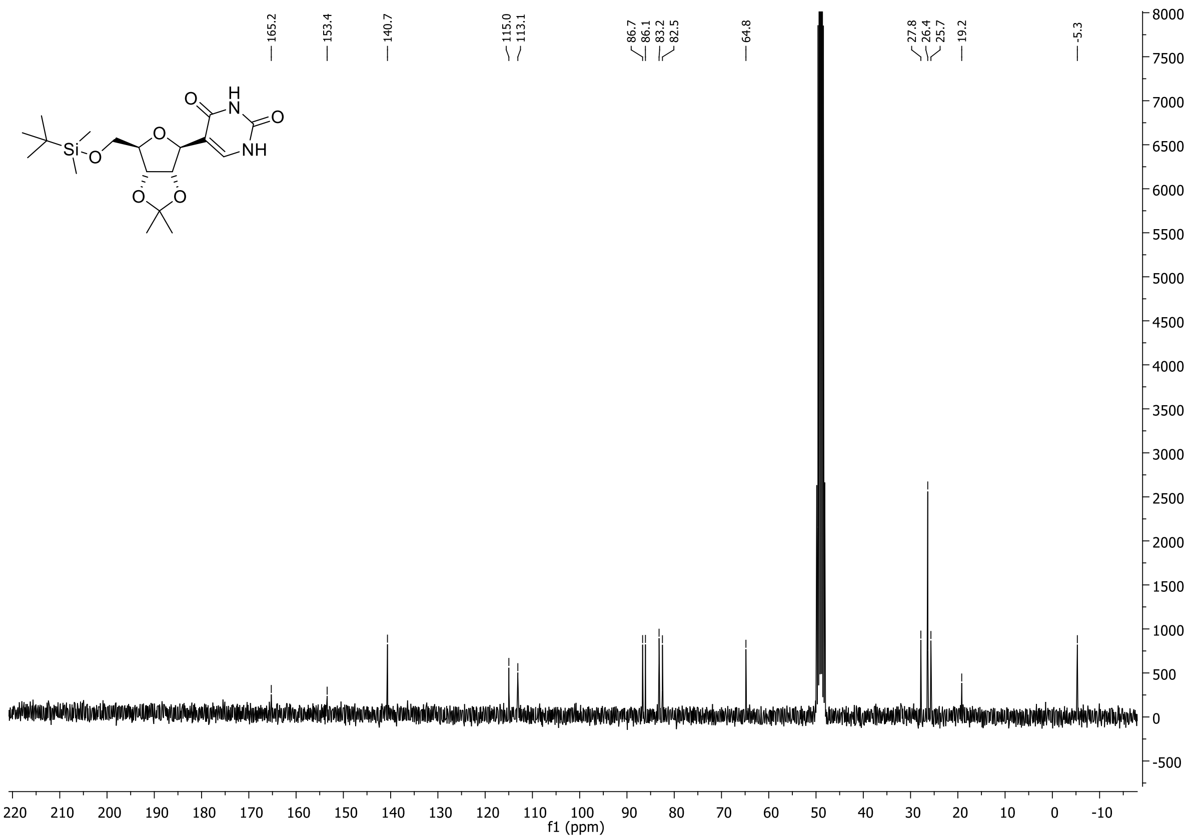

Figure S38. ^{13}C-NMR (75.53 MHz, CD_3OD) of 2',3'-*O*-isopropylidene-5'-*O*-(*tert*-butyldimethylsilyl)-Ψ (**6**).

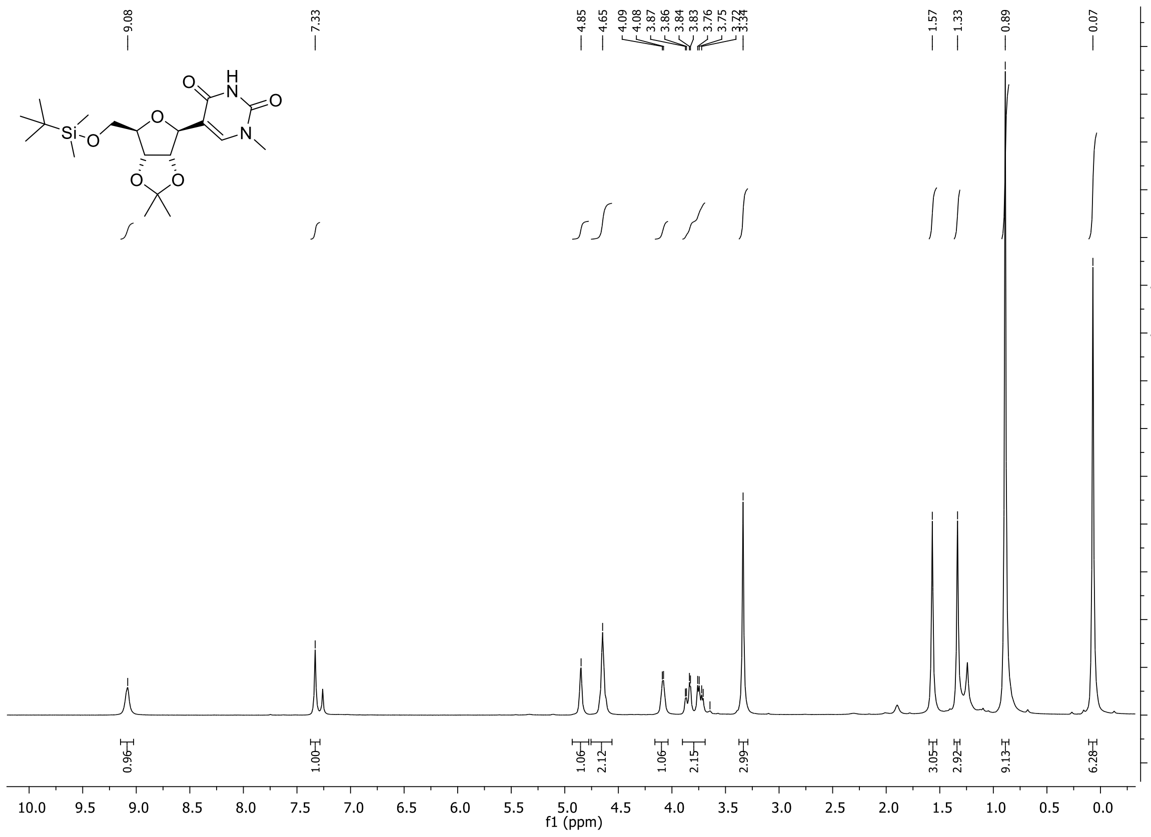

Figure S39. ^{1}H-NMR (300.36 MHz, $CDCl_3$) of *N*^{1}-methyl-2',3'-*O*-isopropylidene-5'-*O*-(*tert*-butyldimethylsilyl)-Ψ (**7**).

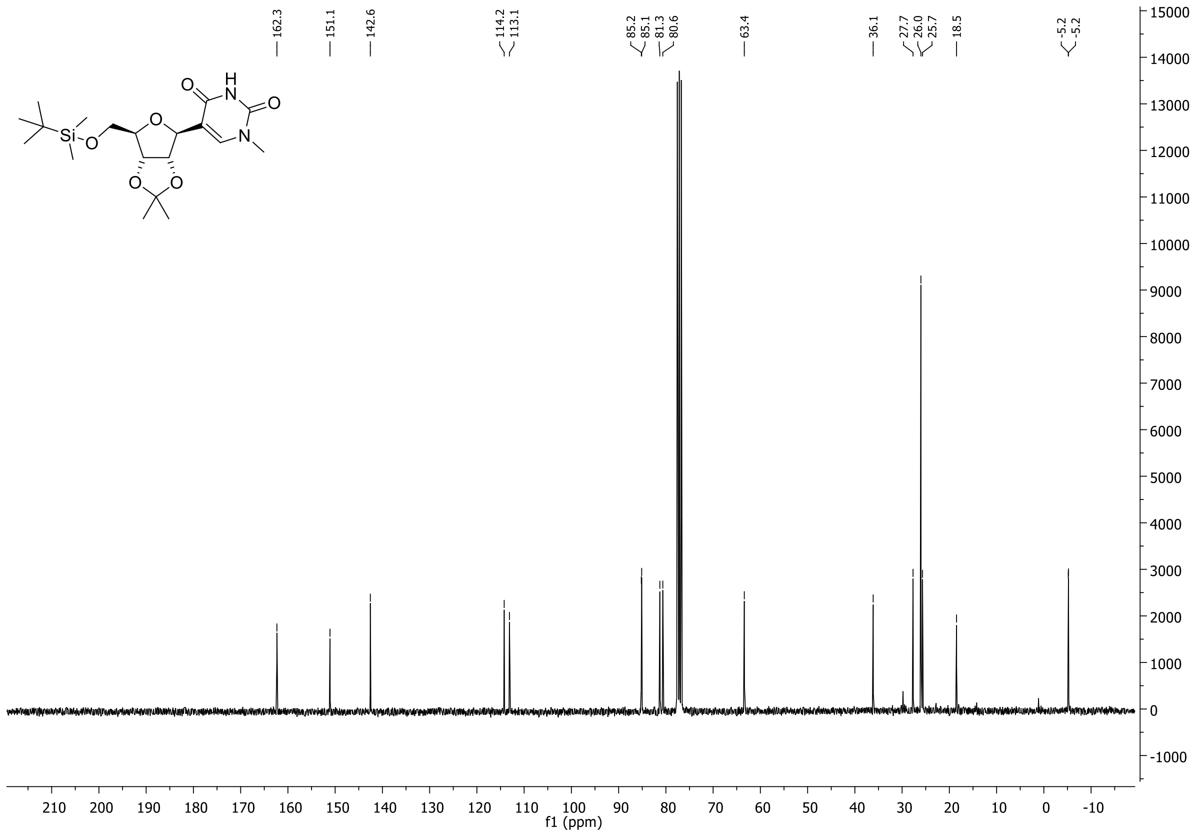

Figure S40. ^{13}C-NMR (75.53 MHz, $CDCl_3$) of *N*[1]-methyl-2',3'-*O*-isopropylidene-5'-*O*-(*tert*-butyldimethylsilyl)-Ψ (**7**).

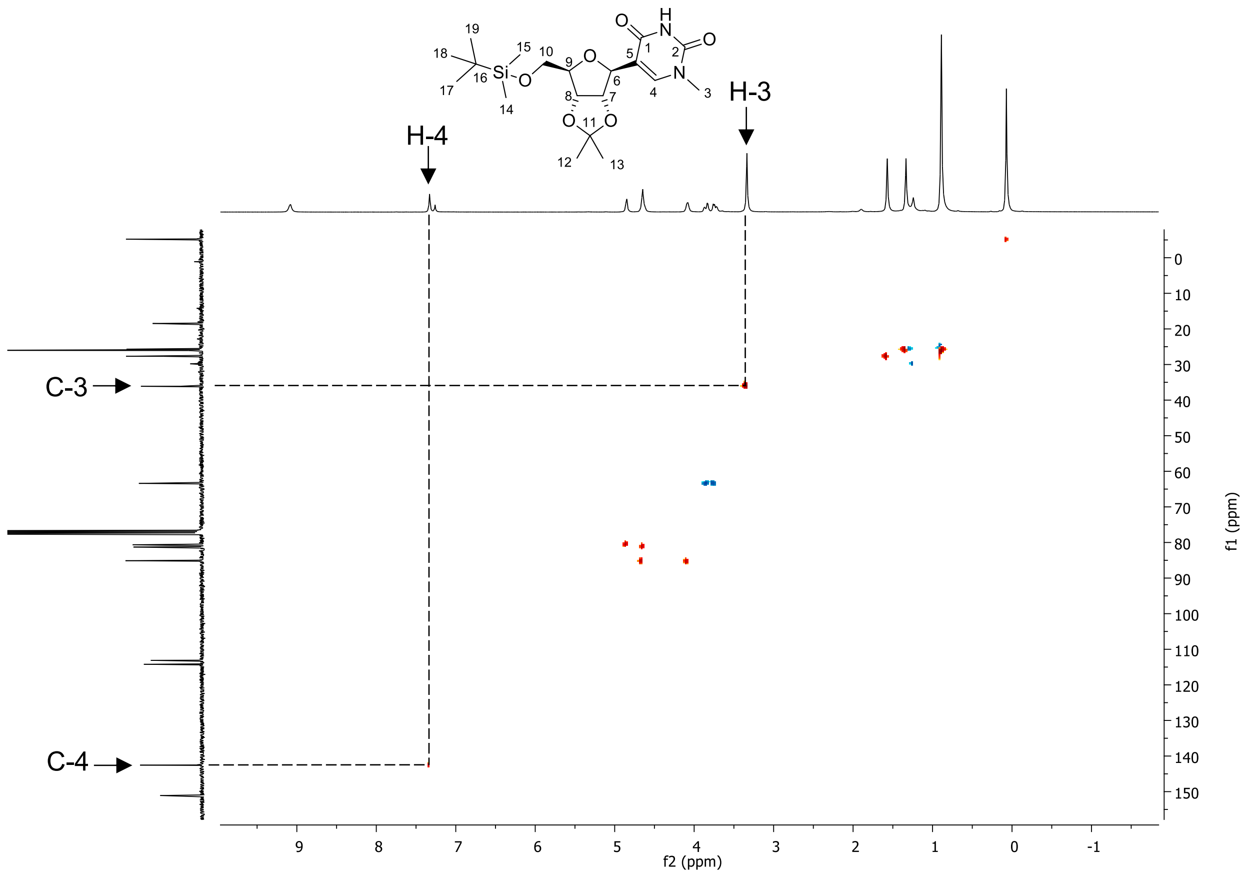

Figure S41. HSQC-NMR (300.36 MHz, 75.53 MHz, D_2O) of *N*[1]-methyl-2',3'-*O*-isopropylidene-5'-*O*-(*tert*-butyldimethylsilyl)-Ψ (**7**). Cross peaks are seen between C-3 and H-3 (methyl group on uracil nitrogen), and between C-4 and H-4 (aromatic CH of uracil), confirming the correct assignments of the given hydrogens and carbons, respectively.

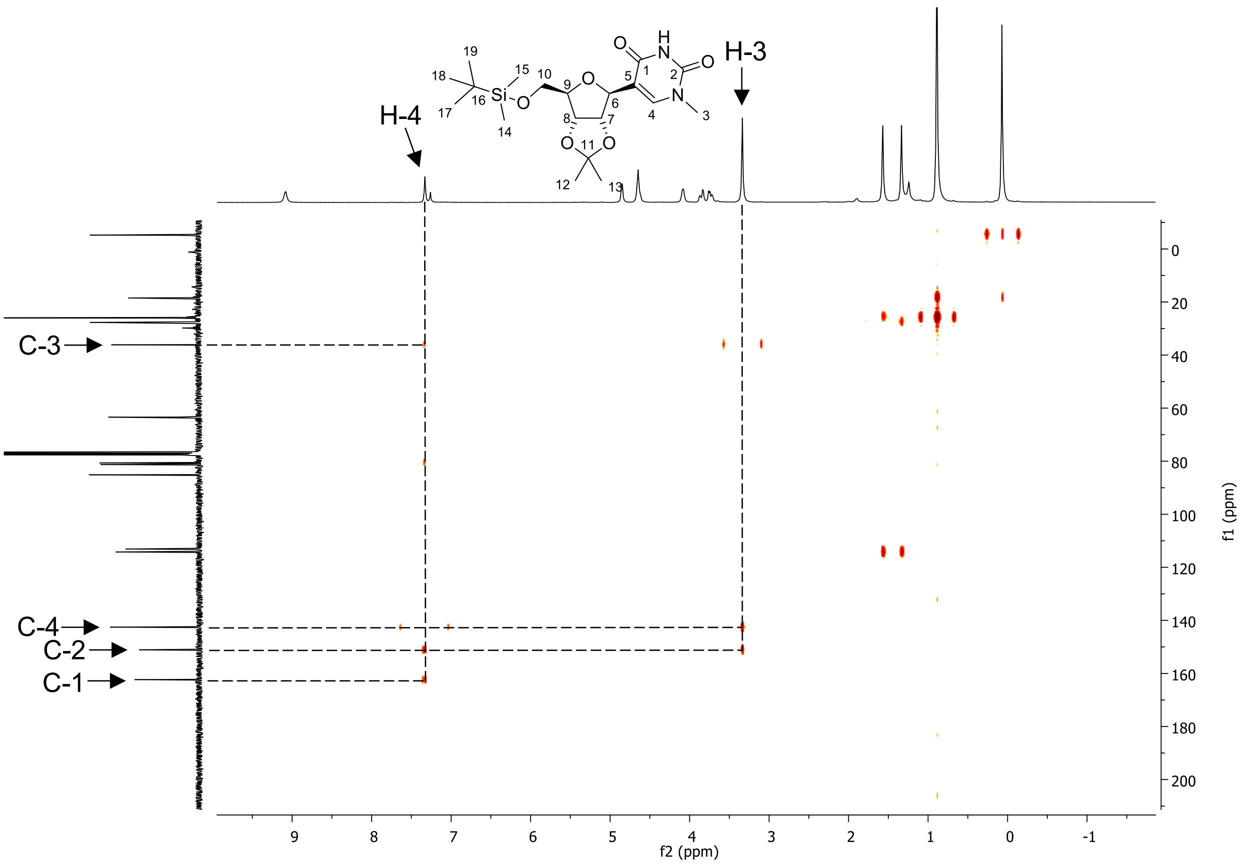

Figure S42. HMBC-NMR (300.36 MHz, 75.53 MHz, D_2O) of *N*[1]-methyl-2',3'-*O*-isopropylidene-5'-*O*-(*tert*-butyldimethylsilyl)-Ψ (**7**). Cross peaks are seen between C-1 (carbonyl-C of uracil ring) and H-4 (aromatic proton of uracil ring), C-2 (carbonyl-C between the two nitrogen atoms of uracil ring) and H-4 (aromatic proton of uracil ring), C-2 and H-3 (methyl group on the nitrogen between carbonyl C-2 and aromatic C-4), C-3 (methyl group on uracil nitrogen) and H-4 and between C-4 (aromatic carbon) and H-3, which unequivocally confirms the correct structure of *N*[1]-methyl-2',3'-*O*-isopropylidene-5'-*O*-(*tert*-butyldimethylsilyl)-Ψ (**7**).

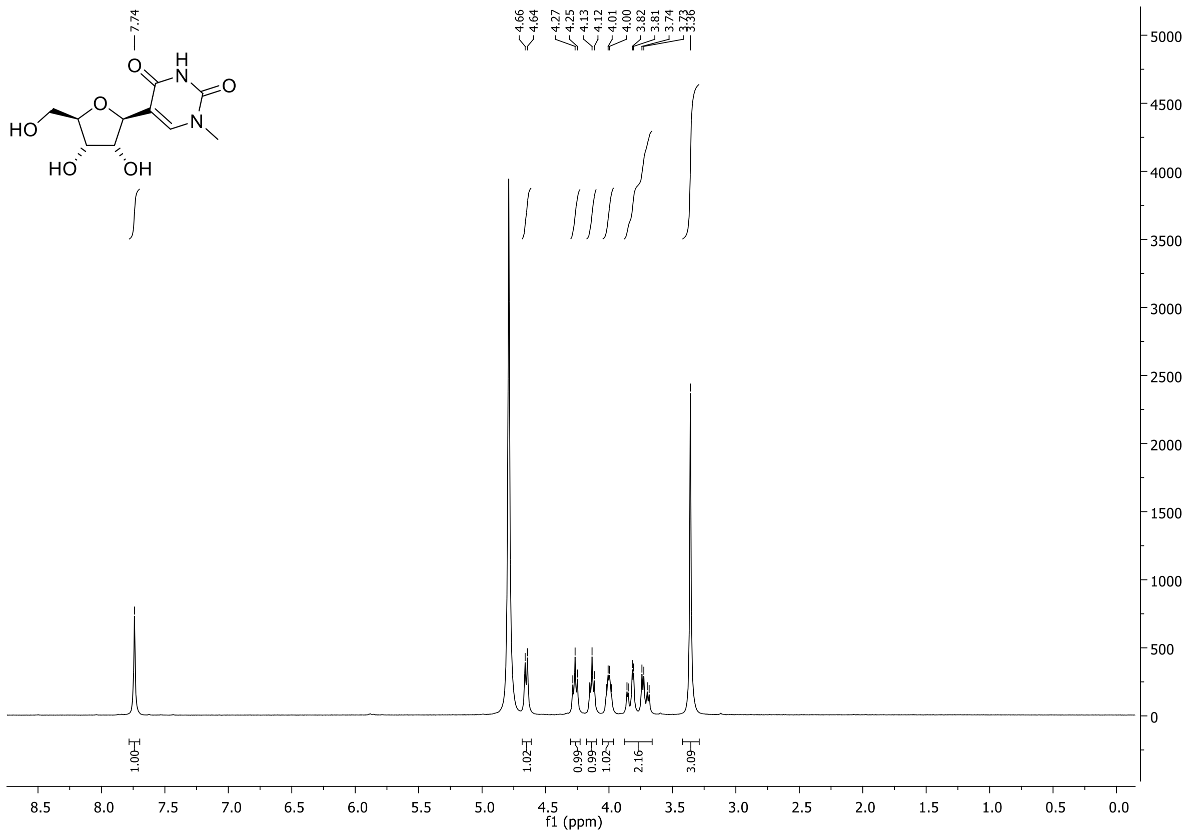

Figure S43. ^{1}H-NMR (300.36 MHz, D_2O) of m[1]Ψ (**3**).

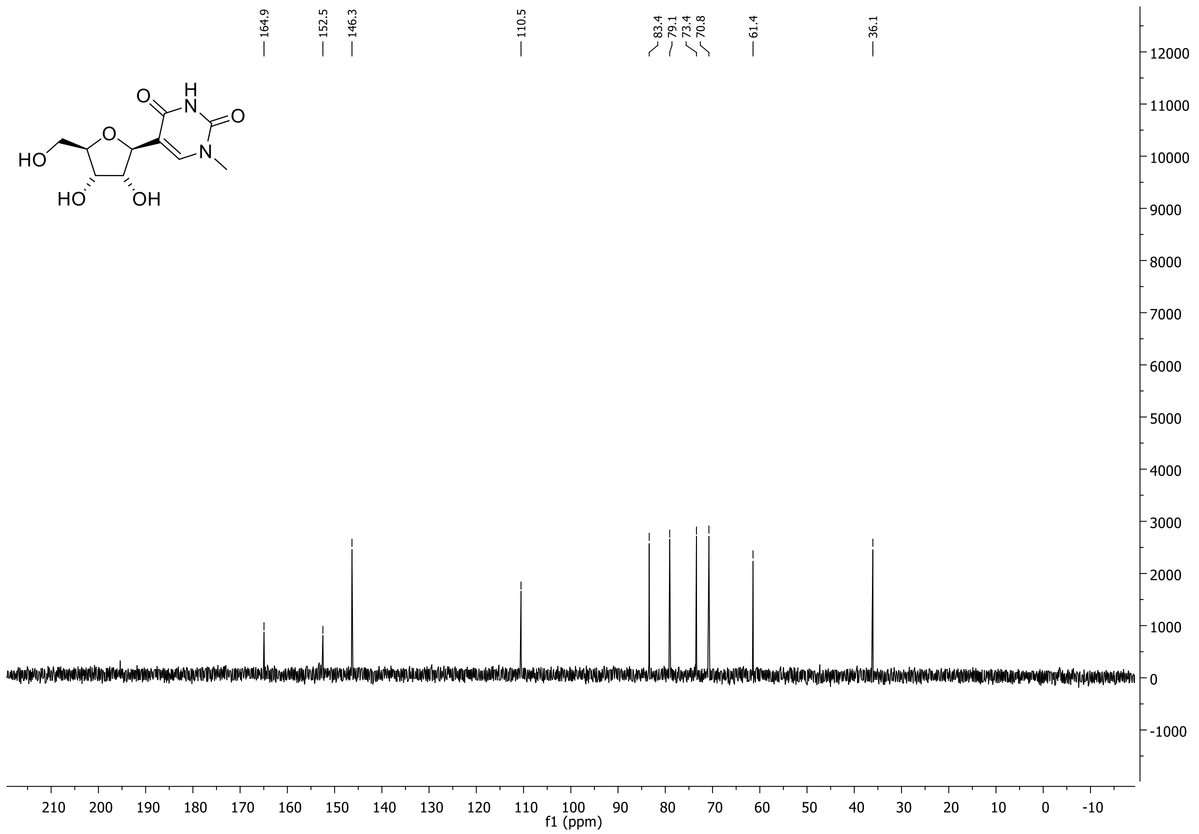

Figure S44. ^{13}C-NMR (75.53 MHz, D_2O) of m$^1\Psi$ (**3**).

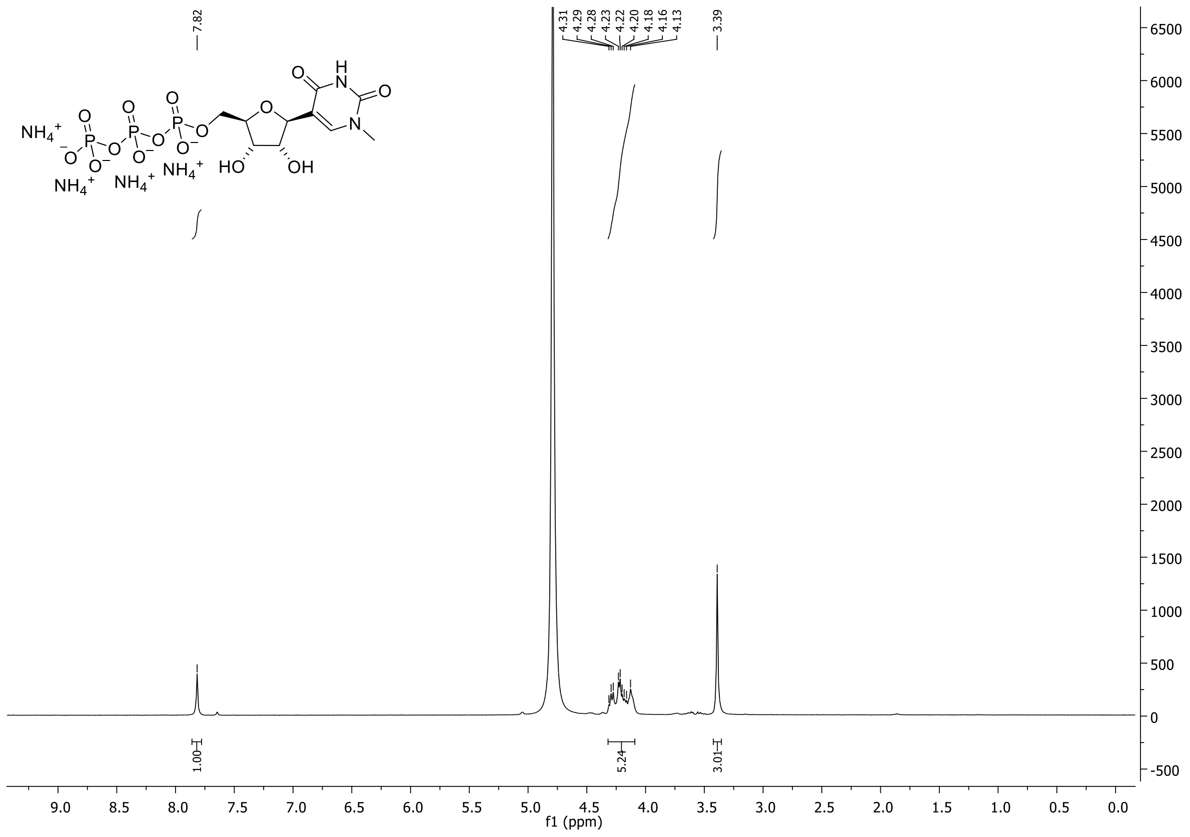

Figure S45. ^{1}H-NMR (300.36 MHz, D_2O) of m$^1\Psi$TP (**3c**) produced by the chemical route as presented in Scheme 2B.

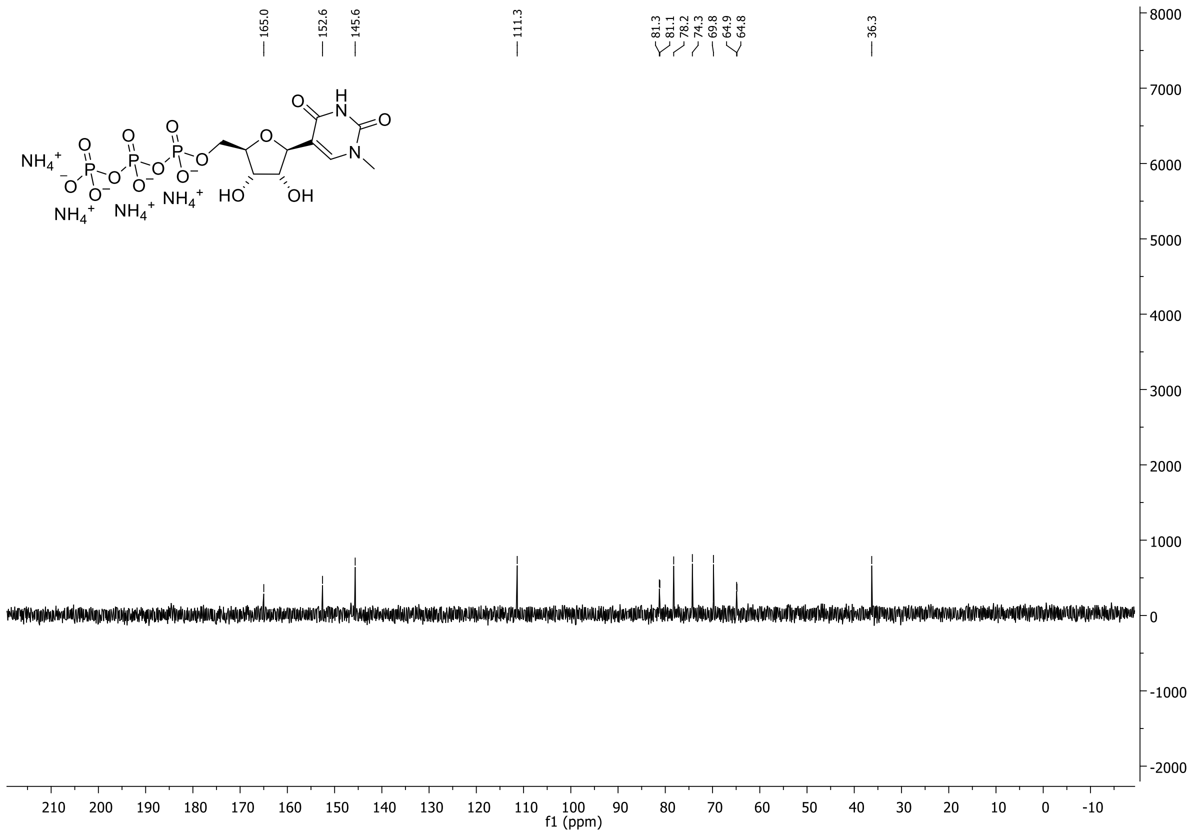

Figure S46. ^{13}C-NMR (75.53 MHz, D_2O) of m^{1}ΨTP (**3c**) produced by the chemical route as presented in Scheme 2B.

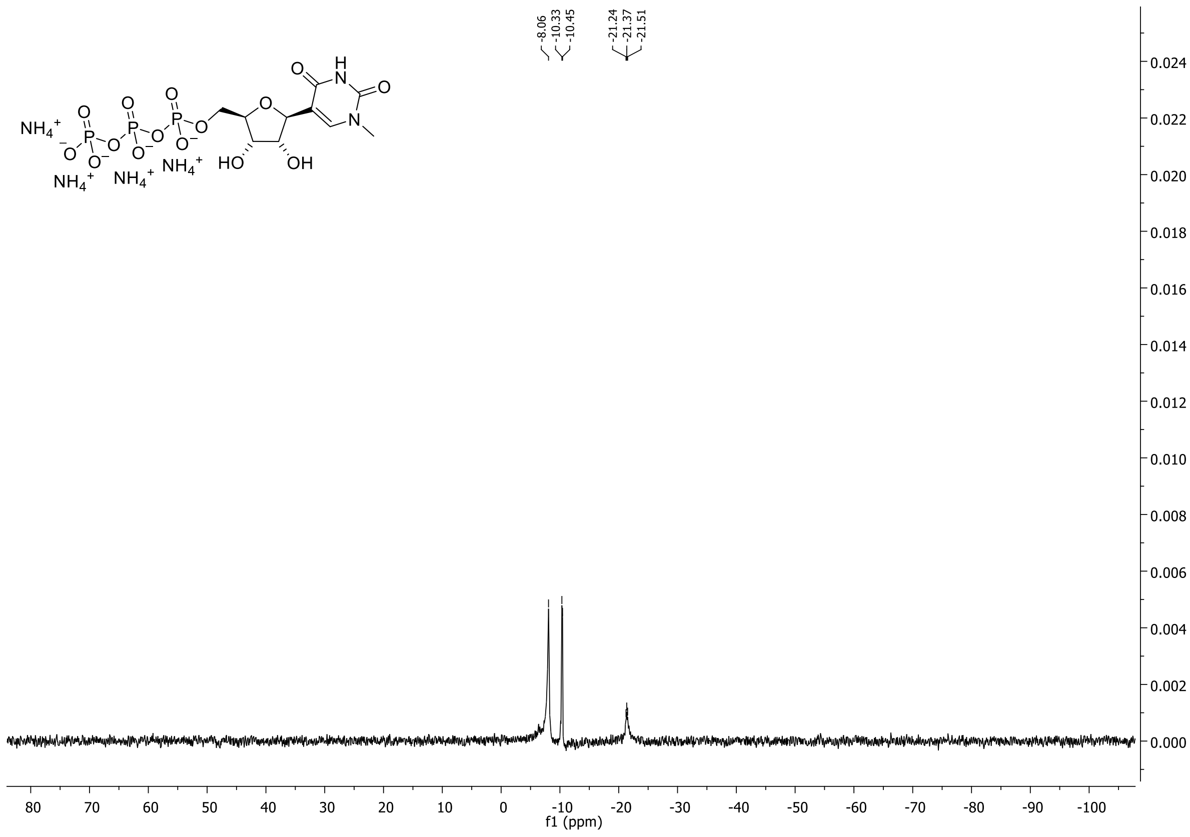

Figure S47. ^{31}P-NMR (161.83 MHz, D_2O) of m^{1}ΨTP (**3c**) produced by the chemical route as presented in Scheme 2B.

IVT appendix

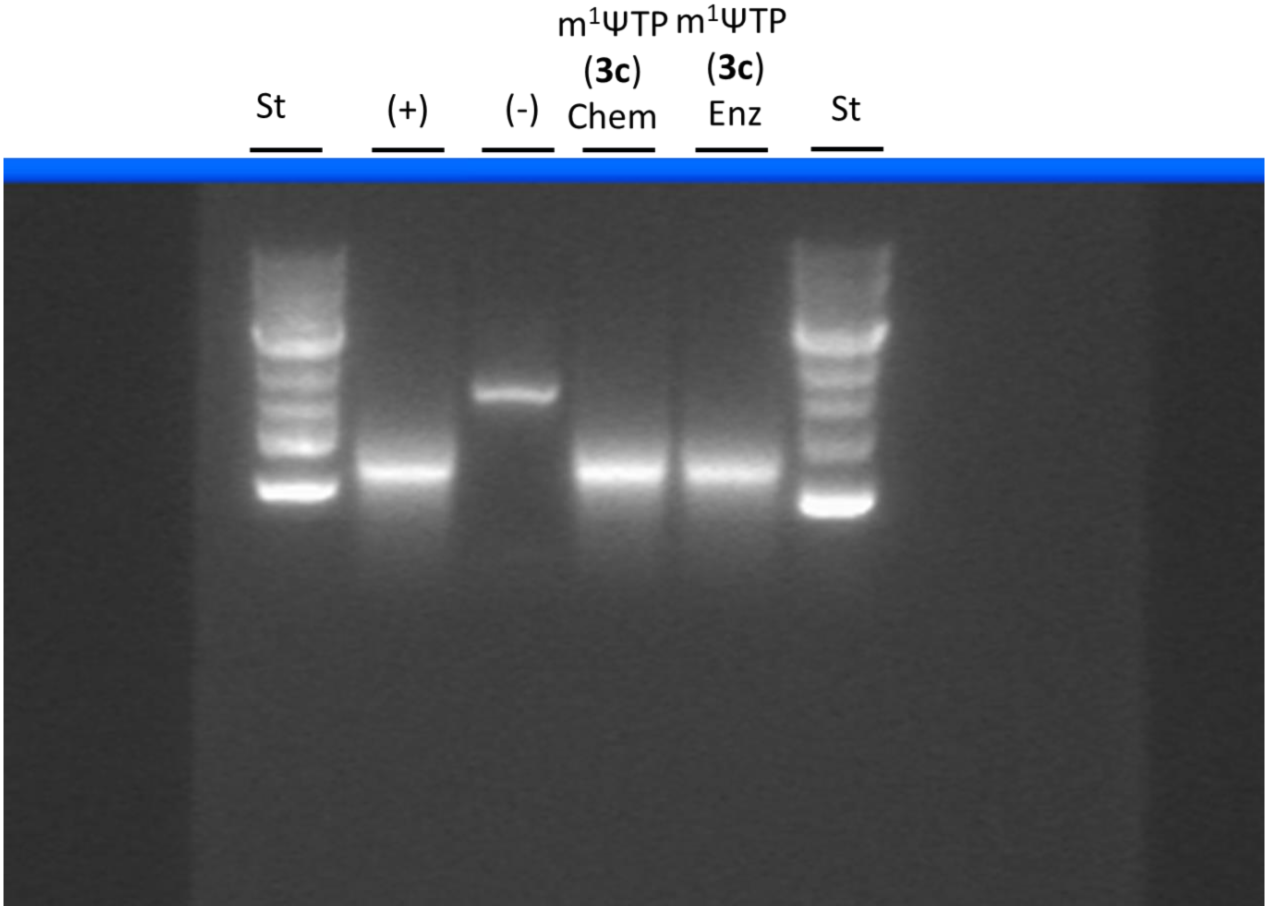

Figure S48. Analytical agarose gel of products from IVT with m^{1}ΨTP (**3c**) as substrate. m^{1}ΨTP (**3c**) was either synthesized chemoenzymatically (lane: Enz) or chemically (lane: Chem). Reactions were done with 2.0 mM of each ATP, CTP, GTP, UTP or UTP-replacing *C*-nucleotide, 1.0 µg linear DNA template (YeiN-encoding gene, Figure S50) and 1.2 *U* µL^{-1} RNA polymerase, incubated for 2 h at 37 °C. The positive control (+) used UTP, the negative control (-) lacked UTP. In reactions labeled m^{1}ΨTP (**3c**) Enz or Chem UTP was replaced with m^{1}ΨTP (**3c**). St, DNA ladder. This gel was used to generate Figure 3.

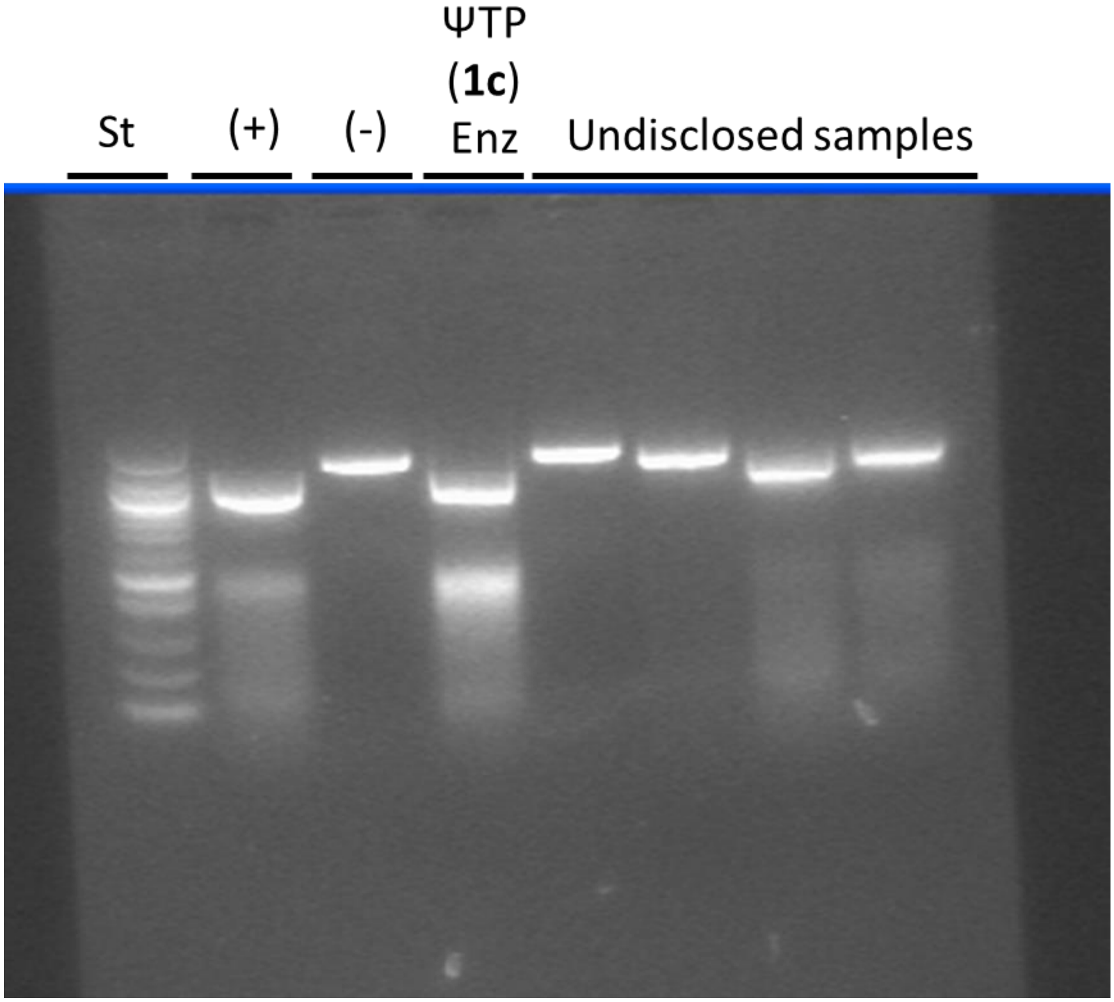

Figure S49. Analytical agarose gel of products from IVT with ΨTP (**1c**) as substrate. ΨTP (**1c**) was synthesized chemoenzymatically (lane: Enz). Reactions were done with 2.0 mM of each ATP, CTP, GTP, UTP or UTP-replacing *C*-nucleotide, 1.0 µg linear DNA template (YeiN-encoding gene, Figure S50) and 1.2 *U* μL^{-1} RNA polymerase, incubated for 2 h at 37 °C. The positive control (+) used UTP, the negative control (-) lacked UTP. In reaction ΨTP (**1c**) UTP was replaced by ΨTP (**1c**). Undisclosed samples are not addressed in this study. St, DNA ladder. This gel was used to generate Figure 3.

TAATACGACTCACTATAGGGgaattgtgagcggataacaattcccctctagaaataattttgtttaactttaagaaggagatatacc**ATGGGCAGCAGCCA**
TCATCATCATCATCACAGCAGCGGCCTGGTGCCGCGCGGCAGCCATATGTCTGAATTAAAAATTTCCCCTGAATTATTA
CAAATTTCCCCGGAAGTGCAGGACGCTTTAAAAAACAAAAAACCGGTTGTGGCGCTGGAATCGACCATTATTTCTCA
CGGGATGCCGTTCCCACAAAATGCCCAGACCGCAATTGAAGTTGAAGAAACTATTCGTAAACAGGGCGCTGTACCTG
CCACGATCGCCATTATTGGCGGCGTGATGAAAGTGGGGTTAAGCAAAGAAGAAATTGAATTACTGGGTCGTGAAGGG
CATAACGTGACCAAAGTTAGTCGTCGCGATTTACCTTTTGTTGTTGCCGCCGGAAAAAATGGCGCAACCACTGTGGCT
TCAACGATGATTATTGCGGCGCTTGCCGGAATTAAAGTATTTGCCACCGGGGGAATTGGTGGTGTGCATCGCGGGGC
GGAACATACCTTCGATATTTCTGCCGATTTGCAAGAACTGGCAAATACTAATGTCACCGTTGTTTGTGCCGGGGCGAA
ATCTATTCTCGATTTAGGATTAACCACTGAGTATTTAGAAACCTTCGGTGTGCCGTTAATTGGCTATCAGACTAAAGCG
CTGCCTGCGTTTTTCTGCCGCACCAGCCCGTTTGACGTCAGCATTCGTCTCGACAGCGCCAGCGAAATTGCCCGTGC
AATGGTGGTGAAATGGCAAAGCGGTCTGAACGGTGGCCTCGTGGTAGCGAACCCGATCCCGGAACAGTTTGCTATG
CCGGAACACACTATCAATGCGGCGATCGATCAGGCGGTAGCTGAAGCTGAAGCTCAGGGAGTCATTGGTAAAGAAA
GTACGCCATTCCTGCTGGCGCGCGTTGCTGAACTGACCGGCGGTGACAGCCTGAAATCCAACATCCAGCTGGTGTT
CAACAACGCCATTCTGGCGAGCGAAATTGCCAAAGAATATCAGCGTCTCGCGGGTTAActcgaggatccggctgctaacaaagcc
cgaaaggaagctgagttggctgctgccaCCGCTGAGCAATAACTAGC

Figure S50. Sequence of the DNA template used for IVT. T7 promotor (blue), Lac operator (orange), open reading frame (bold), T7-terminator (green), primer sequences (underlined).

References

[1] H. E. Gottlieb, V. Kotlyar, A. Nudelman, *J. Org. Chem.* **1997**, *62*, 7512–7515.

[2] E. Gasteiger, C. Hoogland, A. Gattiker, S. Duvaud, M. R. Wilkins, R. D. Appel, A. Bairoch in *The Proteomics Protocols Handbook* (Hrsg.: J. M. Walker), Humana Press, Totowa, NJ, **2005**, S. 571–607.

[3] L.-P. Wang, K. A. McKiernan, J. Gomes, K. A. Beauchamp, T. Head-Gordon, J. E. Rice, W. C. Swope, T. J. Martínez, V. S. Pande, *J. Phys. Chem. B* **2017**, *121*, 4023–4039.

[4] O. Trott, A. J. Olson, *J. Comput. Chem.* **2010**, *31*, 455–461.

[5] S. I. Maffioli, Y. Zhang, D. Degen, T. Carzaniga, G. Del Gatto, S. Serina, P. Monciardini, C. Mazzetti, P. Guglierame, G. Candiani et al., *Cell* **2017**, *169*, 1240-1248.e23.

[6] G. Tasnádi, W. Jud, M. Hall, K. Baldenius, K. Ditrich, K. Faber, *Adv. Synth. Catal.* **2018**, *360*, 2394–2401.
